# Lonely minds, inflamed guts: metabolic and circulating protein pathways linking social isolation and loneliness to inflammatory bowel disease

**DOI:** 10.1038/s41398-026-04116-0

**Published:** 2026-05-22

**Authors:** Jianhui Zhao, Jingyu Ye, Meng Zhang, Guirong Yu, Haosen Ji, Siyun Zhou, Fangyuan Jiang, Erxu Xue, Kangning Li, Ziqing Yu, Hong Yang, Hao Wu, Xue Li

**Affiliations:** 1 Center of Clinical Big Data and Analytics of The Second Affiliated Hospital, School of Public Health, Zhejiang University School of Medicine, Hangzhou, Zhejiang China; 2 Clinical and Translational Epidemiology Unit, Massachusetts General Hospital and Harvard Medical School, Boston, MA USA; 3 Division of Gastroenterology, Massachusetts General Hospital and Harvard Medical School, Boston, MA USA; 4 Nursing Department, Sir Run Run Shaw Hospital, Zhejiang University School of Medicine, Hangzhou, Zhejiang China; 5 Department of Gastroenterology, Peking Union Medical College Hospital, Chinese Academy of Medical Sciences and Peking Union Medical College, Beijing, China; 6 Department of Gastroenterology, National Clinical Research Center for Digestive Diseases, Changhai Hospital; National Key Laboratory of Immunity and Inflammation, Naval Medical University, Shanghai, China; 7 Zhejiang Key Laboratory of Intelligent Preventive Medicine, Hangzhou, Zhejiang China

**Keywords:** Psychology, Diseases

## Abstract

Mounting evidence suggests that psychosocial stressors, such as social isolation and loneliness, contribute to gastrointestinal disorders by disrupting gut-brain interactions. We aim to investigate the associations of social isolation, loneliness, and their related alterations in metabolism and circulating proteins with the risk of inflammatory bowel disease (IBD), including ulcerative colitis (UC) and Crohn’s disease (CD). 275,157 adults from the UK Biobank were analyzed, including metabolomic data from 68,362 participants and proteomic profiling from 29,339 participants. The exposures included social isolation, loneliness, and their related metabolites and circulating proteins, with incident IBD as the outcome. Cox regression and two-sample Mendelian randomization (TSMR) analysis were utilized to examine the associations. Over a mean follow-up of 13.49 years, this cohort study identified 1565 incident IBD cases, comprising 1063 UC cases and 492 CD cases. Social isolation and loneliness showed significant associations with an elevated risk of IBD (social isolation: hazard ratio [HR]_most isolated_: 1.31, 95%CI: 1.01-1.70; loneliness: 1.29, 95%CI: 1.04-1.60). Social isolation and loneliness jointly increased the risk of IBD by 85%, with an HR of 1.85 (95% CI: 1.02-3.36). TSMR analyses further indicated that more sports or gym activity reduced IBD and CD risk, more religious activity lowered UC risk, while fewer leisure/social activities increased UC risk. For the metabolomic analysis, eight and five metabolites were identified to be associated with social isolation and loneliness, respectively. Additionally, 22 circulating proteins consistently associated with both loneliness and social isolation were identified, predominantly enriched in cytokine-related pathways. The derived protein scores were positively associated with an increased risk of IBD. This study demonstrates social isolation and loneliness significantly raise IBD risk, with related metabolite and circulating proteins shedding light on underlying biological mechanisms.

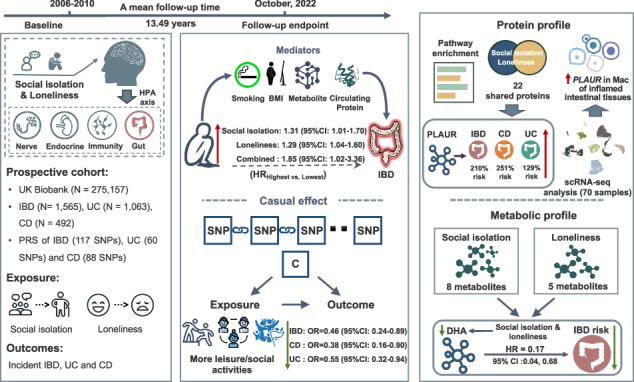

## Introduction

Inflammatory bowel disease (IBD), including ulcerative colitis (UC) and Crohn’s disease (CD), has a high global prevalence. At the turn of the 21st century, IBD emerged as a global disease, with its incidence accelerating in newly industrialized countries undergoing rapid Westernization [1]. The evolving epidemiology of IBD presents global challenges extending beyond medical research to include disease diagnosis, prevention, and health-care delivery systems [2].

Social isolation and loneliness, as behavioral and psychosocial risk factors, are gaining global attention for their impact on health and medical burden [3]. Social isolation and loneliness, reflecting objective and subjective deficits in social connection, are both linked to adverse physical and psychological health outcomes [3–5]. According to a meta-analysis, approximately 25% of community-dwelling older adults worldwide experience social isolation [6]. In European countries, the estimated prevalence of loneliness among adults aged 18-59 years ranged from 1.8 to 12%, and older adults from 4.2 to 24.2% [7]. Previous studies have demonstrated that loneliness and social isolation are associated with activation of the hypothalamic-pituitary-adrenal axis (HPA) and the sympathetic nervous system, which can lead to glucocorticoid resistance, upregulation of pro-inflammatory gene expression, and oxidative stress, all of which may contribute to the development and progression of IBD [8–10]. Although social isolation has been linked to increased mortality and exacerbated disease activity in IBD [11, 12], the associations of both social isolation and loneliness with the risk of incident IBD remain unclear. Previous studies have reported associations between loneliness and social isolation and various metabolic disorders [13], indicating their potential impact on human metabolism and metabolite alterations that may also contribute to IBD risk. Beyond social isolation and loneliness, accumulating evidence indicates that low social support, socioeconomic adversity, psychological distress, depression, anxiety, and chronic stress are also associated with increased IBD susceptibility and worse disease-related outcomes [14–16]. Additionally, patients with IBD experience a substantial burden of psychiatric comorbidities, particularly mood and anxiety disorders, underscoring the close interplay between mental health and chronic intestinal inflammation [17]. The co-regulation between inflammation and social behavior has been widely reported in the literature [18]. Social isolation and loneliness, as psychosocial stressors, can trigger stress responses involving the sympathetic nervous system (SNS) and HPA axis, leading to altered immune function and enhanced pro-inflammatory activity [19, 20]. A study by Shen et al. demonstrated associations between proteins linked to social isolation and loneliness and the risk of cardiovascular disease, stroke, and mortality [21]. However, given the close gut-brain interactions, no prior research has explored the associations between metabolites and proteins related to social isolation and loneliness and gut diseases such as IBD.

Herein, we investigated the associations between social isolation, loneliness, and the risk of IBD in a large-scale prospective cohort and conducted two-sample Mendelian randomization (TSMR) analyses to assess potential causal relationships. We further evaluated the impact of social isolation and loneliness on metabolomics and circulating proteins to elucidate their associations with IBD risk, thereby enriching IBD prevention strategies through a more nuanced understanding of social and psychological dimensions.

## Methods

### Study design and participants

A prospective cohort studies are based on data from the UK Biobank (UKB) database. The UKB is a comprehensive biomedical database of population health and genetic research resources. Between 2006 and 2010, more than 500,000 participants were recruited from 22 assessment centers across the UK. At the time of recruitment, participants filled out touchscreen questionnaires, underwent physical measurements, and provided blood samples [22]. The North West Multicenter Research Ethics Committee approved the study (REC reference for UK Biobank 11/NW/0382), and written informed consent was obtained from all participants [22]. In this cohort, after excluding participants with missing exposure or genetic data, pre-existing or early-onset IBD (within 1 year), and incomplete covariates to reduce potential reverse causality, a total of 275,157 participants were included in the primary analyses, with metabolomic and proteomic data available for 68,362 and 29,339 participants, respectively (Supplementary Figure S1). In the analysis for UC and CD, we excluded 501 and 1072 new cases of CD and UC, respectively.

### Assessment of exposure

The focal exposures in this study are social isolation and loneliness. Social isolation is primarily assessed through objective measures of social connections, including factors such as the frequency of social interactions or engagement in social activities [3]. And the evaluation of loneliness centers around the subjective perception of isolation [23]. Social isolation was assessed by considering factors such as the number of people in the household, the frequency of social activities, and contact with others. Loneliness, on the other hand, was evaluated based on subjective feelings of loneliness and the willingness to confide in others. Social isolation and loneliness were assessed using responses to questionnaire items. Social isolation was quantified by summing the scores of three items (range: 0-3) and categorized into three groups: least isolated (0), moderately isolated (1), and most isolated (2-3). Loneliness was assessed by summing the scores of two items (range: 0-2) and dichotomized into no loneliness (0-1) and loneliness (2). These assessment approaches were consistent with previously published studies on social isolation and loneliness, with detailed definitions provided in the Supplementary Methods [24].

### Assessment of outcome and covariates

Individuals with IBD were identified via self-report, primary care, and hospital inpatient data recorded (Hospital Episode Statistics for England, Scottish Morbidity Record, and Patient Episode Database for Wales) (Supplementary Table S1). The follow-up time was determined from the baseline date (date of visiting the assessment centre) to the diagnosis of IBD (including CD and UC), death, or follow-up endpoint (October 31, 2022 for England; August 31, 2022 for Scotland; and March 31, 2022 for Wales), whichever came first. Covariates for this study were collected at baseline, included age, sex, ethnicity, household income, educational level, employment status, smoking status, drinking status, physical activity, Townsend Deprivation Index (TDI), diet pattern, sleep pattern, body mass index (BMI) and Charlson Comorbidity Index (CCI). Physical activity was measured by the metabolic equivalent task (MET [MET score less than the cohort median or above the median]). A healthy sleep/diet pattern was defined as a healthy sleep/diet score ≥4. Specific information on covariates in the cohort and the field ID of the questions above in the UKB are presented in Supplementary Table S2. Additionally, the process of constructing the polygenic risk score (PRS) is as follows: after removing genetic variants in linkage disequilibrium, a total of 117, 88 and 60 independent single-nucleotide polymorphisms (SNP) (r^2^ < 0.01) at genome-wide significance (P < 5×10^−8^) were used to calculate polygenic scores for IBD, CD and UC, respectively (Supplementary Tables S3-S5). Grades of IBD genetic risk were defined as low, intermediate, and high according to the first quartile (Q1), second and third quartiles (Q2-Q3), and fourth quartile (Q4) of the PRS for IBD.

### Mendelian randomization

Our study utilized TSMR approach using summary-level data from genome-wide association studies (GWAS) conducted on individuals of European ancestry. The exposures of MR analysis included all items of social isolation and loneliness, with instrumental variables derived from the UKB GWAS. To avoid potential pleiotropy, outcome GWAS was extracted from a nonoverlapping European ancestry GWAS study (cases/controls for IBD: 25,042/34,915; UC: 12,366/33,609; CD: 12,194/28,072). Detailed information on the MR design is presented in Supplementary Methods.

### Metabolome and proteome

Baseline plasma samples, obtained at the time of participant enrollment in the UK Biobank (2006-2010), were utilized for high-throughput nuclear magnetic resonance (NMR) metabolomic profiling and plasma proteome. In contrast to the timing of sample collection, metabolomic profiling was performed in two distinct phases—Phase 1 (June 2019 to April 2020) and Phase 2 (April 2020 to June 2022)—utilizing state-of-the-art spectrometric techniques in Finland. The process involved comprehensive profiling procedures and rigorous quality control measures, as previously described [25, 26]. The biomarkers investigated span a range of metabolic pathways, including lipoprotein lipids across 14 subclasses, fatty acid compositions, fatty acids, and low-molecular-weight metabolites. Among the 249 metabolic measures, 168 metabolites in absolute levels were included in our analysis (excluding 81 ratio-based measures). Large-scale plasma proteome study constitutes a key component of the UK Biobank Pharma Proteomics Project (UKB-PPP). Detailed information regarding proteomics techniques, as well as normalization and quality control steps, has been published [27, 28]. In summary, a total of 2941 plasma protein analytes corresponding to 2923 unique proteins were measured using the antibody-based Olink Explore^TM^ Proximity Extension Assay (PEA) technology. Protein data are presented in log2 normalized protein expression (NPX) units from Olink.

### Statistical analyses

Baseline variates were presented as means ± standard error or median (interquartile range) for continuous variables and frequency (percentages) for categorical variables. Continuous variables were assessed for statistical differences using two-sample T-tests, ANOVA tests, or Mann-Whitney U tests. Categorical variables were evaluated for differences chi-squared test. The association of exposure (social isolation and loneliness) with incident IBD was assessed in multivariable Cox proportional hazards and presented as hazard ratio (HR) with 95% confidence interval (CI). Three models were generated for the analysis: Model 1 adjusted for age, sex, and ethnicity; Model 2 further adjusted for BMI, household income, educational level, employment status, smoking status, alcohol consumption, physical activity, TDI, healthy diet pattern, and healthy sleep pattern; Model 3 additionally adjusted for PRS of IBD, CD, or UC. The P values of the proportional hazard’s assumption for the Cox models for the global models, exceeded 0.05. The population attributable fraction (PAF) was calculated to reflect the proportion of the events that could be avoided by eliminating the exposure. In addition, the associations of loneliness and social isolation with IBD were further explored in participants with different levels of genetic susceptibility to IBD (low, intermediate, and high genetic risk).

Beyond the analyses above, we conducted several supplementary analyses to enhance the comprehensiveness of our study. In supplementary analyses, the cumulative risk of incident IBD among different groups is presented in Kaplan-Meier curves with a log-rank test. Furthermore, we explored potential interactions between social isolation and loneliness in relation to IBD risk. The separate and joint associations of social isolation and loneliness with the risk of IBD, the interaction between social isolation and loneliness in the risk of IBD, and the validation for their associations among participants with different levels of genetic susceptibility to IBD were further investigated. Furthermore, we performed subgroups for the separate and joint associations of social isolation and loneliness with IBD risk. Subgroup analyses were performed by stratifying by sex (male or female) and ethnicity (exclude non-europeans). In sensitivity analyses, we validated results in new models with further adjustment for CCI. And we further performed mediation analyses with bootstrapping (1000 replications) to confirm significant mediating covariates between social isolation, loneliness, and IBD incidence.

In MR analysis, we used odds ratios (OR) and 95% CI to estimate the IBD (CD and UC) risk caused by increased levels of loneliness and social isolation. For the primary MR analysis, we utilized the multiplicative random-effects inverse-variance weighted (IVW) method to estimate the causal effect [29]. Sensitivity analyses were performed using four MR methods: fixed-effects inverse-variance weighted, MR-Egger, penalized weighted median, and weighted median approaches. Cochran’s Q statistic was utilized to assess heterogeneity among the included IVs. A significance level of P < 0.05 indicated substantial heterogeneity. To account for pleiotropy effects, we conducted the MR-Radial and MR-PRESSO test by removing pleiotropic SNP associated with exposures and generating outlier-corrected results. Additionally, we performed a reverse TSMR analysis. Additional details on the MR analyses are presented in Supplementary method.

Linear regression was used to assess the associations of metabolites and circulating proteins with loneliness and social isolation. For metabolites, those associated with social isolation after false discovery rate (FDR) correction were further selected using Least Absolute Shrinkage and Selection Operator (LASSO) regression. For circulating proteins, Kyoto Encyclopedia of Genes and Genomes (KEGG) enrichment analyses were performed on proteins significantly associated with loneliness and social isolation after FDR correction. FDR correction was conducted independently for the IBD, UC, and CD analyses. The proteins significantly associated with both loneliness and social isolation were intersected, and protein scores for loneliness and social isolation were constructed by weighting these proteins according to their regression coefficients. During the construction of protein scores, GLIPR1, NPM1, and PCOLCE were excluded due to over 50% missing values, and the remaining missing protein values were imputed using the k-nearest neighbors algorithm from the R package ‘impute’. Cox proportional hazards regression models were then applied to evaluate the associations of loneliness- and social isolation–related metabolites, proteins, and protein scores with the risk of IBD, including UC and CD. All linear and Cox regression models were adjusted according to Model 3.

All statistical analyses were performed using R (version 4.2.1). A two-sided P value of less than 0.05 was considered indicative of statistical significance.

### Bioinformatics analysis

We collected bulk RNA-sequencing data from both peripheral blood and intestinal tissues of patients with IBD from the Gene Expression Omnibus (GEO) database, including GSE193677 (intestinal tissues, n = 2490) and GSE186507 (peripheral blood, n = 1030). Based on endoscopic evaluation, intestinal tissue samples were classified into five groups: UC inflamed tissues (UC-IT), UC non-inflamed tissues (UC-NIT), CD inflamed tissues (CD-IT), CD non-inflamed tissues (CD-NIT), and healthy intestinal tissues (Normal). Raw count data were transformed using log (count + 1), and differences in gene expression levels among groups were assessed using the Wilcoxon test.

We analyzed 70 single-cell RNA-seq samples from four GEO datasets, including UC, CD, UC-NIT, CD-NIT, and normal tissues. After standard preprocessing, doublet removal, and data integration using Seurat and Harmony, 230,075 high-quality cells were retained, clustered, and annotated into seven major cell types. Subclusters with high target gene expression were identified, followed by differential expression and Gene Ontology enrichment analyses within immune cell populations to characterize cell-type–specific expression patterns and functional pathways associated with the target gene in UC and CD. Detailed single-cell analyses are presented in the Supplementary Methods.

## Results

### Baseline characteristics

The average age of the cohort population was 55.86 years, and 48.7% of the participants were male. The baseline characteristic of participants was presented in Supplementary Table S6, respectively. Individuals with higher levels of social isolation or loneliness were more likely to be male, obese, smokers, physically inactive, and to have unhealthy dietary and sleep patterns.

### Social isolation, loneliness, and IBD risk

1565, 1063 and 492 participants were diagnosed with IBD, UC and CD during a mean follow-up period of 13.49 years, respectively. Compared with participants in the least isolated group, those in the moderately and most isolated groups had a significantly higher risk of IBD (moderately isolated: HR = 1.13, 95% CI: 1.02, 1.26; most isolated: HR = 1.31, 95% CI: 1.01, 1.70; P for trend = 0.004) (Table 1). Similarly, individuals reporting loneliness had a significantly higher risk of IBD than those without loneliness (HR 1.29, 95%CI: 1.04, 1.60). In the joint effect analysis, with increasing levels of both social isolation and loneliness, participants had a progressively and significantly higher risk of IBD (P trend < 0.001) (Table 1 and Supplementary Table S7). When participants experience both isolation and loneliness simultaneously, the risk of developing IBD significantly increases in both moderately (HR = 1.53, 95%CI: 1.12, 2.09) and most isolated groups (HR = 1.85, 95%CI: 1.02, 3.36). Participants experiencing loneliness have a higher risk of developing UC compared to those without loneliness (HR = 1.39, 95%CI: 1.08, 1.79), and the association between high social isolation and UC incidence is on the edge of statistical significance (P trend = 0.053) (Supplementary Table S8). Meanwhile, when loneliness and isolation (most isolated) coexist, the risk of developing UC was 2.27 times higher (95% CI: 1.17, 4.40) compared to participants without loneliness and isolation. Additionally, social isolation showed a positive trend with the risk of CD onset (P for trend = 0.029) (Supplementary Table S8).

**Table 1:** Separate and joint association of social isolation and loneliness with long-term risk of IBD.

Exposure	N	Cases/Person-years	Model 1 HR (95% CI)	Model 2 HR (95% CI)	Model 3 HR (95% CI)	PAF (%) (95% CI)
**Separate effects**						
Social isolation						6.09 (4.25-7.93)
Least isolated	182913	977/2472341	1.00 [Reference]	1.00 [Reference]	1.00 [Reference]	
Moderately isolated	83945	526/1128210	1.18 (1.06-1.32)	1.13 (1.02-1.26)	1.13 (1.02-1.26)	
Most isolated	8299	62/110667	1.39 (1.08-1.8)	1.31 (1.01-1.7)	1.31 (1.01-1.70)	
**P trend**			<0.001	0.004	0.004	
Loneliness						1.96 (1.35-2.57)
No loneliness	264338	1474/3566371	1.00 [Reference]	1.00 [Reference]	1.00 [Reference]	
Loneliness	10819	91/144846	1.5 (1.22-1.86)	1.30 (1.05-1.61)	1.29 (1.04-1.60)	
**Joint effects**						
Absence of loneliness						
Least isolated	177335	939/2397130	1.00 [Reference]	1.00 [Reference]	1.00 [Reference]	
Moderately isolated	79627	484/79627	1.16 (1.04-1.29)	1.12 (1-1.25)	1.11 (1-1.24)	
Most isolated	7376	51/98513	1.3 (0.98-1.72)	1.25 (0.94-1.66)	1.25 (0.94-1.66)	
Loneliness						
Least isolated	5578	38/75210	1.27 (0.92-1.76)	1.12 (0.81-1.56)	1.12 (0.81-1.55)	
Moderately isolated	4318	42/57482	1.86 (1.37-2.53)	1.55 (1.14-2.12)	1.53 (1.12-2.09)	
Most isolated	923	11/12153	2.29 (1.26-4.15)	1.85 (1.02-3.35)	1.85 (1.02-3.36)	
**P trend**			<0.001	<0.001	<0.001	

Model 1 adjusted for age, sex, and ethnicity; Model 2 further adjusted for BMI, household income, educational level, employment status, smoking status, alcohol consumption, physical activity, TDI, healthy diet pattern, and healthy sleep pattern; Model 3 additionally adjusted for PRS of IBD.

*BMI* body mass index, *HR* hazard ratio, *IBD* inflammatory bowel disease, *PAF* population attributed fraction, *PRS* polygenic risk score.

**Fig. 1:**
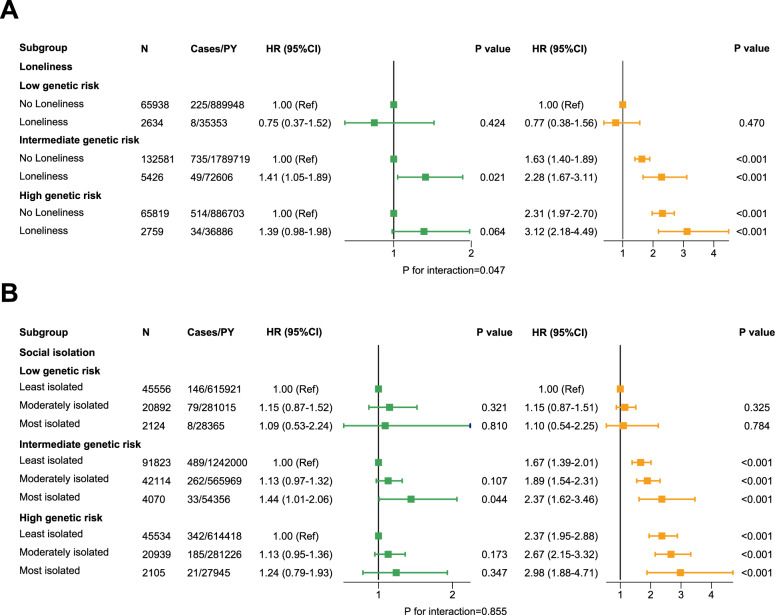
Genetic susceptibility on the impact of loneliness and social isolation on IBD risk. Separate and joint association of loneliness (**A**) and social isolation (**B**) with the long-term risk of IBD across different levels of genetic susceptibility. The interaction P-value for genetic risk subgroups and social isolation in IBD was 0.855, while for loneliness in IBD it was 0.047. These analyses were conducted using Model 2, which adjusted for age, sex, ethnicity, BMI, household income, education level, employment status, smoking status, alcohol consumption, physical activity, healthy diet pattern, and healthy sleep pattern. Genetic risk categories were defined as low, intermediate, and high based on quartiles of the PRS for IBD (quartile 1, quartiles 2–3, and quartile 4, respectively). Abbreviations: BMI body mass index, IBD inflammatory bowel disease, PRS polygenic risk score.

The associations of social isolation and loneliness with IBD risk across different genetic risk groups are shown in Fig. 1 and Supplementary Table S9. Among individuals with moderate genetic susceptibility, both social isolation and loneliness were associated with elevated IBD risk, with HRs of 1.44 (95% CI: 1.01-2.06) for the most isolated group and 1.41 (95% CI: 1.05-1.89) for loneliness. Additionally, the association for social isolation was not modified by genetic susceptibility (P interaction= 0.855), and association for loneliness was modified by genetic susceptibility (P interaction = 0.047). When stratifying for genetic susceptibility to UC, we observed that in individuals with moderate genetic risk for UC, both the most isolated (HR = 1.61, 95%CI: 1.07, 2.41) and those experiencing loneliness (HR = 1.84, 95%CI: 1.34, 2.54) showed an increased association with the risk of UC (Supplementary Figure S2). Stratified analyses by genetic susceptibility to CD showed that, among individuals with moderate genetic risk of CD, those in the most isolated group had a higher risk of incident CD compared with those in the least isolated group (HR = 1.97, 95% CI: 1.11, 3.50, Supplementary Figure S3).

### Metabolic changes related to social isolation and loneliness and their association with IBD risk

**Fig. 2:**
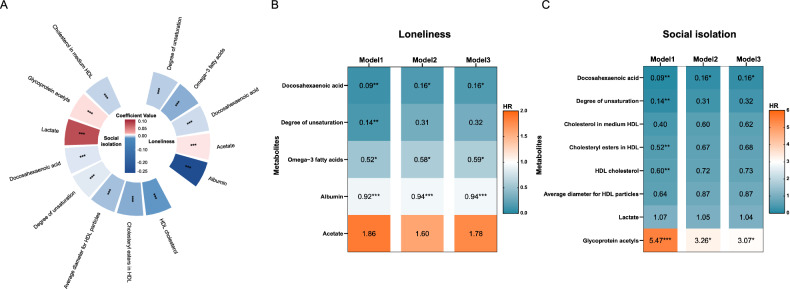
Associations between loneliness- and social isolation-related metabolites and the risk of IBD. Metabolites associated with social isolation and loneliness (**A**); associations of loneliness- (**B**) and social isolation-related (**C**) metabolites with the risk of IBD. The association analysis between metabolites and the risk of IBD was performed in Model 3. *, P < 0.05; **, P < 0.01; ***, P < 0.001. Abbreviations: CD Crohn’s disease, IBD inflammatory bowel disease, MR Mendelian randomization, UC ulcerative colitis.

Linear regression analyses identified 49 metabolites associated with loneliness, of which five remained significant after FDR correction (Supplementary Table S10 and Fig. 2). Among these, degree of unsaturation, omega-3 fatty acids, docosahexaenoic acid (DHA), and albumin were negatively associated with loneliness, whereas acetate showed a positive association. Importantly, DHA (HR = 0.17; 95% CI: 0.04, 0.68; P = 0.013), omega-3 fatty acids (HR = 0.59; 95% CI: 0.35, 0.98; P = 0.040), and albumin (HR = 0.94; 95% CI: 0.91, 0.97; P < 0.001) were independently associated with a significantly reduced risk of incident IBD (Fig. 2). For social isolation, linear regression analyses identified 115 associated metabolites, and eight remained significant after FDR correction (Supplementary Table S10 and Fig. 2). Among these, DHA, degree of unsaturation, cholesterol in medium high-density lipoprotein (HDL), cholesteryl esters in HDL, HDL cholesterol, average diameter for HDL particles, and lactate were negatively associated with social isolation, whereas glycoprotein acetyls were positively associated. Notably, DHA was significantly associated with a reduced risk of incident IBD, while glycoprotein acetyls (HR = 3.07; 95% CI: 1.24, 7.56; P = 0.015) were linked to an increased risk (Fig. 2). The associations of these metabolites with UC and CD are shown in Supplementary Figure S4. Among loneliness-related metabolites, albumin showed a significant inverse association with the risk of both UC and CD. In contrast, no social isolation–related metabolites were significantly associated with UC risk. However, in CD, higher levels of cholesterol in medium HDL, cholesteryl esters in HDL, and HDL cholesterol were all significantly associated with a lower risk, whereas glycoprotein acetyls were significantly positively associated with CD risk.

### Proteomic alterations associated with social isolation and loneliness and their relationship to IBD risk

**Fig. 3:**
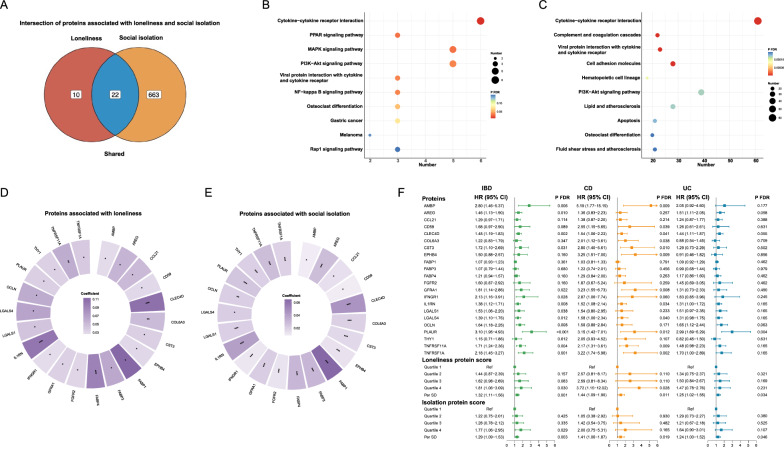
Associations between loneliness- and social isolation-related proteins and the risk of IBD. Overlap of proteins linked to both loneliness and social isolation (**A**); pathway enrichment analyses for proteins related to loneliness (**B**) and social isolation (**C**); correlation plots of circulating proteins with loneliness (**D**) and social isolation (**E**); associations between circulating proteins and IBD, CD, and UC (**F**). The association analysis between circulating proteins and the risk of IBD was performed in Model 3. *, P < 0.05; **, P < 0.01; ***, P < 0.001. Abbreviations: BMI body mass index, CD Crohn’s disease, IBD inflammatory bowel disease, PRS polygenic risk score, UC ulcerative colitis.

A total of 685 and 32 circulating proteins were identified to be significantly associated with social isolation and loneliness, respectively (P_FDR_ < 0.05) (Fig. 3A and Supplementary Tables S11-S12). KEGG enrichment analysis revealed that proteins related to social isolation were mainly enriched in signaling pathways such as cytokine–cytokine receptor interaction, PPAR signaling, MAPK signaling, PI3K-Akt signaling, viral protein interaction with cytokine and cytokine receptor, and NF-kappa B signaling (Fig. 3B). Proteins associated with loneliness were enriched in cytokine-cytokine receptor interaction, complement and coagulation cascades, viral protein interaction with cytokine and cytokine receptor, cell adhesion molecules, hematopoietic cell lineage, PI3K-Akt signaling, lipid and atherosclerosis, and apoptosis pathways (Fig. 3C). Notably, 22 circulating proteins were found to be consistently associated with both social isolation and loneliness (Fig. 3D-E). Protein scores constructed based on these 22 proteins were positively associated with increased IBD risk, with the loneliness protein score showing an HR per standard deviation (SD) of 1.32 (95% CI: 1.11, 1.56) and the social isolation protein score showing an HR per score of 1.29 (95% CI: 1.09, 1.53). Specifically, after FDR correction, elevated circulating levels of AMBP, CLEC4D, CST3, GFRA1, IFNGR1, IL1RN, LGALS1, LGALS4, OCLN, PLAUR, TNFRSF11A, and TNFRSF1A were significantly associated with an increased risk of IBD. AMBP, CLEC4D, COL6A3, CST3, EPHB4, GFRA1, IL1RN, LGALS4, PLAUR, TNFRSF11A, and TNFRSF1A were associated with an increased risk of CD. PLAUR was significantly associated with an increased risk of UC (Fig. 3F). Among them, PLAUR was consistently associated with increased risks of IBD, CD, and UC, with PLAUR showing HRs of 3.10 (95% CI: 1.95, 4.93; P < 0.001) for IBD, 3.15 (95% CI: 1.42, 7.01; P = 0.012) for CD, and 2.99 (95% CI: 1.69, 5.29; P = 0.004) for UC.

### PLAUR transcriptionally associated with UC and CD

**Fig. 4:**
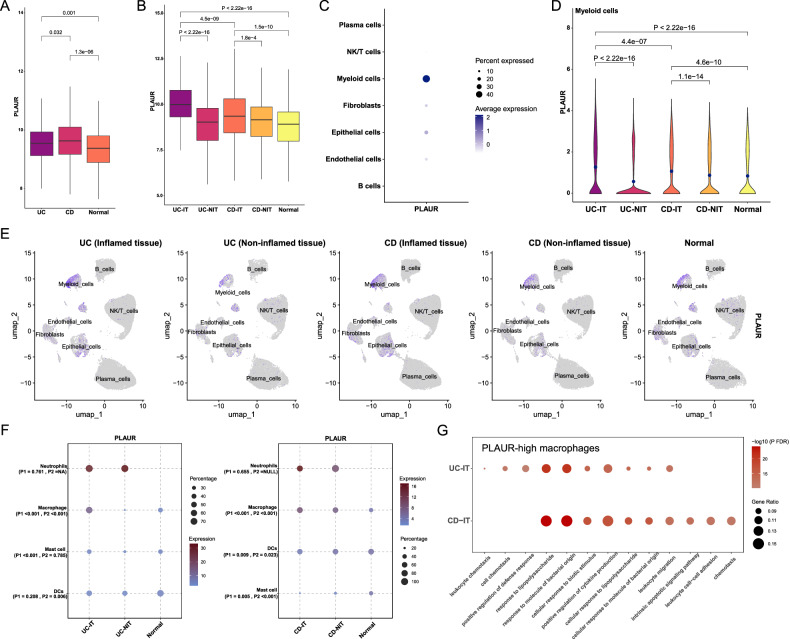
Transcriptomic characterization of *PLAUR* in IBD. Transcriptomic levels of *PLAUR* in peripheral blood among UC, CD patients, and healthy controls (**A**); *PLAUR* expression in intestinal tissues across UC, CD, and controls (**B**); cell-type–specific expression of *PLAUR* in intestinal tissues (**C**); *PLAUR* expression in intestinal myeloid cells (**D**); cellular distribution of PLAUR based on UMAP clustering of intestinal tissues (**E**); *PLAUR* expression across different intestinal myeloid cell subsets (**F**); pathway enrichment of upregulated genes in *PLAUR*-high macrophages from inflamed UC and CD tissues (**G**). Abbreviations: CD Crohn’s disease, FDR false discovery rate, IBD inflammatory bowel disease, IT inflamed tissues, NIT non-inflamed tissues, UC ulcerative colitis.

Based on the evidence from circulating protein studies, we further investigated the role of PLAUR in IBD at the transcriptomic level (Fig. 4). RNA-seq data showed that *PLAUR* expression was upregulated in the peripheral blood of both UC (P = 0.001) and CD (P < 0.001) patients compared to healthy controls. At the tissue level, *PLAUR* was significantly upregulated in inflamed intestinal tissues of UC and CD patients compared to non-inflamed and normal intestinal tissues. Single-cell transcriptomic analysis further revealed that *PLAUR* was highly expressed and abundant in myeloid cells. Notably, *PLAUR* expression in macrophages was significantly elevated in inflamed intestinal tissues of UC and CD compared to both normal and non-inflamed tissues (all P < 0.001). In inflamed intestinal tissues of UC and CD, *PLAUR*-high macrophages were predominantly enriched in pathways related to response to lipopolysaccharide, response to molecules of bacterial origin, cellular response to biotic stimulus, positive regulation of cytokine production, and leukocyte migration.

### Subgroups, sensitivity and mediation analyses

The results of stratified analysis by sex indicated that the impact of social isolation and loneliness on the incidence of IBD is more pronounced in males, with no significant statistical association observed in females (Supplementary Table S13). We further excluded cases of non-European descent and found that the association between social isolation and loneliness with the risk of IBD incidence remains significant (Supplementary Table S14). We further adjusted for CCI on the basis of Model 3 and found that social isolation and loneliness remain significantly associated with an increased risk of IBD (Supplementary Table S15). Furthermore, when conducting the analysis while retaining all IBD cases, the association between social isolation, loneliness, and the risk of IBD incidence is generally consistent with the main analysis (Supplementary Table S16). In mediation analyses, DHA (P = 0.026) and glycoprotein acetyls (P = 0.012) were identified as mediators of the association between social isolation and IBD risk (Supplementary Table S17). Additionally, omega-3 fatty acids (P = 0.050), docosahexaenoic acid (P = 0.012), and albumin (P < 0.001) mediated the relationship between loneliness and IBD risk. Additionally, the proteins AMBP, AREG, CLEC4D, CST3, GFRA1, IFNGR1, IL1RN, LGALS1, LGALS4, OCLN, PLAUR, TNFRSF11A, TNFRSF1A, as well as the protein feature score, were found to mediate the association between social isolation and IBD risk (Supplementary Table S18). Similarly, these proteins and the protein feature score also mediated the association between loneliness and IBD risk. In addition, BMI and smoking were identified as mediators in the associations between social isolation, loneliness, and IBD risk, with mediation proportions ranging from 5.83% to 8.19% (Supplementary Table S19).

### Mendelian randomization analysis of social isolation, loneliness and the risk of IBD

**Fig. 5:**
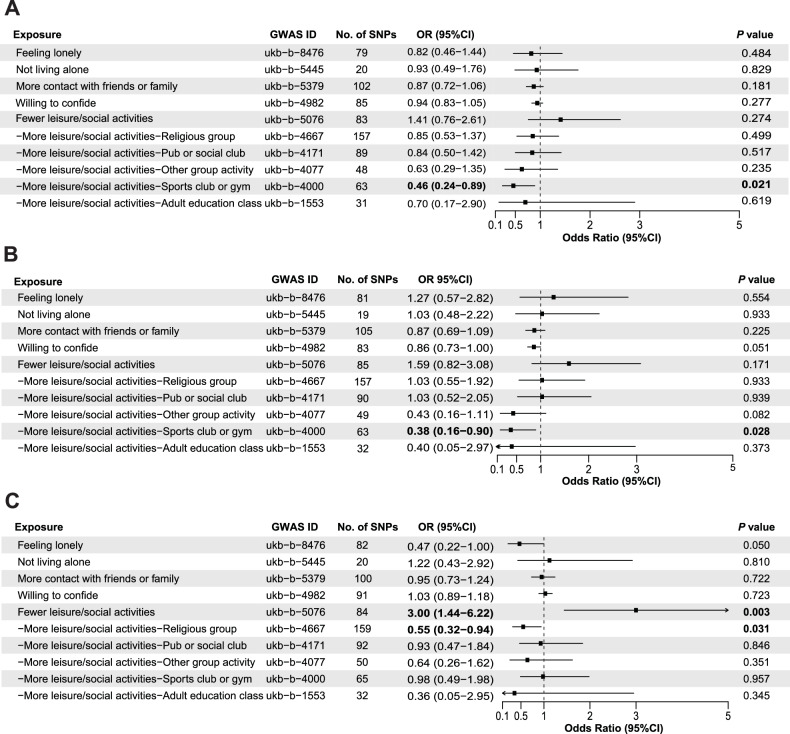
Causal association from loneliness and social isolation to IBD, CD, and UC. MR analyses for the causal effects of loneliness and social isolation on the risk of IBD (**A**), CD (**B**) and UC (**C**). Abbreviations: CD Crohn’s disease, IBD inflammatory bowel disease, MR Mendelian randomization, UC ulcerative colitis.

In the TSMR analyses, engaging in more activities-sports club or gym was associated with a reduced risk of IBD (OR = 0.46, 95%CI: 0.24, 0.89, P = 0.021) and CD (OR = 0.38, 95%CI: 0.16, 0.90, P = 0.028) (Fig. 5 and Supplementary Tables S20-S21). Furthermore, the analysis identified that participating in more religious activities (OR = 0.5, 95%CI: 0.32, 0.94, P = 0.031) were each associated with a decreased risk of UC, whereas engaging in fewer leisure social activities (OR = 3.00, 95%CI: 1.44, 6.22, P = 0.003) were each associated with an elevated risk of UC (Fig. 5 and Supplementary Table S22). Estimates from MR analyses with RE-IVW method and sensitivity analyses were shown in Supplementary Tables S23-S25. The results of the reverse MR were exhibited in Supplementary Table S26.

## Discussion

This prospective cohort study demonstrated that loneliness and social isolation were independently and jointly associated with an increased risk of developing IBD. TSMR analysis further supported a potential causal relationship between social isolation, loneliness, and the risk of IBD, including both UC and CD. Metabolomic analysis identified eight and five metabolites significantly associated with social isolation and loneliness, respectively. Notably, individuals experiencing social isolation or loneliness exhibited lower levels of DHA, which was associated with a reduced risk of IBD. Proteomic analysis revealed 22 circulating proteins consistently associated with both social isolation and loneliness, with enrichment in cytokine-related pathways. A composite protein score derived from these proteins was positively associated with an increased risk of IBD. Among these, PLAUR showed the strongest association with an elevated risk of IBD as well as both CD and UC subtypes. Transcriptomic data further demonstrated that PLAUR was highly expressed in both peripheral blood and inflamed intestinal tissues of UC and CD patients, particularly within macrophages. PLAUR-high macrophages were significantly enriched in pathways related to inflammation, immune cell activation, and migration. These findings suggest that social isolation and loneliness may promote IBD development via brain-immune-gut pathways, involving metabolic alterations in circulating protein profiles.

Our study findings suggest that social isolation and loneliness may elevate the risk of IBD. Specifically, after adjusting for the genetic risk of IBD, modifiable lifestyle factors, and other baseline characteristics, the risk of IBD increased by 13 and 31% for participants who were moderately isolated and most isolated, respectively, compared to the least isolated participants. Furthermore, individuals in the loneliness group experienced a 29% increase in the risk of developing IBD compared to the non-loneliness group. The characteristics of IBD involve chronic immune-mediated intestinal inflammation driven by a combination of genetic susceptibility, environmental factors, and microbial elements [30, 31]. The mechanisms through which social determinants of health contribute to variations in IBD are increasingly being recognized [32]. Acute and chronic social isolation elicits distinct physiological, psychological, and behavioral effects [33]. Acute social isolation activates the immune system, inducing upregulation of pro-inflammatory interleukin genes to safeguard individuals from the dangers of solitude [34]. Simultaneously, the hypothalamus-pituitary-adrenal axis, as another major target, is triggered, initiating the neuroendocrine response [35]. Furthermore, the central nervous system undergoes alterations, leading to a state of heightened vigilance and increased excitability [36]. Although these changes may be adaptive in the short term, the consequences of prolonged isolation often result in maladaptive outcomes [37, 38]. In addition, perceived loneliness is linked to an inflammatory response [39]. Interestingly, the association between loneliness and IBD risk was most pronounced in individuals with moderate genetic susceptibility, with a significant interaction observed. This pattern may reflect differences in the relative contribution of genetic and psychosocial factors across risk strata, although caution is warranted given the larger sample size in the moderate genetic risk group (PRS Q2-Q3).

Beyond confirming the association between social isolation, loneliness, and IBD in cohort studies, we for the first time applied TSMR to provide stronger evidence supporting a causal relationship. Our results indicated that engaging in fewer leisure social activities was associated with an increased risk of UC, while participating and more activities such as sports clubs or gyms was associated with a reduced risk of IBD and its subtypes. Holt-Lunstad and Perissinotto underscored integrating responses to patients’ social needs into direct care to enhance treatment outcomes, proposing the Educate, Assess, Respond (EAR) framework for addressing social isolation and loneliness [40]. Guided by this framework, clinicians educate patients to promote risk-reducing behaviors, routinely assess social isolation and loneliness, document these factors in health records, and tailor coping strategies to patients’ evolving needs. Regarding specific interventions, social prescribing programs that link primary care patients to nonmedical community resources may offer an effective approach to improving patient well-being [41]. Unlike objective social isolation, loneliness is a subjective experience and requires behavioral and psychological interventions to enhance perceived social connection. Among available strategies, internet-based cognitive behavioral therapy and other digital or tele-delivered interventions represent particularly promising and scalable approaches [42].

From a metabolomic perspective, our study found that individuals experiencing loneliness had lower levels of omega-3 fatty acids, DHA and albumin, all of which showed significant protective associations with IBD risk. In socially isolated individuals, glycoprotein acetyls were elevated and DHA was decreased; glycoprotein acetyls were found to be associated with an increased risk of IBD. Notably, individuals experiencing social isolation and loneliness consistently showed lower levels of DHA. Previous studies have shown that DHA accounts for more than 40% of total omega-3 polyunsaturated fatty acids in neuronal tissue, particularly in the gray matter [43]. DHA plays important roles in reducing inflammation and protecting cognitive function, and lonelier individuals have been observed to experience a greater decline in episodic memory over time, while omega-3 supplementation has been shown to mitigate loneliness-induced memory impairments [44]. On the other hand, epidemiological studies suggest that higher dietary intake of DHA is associated with a reduced risk of IBD [45]. Animal experiments further support this finding, demonstrating that DHA can alleviate colitis by protecting intestinal barrier integrity through its antioxidant and anti-inflammatory properties [46]. Additionally, glycoprotein acetyls, as a composite biomarker of inflammation, have been recognized as a reliable indicator of chronic systemic inflammation and have been positively associated with various inflammation-related non-communicable diseases [47]. This supports the hypothesis that socially isolated individuals may experience elevated levels of chronic inflammation, thereby contributing to an increased risk of IBD.

At the proteomic level, our study identified a set of proteins associated with social isolation, loneliness, and incident IBD, including AMBP, AREG, CLEC4D, CST3, GFRA1, IFNGR1, IL1RN, LGALS1, LGALS4, OCLN, PLAUR, TNFRSF11A, and TNFRSF1A, many of which are known for their roles in immune response and inflammatory processes. Chronic inflammation has been closely linked to the pathophysiology of IBD, with dysregulation in immune signaling pathways potentially contributing to its development. Notably, PLAUR showed significant associations with both subtypes of IBD—UC and CD. Among these, PLAUR emerged as a key protein of interest; previous studies identified it as having the strongest effect size for depression scores [48], and in our study, it also contributed the most to IBD risk. PLAUR is involved in the plasminogen activation system and has been implicated in neuroinflammatory processes [49]. Furthermore, HIF-dependent NFATC1 activation has been shown to upregulate ITGA5 and PLAUR expression in the intestinal epithelium in IBD [50]. Similarly, Yang Cheng et al. provided functional validation showing that genetic or pharmacological inhibition of PLAUR or PLAU protected against intestinal epithelial barrier damage in cells and organoids, with PLAUR-deficient mice exhibiting reduced epithelial injury in DSS-induced colitis. These evidences suggest that elevated PLAUR may serve as a molecular link between social isolation, loneliness, and the onset of IBD.

### Limitations

The major strengths of this study include the integration of a large prospective cohort with multi-omics profiling and TSMR, enabling triangulation of observational, molecular, and causal evidence. This comprehensive approach provides mechanistic insights into how social isolation and loneliness may influence IBD risk through interconnected brain-immune-gut pathways. However, there are certain limitations in this study. First, although the assessment method of social isolation and loneliness has been used in previous research, future research should adopt validated and consistent social isolation and loneliness assessment instrument to increase comparability between studies. Second, the cohort study did not record changes in social isolation and loneliness during the follow-up period, which could lead to bias of the observed associations. Third, our study was conducted solely in the European population, which reduces the generalizability of the study results. Future research should include a more diverse population. Finally, as with most cohort studies, healthy volunteer bias may be present, potentially affecting the generalizability of our findings.

## Conclusion

Our findings demonstrate a significant association between social isolation, loneliness, and an increased risk of IBD. In particular, lower levels of DHA and elevated circulating PLAUR protein observed in socially isolated and lonely individuals were linked to higher IBD risk. Addressing social isolation and loneliness through targeted clinical psychosocial strategies merits increased focus as a potential means to reduce IBD risk. Furthermore, unraveling the biological mechanisms linking social relationships, immune and inflammatory functions, and gut health could pave the way for novel, precision-based preventive and therapeutic approaches.

## Supplementary information

Supplementary materials

## Data Availability

The data used in this study are available from the UK Biobank upon submission and approval of a data access application (https://www.ukbiobank.ac.uk). This research was conducted under UK Biobank application number 724597.

## Code availability

The analysis code used for data analysis is available from the corresponding author on reasonable request.
