# Supplementary material for: Lonely minds, inflamed guts: metabolic and circulating protein pathways linking social isolation and loneliness to inflammatory bowel disease
Source: Transl Psychiatry. 2026 May 22;16:365. doi: 10.1038/s41398-026-04116-0 (PMC13373236; doi:10.1038/s41398-026-04116-0)
Supplement: Supplementary file 1 — Supplementary materials [file 41398_2026_4116_MOESM1_ESM.docx]

**Supplementary Materials**

**Lonely Minds, Inflamed Guts: Metabolic and Circulating Protein Pathways Linking Social Isolation and Loneliness to Inflammatory Bowel Disease**

Jianhui Zhao^1,2,3*^, Jingyu Ye^1*^, Meng Zhang^1*^, Guirong Yu^1^, Haosen Ji^1^, Siyun Zhou^1^, Fangyuan Jiang^1^, Erxu Xue^2,4^, Kangning Li^1^, Ziqing Yu^5^, Hong Yang^5#^, Hao Wu^6#^, Xue Li^1, 7#^

^1^ Center of Clinical Big Data and Analytics of The Second Affiliated Hospital, School of Public Health, Zhejiang University School of Medicine, Hangzhou, Zhejiang, China.

^2^ Clinical and Translational Epidemiology Unit, Massachusetts General Hospital and Harvard Medical School, Boston, MA, USA.

^3^ Division of Gastroenterology, Massachusetts General Hospital and Harvard Medical School, Boston, MA, USA.

^4^ Nursing Department, Sir Run Run Shaw Hospital, Zhejiang University School of Medicine, Hangzhou, Zhejiang, China.

^5^ Department of Gastroenterology, Peking Union Medical College Hospital, Chinese Academy of Medical Sciences and Peking Union Medical College, Beijing, China

^6^ Department of Gastroenterology, National Clinical Research Center for Digestive Diseases, Changhai Hospital; National Key Laboratory of Immunity and Inflammation, Naval Medical University, Shanghai, China.

^7^ Zhejiang Key Laboratory of Intelligent Preventive Medicine, Hangzhou, Zhejiang, China

* These authors contributed equally.

**Correspondence to:** Xue Li (Address: 866 YuhangTang Rd, Xihu Dist., Hangzhou 310058, China; E-mail: [xueli157@zju.edu.cn](mailto:xueli157@zju.edu.cn)); Hao Wu (Address: 168 Changhai Road, Yangpu District, Shanghai, China; E-mail: [wuhao_lnly@163.com](mailto:wuhao_lnly@163.com)); Hong Yang (Address: Peking Union Medical College Hospital, No.1 Shuaifuyuan, Dongcheng District, Beijing, 100730; E-mail: [yangh@pumch.cn](mailto:yangh@pumch.cn)).

**Supplementary Methods**

**Definitions of social isolation and loneliness**

Social isolation was evaluated through responses to three questions on questionnaires: 1) “How often do you visit friends or family or have them visit you?” (1 point for the response of “once a month”, “once every few months”, “never or almost never”, or “no friends or family outside the household” and 0 point for the response of “once a week”, “2-4 times a week”, and “Almost daily”); 2) “Which of the following leisure or social activities do you engage in once a week or more often? You may select more than one of them: sports club or gym, pub or social club, religious group, adult education class, or other group activities” (1 point for the response of “none of above” and 0 point for the response of one of activities mentioned above); and 3) “Including yourself, how many people live in your household? Include those who usually live in the house such as students living away from home during term time, and partners in the armed forces or in professions such as pilots” (1 point for the response of “0” and 0 point for other numbers higher than 0). The social isolation score was calculated by summing up the points of three questions (ranging from 0 to 3). Participants were allocated into three groups according to the social isolation score: least isolated group (0 point), moderately isolated group (1 point), and most isolated group (2 or 3 points). Loneliness was assessed by two questions from UKB questionnaires: “Do you often feel lonely?” (1 point for the response of “yes” and 0 point for “no”) and “How often are you able to confide in someone close to you?” (1 point for the response of “never or almost never” and 0 point for the response of “once every few months”, “once a month”, “once a week”, “2-4 times a week”, or “almost daily”). The loneliness score was determined by adding up the scores from two questions, resulting in a range of 0–2 points. Individuals were allocated into two groups according to the loneliness score: the no-loneliness group (0 or 1 point) and the loneliness group (2 points). The assessment methods for loneliness and social isolation were consistent with published studies focusing on the topic of loneliness and social isolation ^1^.

**Detailed information on** **mendelian randomization**

Mendelian randomization (MR) leverages genetic variants as instrumental variables (IVs) that are randomly assigned at conception to investigate whether a lifetime exposure is causally associated with an outcome, thus are less susceptible to reverse causality and potential confounding than observational studies. Two Sample MR was conducted by integrating SNP associations for loneliness and social isolation with inflammatory bowel disease and subtypes in our study. IVs for both exposure s and outcome were derived from Genome-wide association study (GWAS) based on European ancestry. To be specific, feeling lonely, being willing to confide in others, not living alone, contact with friends or family, fewer leisure social activities, engaging in more sports clubs or gyms, pubs or social clubs, religious activities, adult education classes, and other group activities (GWAS IDs: ukb-b-8476/4982/5445/5379/5076/4000/4171/4667/1553/4077) were considered to be exposures in the MR analysis, which derived from the UKB GWAS consisting of 463,010 European ancestry participants. After significance screening (*P* < 5×10^-6^) and LD clumping (r^2^ < 0.01, window size = 10,000 kb), a total of 914 IVs for loneliness and social isolation were identified. IVs which are strongly significant in outcome GWAS (*P* < 5×10^-8^) were excluded from our analyses because of the possibility of violating the assumption of MR. To avoid potential pleiotropy, outcome GWAS was extracted from a nonoverlapping European ancestry GWAS study published by de Lang et al in 2017. (cases/controls for IBD: 25,042/34,915; UC: 12,366/33,609; CD: 12,194/28,072). All analyses were conducted using R (version 4.3.1) software and TwoSampleMR package. Nominal P-value < 0.05 was considered significant.

**Detailed information on the statistical analysis of mendelian randomization**

We employed the multiplicative random-effects inverse-variance weighted (MRE-IVW) method to conduct the main MR analysis, addressing potential heterogeneity. This approach integrated the Wald ratio estimates of individual SNPs to derive a single causal estimate for each risk factor, which was calculated by dividing the association between SNP and outcome by the association between SNP and exposure. Sensitivity analyses were performed using four MR methods: fixed-effects inverse-variance weighted (FE-IVW), MR-Egger, penalized weighted median, and weighted median (WM) approaches. Cochran's Q statistic was used to assess heterogeneity among the included IVs. A significance level of P-value < 0.05 indicated substantial heterogeneity. We also implemented MR-Radial method to removing outlier SNPs to avoid potential bias. To account for pleiotropy effects, we evaluated the intercepts of MR-Egger analysis and results of MR-PRESSO global test. Additionally, as part of sensitivity analysis, leave-one-out analysis was performed to ascertain whether a particular SNP drives main causal associations by systematically excluding SNPs from the model.

In reverse MR analysis, we evaluated reverse causality between loneliness and social isolation with IBD. 82 SNPs which overlapped in IVs of IBD and its subtypes were excluded for consideration of controlling for potential pleiotropy before analysis, and 182 SNPs were selected as final IVs (IBD:79; CD:58; UC:45). MRE-IVW method was still used for the main analysis. Heterogeneity test and Pleiotropy test were implemented to ensure the robustness of conclusions.

**Single-cell RNA sequencing analysis**

To investigate the expression patterns and potential functions of the target gene at the single-cell level, we obtained 70 single-cell RNA-seq samples from four GEO datasets (GSE21469573, GSE23199374, GSE26084275, and GSE15051676), comprising 14 UC, 18 CD, 9 UC-NIT, 19 CD-NIT, and 10 Normal samples. After standard preprocessing using the *Seurat* package, including cell filtering and doublet removal with DoubletFinder, we retained 230,075 high-quality cells. These cells were normalized, scaled, and integrated using the Harmony algorithm. Dimensionality reduction and clustering identified 20 subclusters. Cell types were annotated based on canonical markers and the *SingleR* package, resulting in seven major cell types: endothelial cells, fibroblasts, epithelial cells, myeloid cells, B cells, NK/T cells, and plasma cells.

To further explore cell-type-specific expression and function of the target gene, we identified subclusters with high expression of the gene and compared their average expression across tissue types using the Wilcoxon test. Based on marker genes from published literature and the CellMarker 2.0 database, subclusters with high target gene expression were annotated (resolution = 0.5), resulting in five distinct immune cell populations: macrophages (FCGR3A, CD14, CD68), neutrophils (FCGR3B, CSF3R), mast cells (TPSAB1, CPA3), dendritic cells (e.g., CLEC9A, XCR1, CD1C, FCER1A, LAMP3), and doublets (CD79A, CD79B, CD3D, CD3E). After removing potential doublets (n = 762), we compared gene expression between inflamed and non-inflamed tissues, and between inflamed and normal tissues within the remaining four immune cell populations in UC and CD, using the Wilcoxon test. Finally, to infer the functional role of the target gene, we performed Gene Ontology (GO) enrichment analysis within the macrophage subcluster. Cells were stratified into high and low expression groups based on the median expression of the target gene within UC and CD separately. Differentially expressed genes (DEGs) upregulated in the high-expression group were identified using the FindAllMarkers function (criteria: min.pct = 0.2, FDR-adjusted p < 0.05, log2FC > 1). Functional enrichment of DEGs was performed using the clusterProfiler package, with significant pathways filtered by FDR-adjusted p < 0.05. The top 10 pathways ranked by significance were visualized.


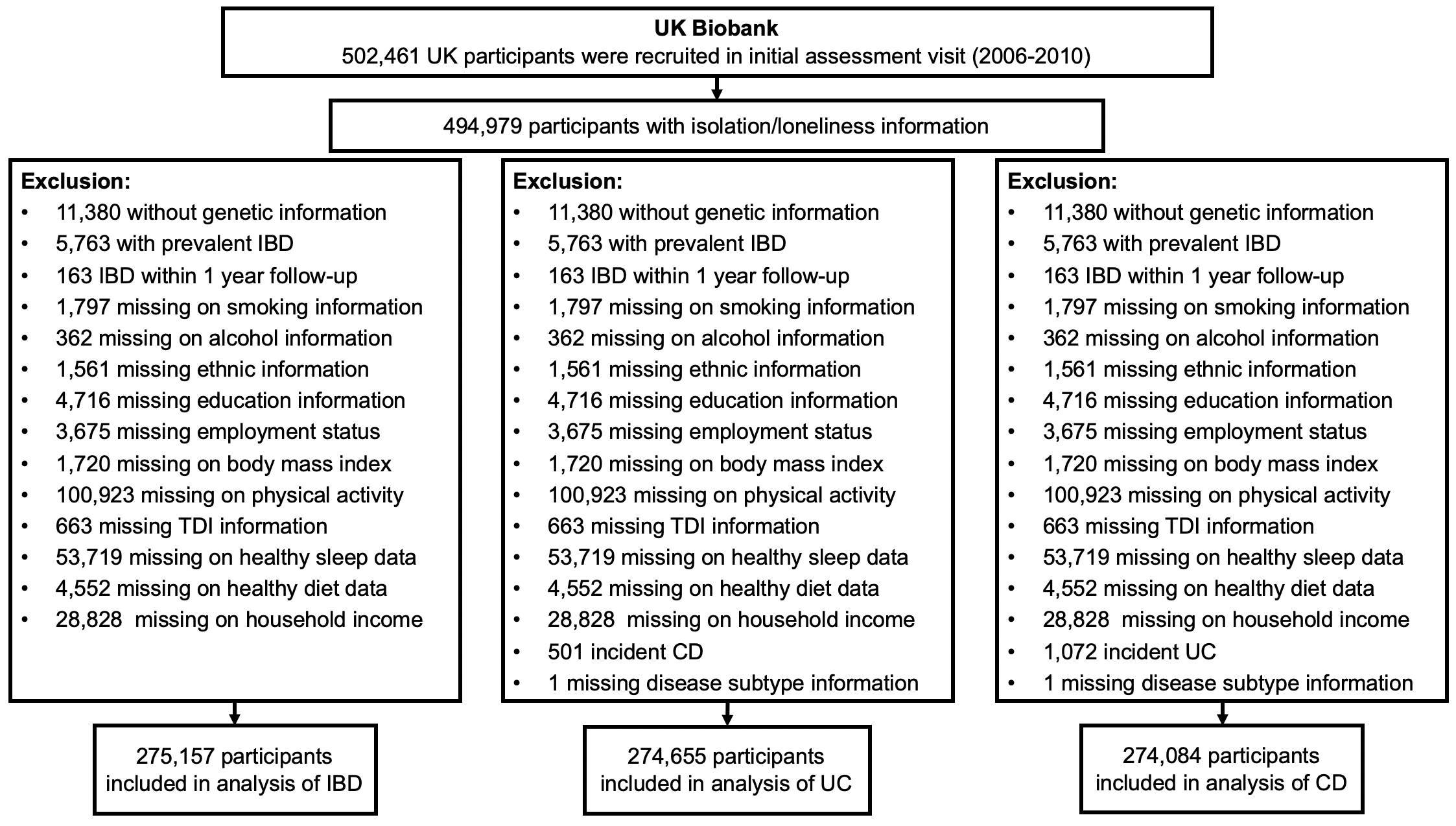


Supplementary Figure S1. Flow chart of the inclusion of the participants.

Abbreviation: CD, Crohn’s disease; IBD, inflammatory bowel disease; TDI, Townsend deprivation index; UC, ulcerative colitis.


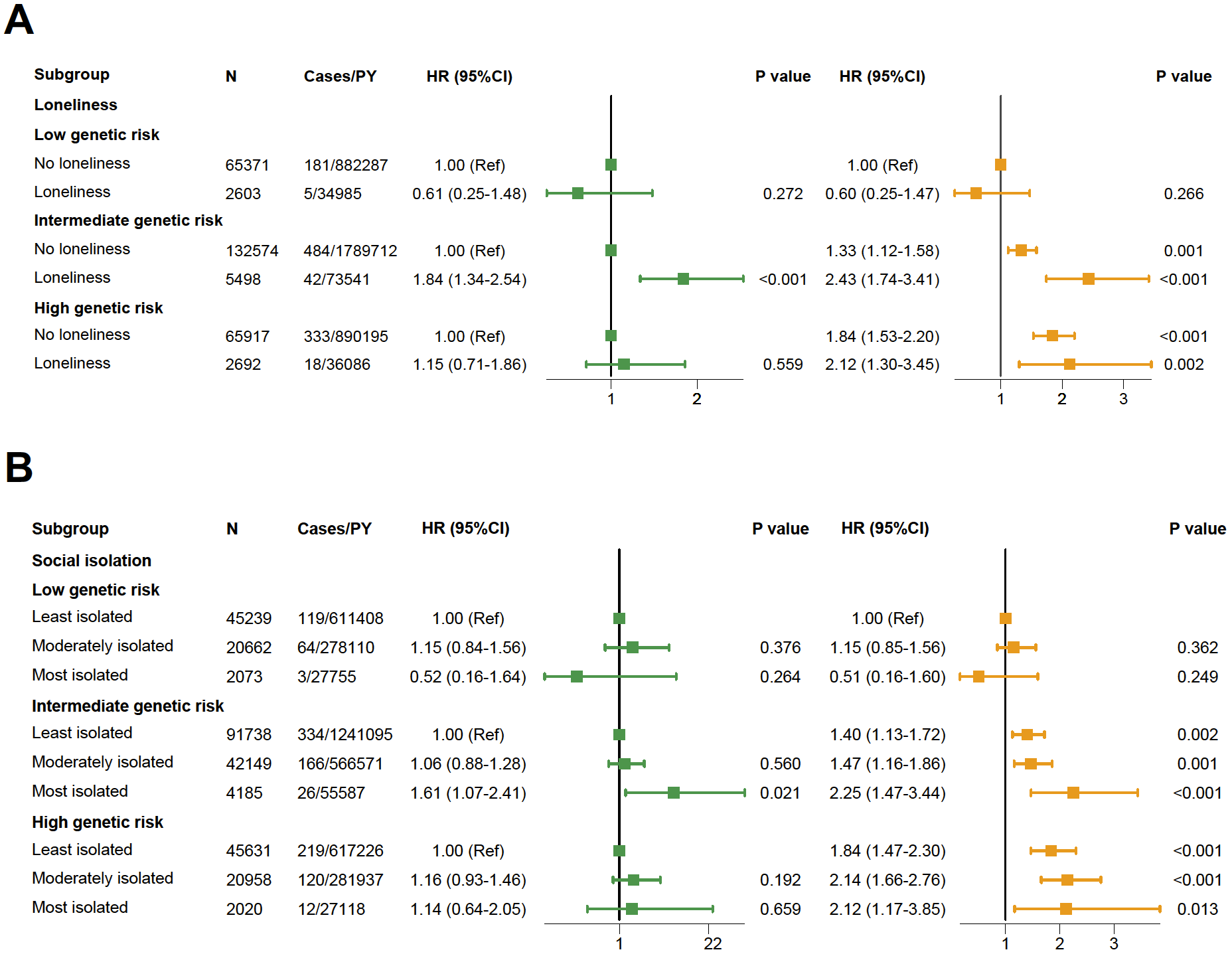


**Supplementary Figure S2. Separate and joint association of loneliness (A) and social isolation (B) with the long-term risk of UC across different levels of genetic susceptibility.** These analyses were conducted using Model 2, which adjusted for age, sex, ethnicity, BMI, household income, education level, employment status, smoking status, alcohol consumption, physical activity, healthy diet pattern, and healthy sleep pattern. Genetic risk categories were defined as low, intermediate, and high based on the quartiles of the PRS for UC, corresponding to quartile 1, quartiles 2-3, and quartile 4, respectively. Abbreviations: BMI, body mass index; PRS, polygenic risk score; UC, ulcerative colitis.


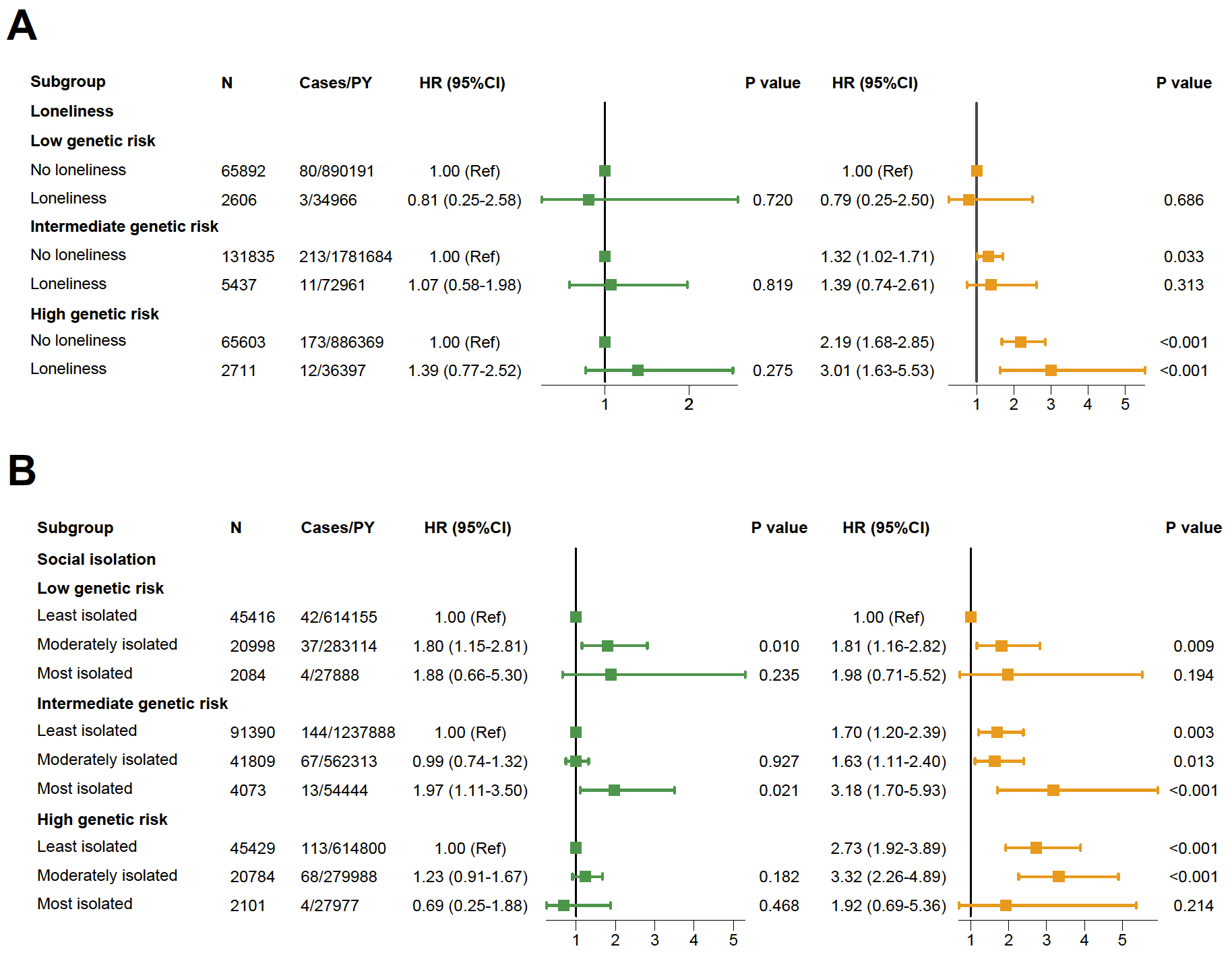


**Supplementary Figure S3. Separate and joint association of loneliness (A) and social isolation (B) with the risk of CD across different levels of genetic susceptibility.**

These analyses were conducted using Model 2, which adjusted for age, sex, ethnicity, BMI, household income, education level, employment status, smoking status, alcohol consumption, physical activity, healthy diet pattern, and healthy sleep pattern. Genetic risk categories were defined as low, intermediate, and high based on the quartiles of the PRS for CD, corresponding to quartile 1, quartiles 2-3, and quartile 4, respectively. Abbreviations: BMI, body mass index; CD, Crohn’s disease; PRS, polygenic risk score.

**
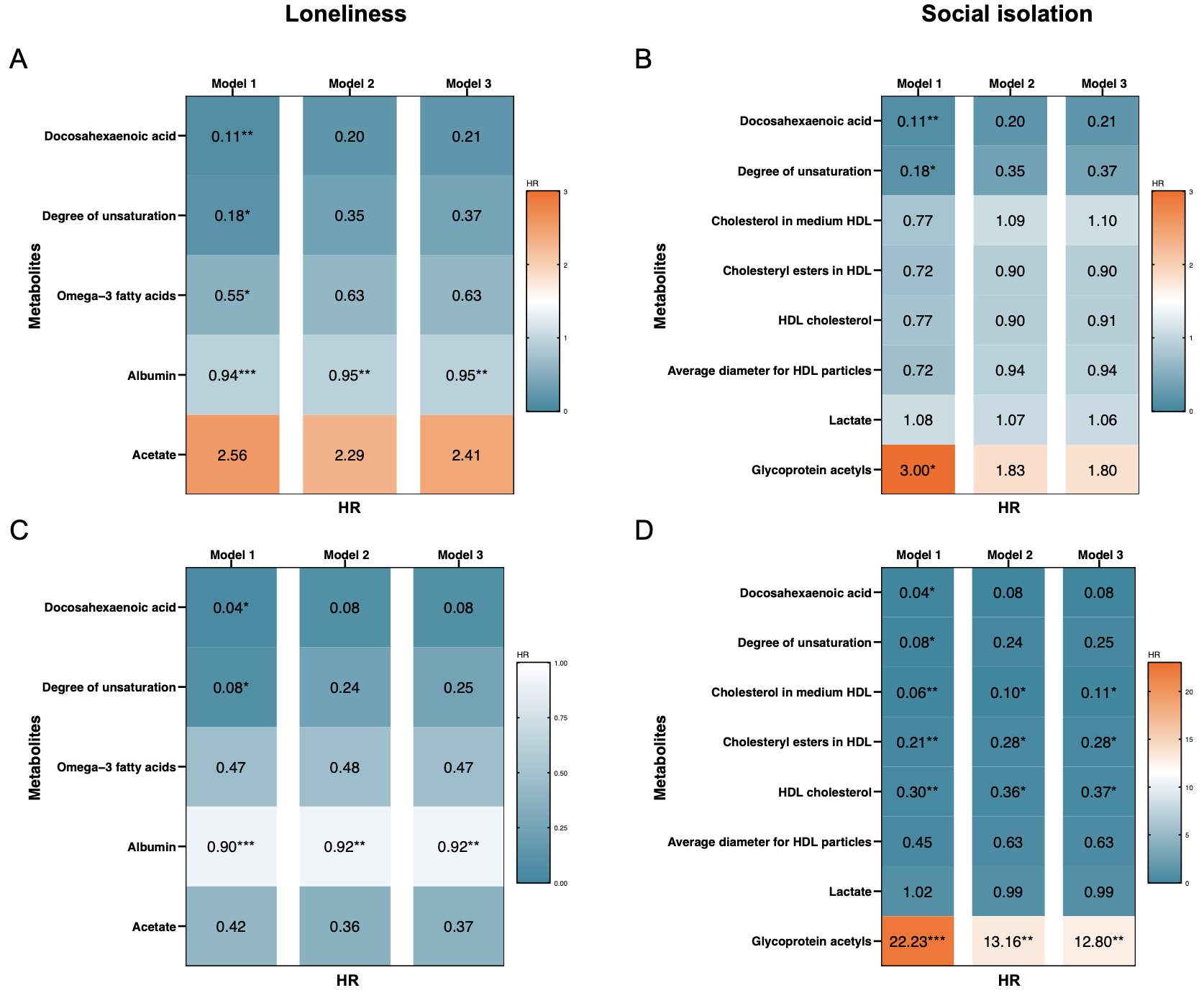
**

**Supplementary Figure S4. Associations between** **loneliness- and social isolation-related** **metabolites and the risk of UC (A and B) and CD (C and D).** The analysis was performed based on Model 3. Abbreviations: CD, Crohn’s disease; IBD, inflammatory bowel disease; UC, ulcerative colitis.

**Supplementary Table S1.** ICD codes assigned for different phenotypes of IBD, CD and UC.

| **Diagnoses** | **ICD-9** | **ICD-10** | **Self-report** | **First reported** |
| --- | --- | --- | --- | --- |
| **IBD** | "555", "5550", "5551", "5552", "5559", "556", "5560", "5561", "5562", "5563", "5564", "5565", "5566", "5568", "5569", | "K50", "K500", "K501", "K508", "K509", "K51", "K510", "K512", "K513", "K514", "K515", "K518", "K519" | 1461: inflammatory bowel disease;  1462: Crohn’s disease;  1463: ulcerative colitis |  |
| **CD** | "555", "5550", "5551", "5552", "5559" | "K50", "K500", "K501", "K508", "K509" | 1462 | "K50" |
| **UC** | "556", "5560", "5561", "5562", "5563", "5564", "5565", "5566", "5568", "5569" | "K51", "K510", "K512", "K513", "K514", "K515", "K518", "K519" | 1463 | "K51" |

Abbreviation: CD, Crohn’s disease; IBD, inflammatory bowel disease; ICD, International Classification of Diseases; UC, ulcerative colitis.

Supplementary Table S2. Definitions for covariates.

| **Covariates** | **Definition** | **Field code** |
| --- | --- | --- |
| Age | Age based on the date of birth and date of attending an initial assessment center and refers to the age of the participant on the day they attended an Initial Assessment Centre (variable handling: <65 or >=65 years old) | 21003 |
| Sex | A mixture of the sex the National Health Service had recorded for the participant and self-reported sex (categorical variable “Female”, “Male”). | 31 |
| Ethnicity | Self-reported: “What is your ethnic background”. We classified the variable into: White and Others (Mixed, Asian or Asian British, Black or Black British, Chinese, and other ethnic groups) because the number of non-white ethnic backgrounds was too small | 21000 |
| Household income | Self-reported: "What is the average total income before tax received by your HOUSEHOLD?". We classified the variable into high: ≥ £52,000, low: < £52 000 | 738 |
| Townsend deprivation index (TDI) | The higher, the more socioeconomic deprivation one was suffering. TDI was derived according to the unemployment rate, the percentage of overcrowded households, the percentage of people without cars, and the percentage of people without houses for each area in the UK, and baseline TDI was calculated immediately before the participant joined UK Biobank based on the preceding national census output areas. Each participant was assigned a score corresponding to the output area in which their postcode is located. We classified the variable into: Low deprivation/ Moderate deprivation/ High deprivation | 22189 |
| Current employment status | Self-reported: "Which of the following describes your current situation? (You can select more than one answer)". We classified the variable into current employed or not | 6142 |
| Education level | Self-reported: “Which of the following qualifications do you have” We classified the variable into College (College or University degree) and Below college (A levels/AS levels or equivalent, O levels/GCSEs or equivalent, CSEs or equivalent, NVQ or HND or HNC or equivalent, other professional qualifications eg: nursing, teaching, and none of the above) (classified into: College/university degree or Other qualifications) | 6138 |
| Smoking status | Self-reported: current/past smoking status of the participant. We classified the variable handlings into Current smoker or Not | 20116 |
| Drinking status | Self-reported: "About how often do you drink alcohol?" We classified the variable handlings into Current drinker or Not | 20117 |
| Physical activity | evaluated by metabolic equivalent task [MET], According to the median number of total exercise days, they were divided into two groups: high and low | 22033 |
| Charlson Comorbidity Index (CCI) | CCI is a highly cited and well-established tool for measuring comorbidity in clinical research. We calculated CCI as a variable reflecting objective heath status (range: 0–16). Mak JKL et al developed the calculation of CCI in the UK Biobank, which were constructed based on 17 comorbidities (myocardial infarction, congestive heart failure, peripheral vascular disease, cerebral vascular disease, dementia, pulmonary disease, connective tissue disorder, peptic ulcer, liver disease, diabetes, diabetes complications, paraplegia, renal disease, cancer, metastatic cancer, severe liver disease, and acquired immune deficiency | 30710 |
| Healthy diet pattern | The evaluation for the diet pattern was based on five dietary factors: vegetable intake of at least four tablespoons each day (median); fruits intake at least three pieces each day (median); fish intake at least twice each week (median); unprocessed red meat intake no more than twice each week (median); and processed meat intake no more than two each week (median). Each point was given for each favorable diet factor, with the total diet score ranging from 0 to 5, and a healthy diet pattern was defined as a healthy sleep score ≥ 4 points | 1289, 1299, 1309, 1319, 1329, 1339, 1369, 1379, 1389, 1349 |
| Healthy sleep pattern | The sleep pattern is evaluated by a healthy sleep score calculated from five factors: insomnia, sleep duration, chronotype, daytime sleepiness, and snoring. Healthy sleep factors were defined as “never/rarely or sometimes insomnia symptoms”, “sleep 7-8 h per day”, “no self-reported snoring”, and “no excessive daytime sleepiness” (never/rarely or sometimes).  Each sleep factor is coded 1 point if meeting the healthy criterion and 0 if not. The healthy sleep score ranged from 0-5, and a healthy sleep pattern was defined as a healthy sleep score ≥ 4 points | 1200, 1180, 1160, 1220, 1210 |
| Body mass index (BMI) | BMI value is constructed from height and weight measured during the initial assessment center visit. The relevant variable was measured by trained staff. We classified the variable into: <25 kg/m^2^, 25-29.9 kg /m^2^, ≥30 kg/m^2^ | 21001 |
| **Exposure** |  |  |
| Social isolation | - | 709/1031/6160 |
| Loneliness | - | 2020/2110 |

Abbreviation: ICD, International Classification of Diseases.

Supplementary Table S3. Genetic instrumental variables for inflammatory bowel diseases.

| **SNP** | **Chr** | **OA** | **EA** | **Beta** | **SE** | **EAF** | **P value** | **F-statistics** |
| --- | --- | --- | --- | --- | --- | --- | --- | --- |
| rs10746475 | 1 | A | T | -0.1308 | 0.0164 | 0.83 | 1.58E-15 | 63.61 |
| rs10800309 | 1 | A | G | -0.123 | 0.0133 | 0.3002 | 1.94E-20 | 85.52 |
| rs11209013 | 1 | A | G | 0.0773 | 0.0124 | 0.4751 | 4.46E-10 | 38.86 |
| rs112936798 | 1 | A | C | -0.1844 | 0.0332 | 0.9781 | 2.89E-08 | 30.85 |
| rs11581607 | 1 | A | G | 0.6578 | 0.0294 | 0.0616 | 4.59E-111 | 500.56 |
| rs12136659 | 1 | T | C | 0.087 | 0.0142 | 0.2515 | 1.02E-09 | 37.53 |
| rs1268339 | 1 | T | C | 0.0907 | 0.0163 | 0.2167 | 2.75E-08 | 30.96 |
| rs1336900 | 1 | A | G | 0.0848 | 0.0128 | 0.4095 | 2.98E-11 | 43.89 |
| rs2488398 | 1 | C | G | -0.0985 | 0.0149 | 0.1899 | 3.63E-11 | 43.7 |
| rs3024493 | 1 | A | C | -0.1911 | 0.0165 | 0.834 | 4.04E-31 | 134.13 |
| rs35730213 | 1 | C | G | 0.1346 | 0.014 | 0.2744 | 7.50E-22 | 92.43 |
| rs3820330 | 1 | A | C | 0.0892 | 0.014 | 0.6998 | 1.72E-10 | 40.59 |
| rs4276914 | 1 | A | G | -0.0783 | 0.0125 | 0.494 | 3.15E-10 | 39.23 |
| rs6674040 | 1 | T | G | 0.1129 | 0.0124 | 0.5268 | 6.31E-20 | 82.89 |
| rs7532133 | 1 | A | G | 0.0789 | 0.0134 | 0.3231 | 3.83E-09 | 34.67 |
| rs7543680 | 1 | A | G | 0.1001 | 0.0153 | 0.2187 | 5.51E-11 | 42.8 |
| rs11677002 | 2 | T | C | -0.0931 | 0.0126 | 0.4592 | 1.37E-13 | 54.59 |
| rs13422838 | 2 | T | C | -0.1143 | 0.0205 | 0.0954 | 2.56E-08 | 31.08 |
| rs1558619 | 2 | T | G | 0.0843 | 0.0123 | 0.493 | 8.90E-12 | 46.97 |
| rs3792111 | 2 | T | C | -0.1391 | 0.0124 | 0.5368 | 5.12E-29 | 125.83 |
| rs4676408 | 2 | A | G | -0.1011 | 0.013 | 0.4881 | 7.63E-15 | 60.48 |
| rs55946629 | 2 | A | C | -0.1298 | 0.018 | 0.1233 | 5.45E-13 | 52 |
| rs62180107 | 2 | C | G | 0.0797 | 0.0132 | 0.3688 | 1.55E-09 | 36.45 |
| rs62183956 | 2 | T | C | 0.078 | 0.0125 | 0.5 | 4.49E-10 | 38.93 |
| rs6740847 | 2 | A | G | -0.0924 | 0.0125 | 0.4553 | 1.22E-13 | 54.64 |
| rs72852162 | 2 | A | C | -0.1129 | 0.0202 | 0.8897 | 2.30E-08 | 31.24 |
| rs7608697 | 2 | A | C | 0.1395 | 0.0126 | 0.6372 | 1.67E-28 | 122.57 |
| rs76286777 | 2 | T | C | 0.0996 | 0.0151 | 0.2048 | 4.66E-11 | 43.5 |
| rs1131095 | 3 | T | C | 0.1635 | 0.0131 | 0.3241 | 1.22E-35 | 155.76 |
| rs2593855 | 3 | T | C | 0.0832 | 0.014 | 0.675 | 2.54E-09 | 35.31 |
| rs503734 | 3 | A | G | -0.0692 | 0.0124 | 0.5139 | 2.67E-08 | 31.14 |
| rs56116661 | 3 | T | C | 0.1 | 0.0163 | 0.8062 | 9.27E-10 | 37.63 |
| rs77272631 | 3 | C | G | -0.2293 | 0.0417 | 0.0408 | 3.72E-08 | 30.23 |
| rs11734570 | 4 | A | G | -0.0694 | 0.0127 | 0.4473 | 4.80E-08 | 29.86 |
| rs62324212 | 4 | A | C | -0.0886 | 0.0127 | 0.4642 | 2.67E-12 | 48.67 |
| rs10041497 | 5 | T | C | 0.0819 | 0.0129 | 0.6203 | 1.95E-10 | 40.3 |
| rs11739135 | 5 | C | G | -0.1366 | 0.0125 | 0.3738 | 1.10E-27 | 119.41 |
| rs1445004 | 5 | T | C | -0.1689 | 0.0127 | 0.5895 | 3.48E-40 | 176.85 |
| rs254837 | 5 | C | G | -0.1222 | 0.0205 | 0.8787 | 2.54E-09 | 35.53 |
| rs341295 | 5 | T | C | -0.0702 | 0.0124 | 0.4652 | 1.45E-08 | 32.05 |
| rs4957256 | 5 | T | C | 0.1179 | 0.0155 | 0.7674 | 3.37E-14 | 57.85 |
| rs56235845 | 5 | T | G | 0.0877 | 0.0138 | 0.341 | 1.77E-10 | 40.38 |
| rs6579807 | 5 | T | C | -0.125 | 0.0189 | 0.84 | 4.01E-11 | 43.74 |
| rs6873866 | 5 | T | C | -0.0919 | 0.0128 | 0.5954 | 6.15E-13 | 51.54 |
| rs755374 | 5 | T | C | -0.1767 | 0.0134 | 0.327 | 1.59E-39 | 173.87 |
| rs11152949 | 6 | A | G | 0.1019 | 0.0133 | 0.6968 | 1.56E-14 | 58.7 |
| rs1267496 | 6 | C | G | -0.1053 | 0.0159 | 0.2306 | 3.39E-11 | 43.86 |
| rs143210366 | 6 | T | G | 0.2836 | 0.036 | 0.0398 | 3.14E-15 | 62.05 |
| rs145568234 | 6 | T | G | 0.86 | 0.0476 | 0.0139 | 4.73E-73 | 326.4 |
| rs212402 | 6 | A | G | 0.0743 | 0.013 | 0.3569 | 1.06E-08 | 32.66 |
| rs34140409 | 6 | T | C | 0.1583 | 0.0237 | 0.8728 | 2.28E-11 | 44.61 |
| rs35171809 | 6 | A | G | 0.1088 | 0.0123 | 0.5527 | 1.16E-18 | 78.24 |
| rs4712528 | 6 | C | G | -0.1043 | 0.0152 | 0.7932 | 7.14E-12 | 47.08 |
| rs62408218 | 6 | T | C | 0.0818 | 0.0129 | 0.6243 | 2.40E-10 | 40.21 |
| rs6457681 | 6 | T | G | 0.1687 | 0.0153 | 0.7803 | 3.75E-28 | 121.57 |
| rs6933404 | 6 | T | C | 0.0863 | 0.0149 | 0.169 | 6.64E-09 | 33.54 |
| rs10953551 | 7 | A | G | -0.1033 | 0.0127 | 0.3966 | 4.94E-16 | 66.15 |
| rs11768365 | 7 | A | G | -0.0837 | 0.0152 | 0.8032 | 3.88E-08 | 30.32 |
| rs1456896 | 7 | T | C | -0.0879 | 0.0133 | 0.6869 | 4.50E-11 | 43.68 |
| rs149169037 | 7 | A | G | 0.1338 | 0.0242 | 0.0755 | 3.26E-08 | 30.57 |
| rs243505 | 7 | A | G | -0.0805 | 0.0128 | 0.4185 | 3.04E-10 | 39.55 |
| rs2529269 | 7 | T | C | 0.1042 | 0.0163 | 0.7167 | 1.70E-10 | 40.86 |
| rs62482552 | 7 | A | G | 0.0737 | 0.0131 | 0.5726 | 1.97E-08 | 31.65 |
| rs4380956 | 8 | A | G | -0.0907 | 0.0127 | 0.6302 | 1.12E-12 | 51 |
| rs78771661 | 8 | T | C | 0.3848 | 0.0669 | 0.9751 | 8.95E-09 | 33.08 |
| rs938650 | 8 | A | G | 0.1074 | 0.0189 | 0.1044 | 1.41E-08 | 32.29 |
| rs10114470 | 9 | T | C | 0.1475 | 0.0137 | 0.675 | 4.10E-27 | 115.91 |
| rs1887428 | 9 | C | G | 0.1643 | 0.0131 | 0.6282 | 2.46E-36 | 157.29 |
| rs3829110 | 9 | A | G | 0.1574 | 0.0125 | 0.6064 | 3.52E-36 | 158.55 |
| rs10761659 | 10 | A | G | 0.1585 | 0.0126 | 0.497 | 2.30E-36 | 158.23 |
| rs10826797 | 10 | T | G | 0.099 | 0.0136 | 0.6899 | 3.99E-13 | 52.99 |
| rs111456533 | 10 | A | G | 0.1031 | 0.017 | 0.167 | 1.18E-09 | 36.78 |
| rs11195128 | 10 | T | C | -0.0792 | 0.0133 | 0.6799 | 2.74E-09 | 35.46 |
| rs1250573 | 10 | A | G | 0.098 | 0.0138 | 0.2863 | 1.11E-12 | 50.43 |
| rs2384352 | 10 | A | G | 0.0951 | 0.0131 | 0.6581 | 3.12E-13 | 52.7 |
| rs6584282 | 10 | A | G | -0.152 | 0.0124 | 0.493 | 1.19E-34 | 150.25 |
| rs7918084 | 10 | T | C | -0.071 | 0.0125 | 0.4602 | 1.38E-08 | 32.26 |
| rs11221335 | 11 | T | C | 0.0827 | 0.0148 | 0.2445 | 2.44E-08 | 31.22 |
| rs11236797 | 11 | A | C | -0.1488 | 0.0125 | 0.4503 | 7.19E-33 | 141.69 |
| rs11066188 | 12 | A | G | -0.0874 | 0.013 | 0.4135 | 1.76E-11 | 45.2 |
| rs117981694 | 12 | A | G | -0.3452 | 0.0411 | 0.0229 | 4.53E-17 | 70.54 |
| rs12825700 | 12 | A | G | -0.1324 | 0.0127 | 0.3598 | 1.28E-25 | 108.68 |
| rs140933577 | 13 | T | C | -0.1857 | 0.0305 | 0.0368 | 1.13E-09 | 37.07 |
| rs3897234 | 13 | T | C | 0.0971 | 0.0145 | 0.7664 | 1.90E-11 | 44.84 |
| rs194746 | 14 | T | C | -0.0833 | 0.0124 | 0.5288 | 1.84E-11 | 45.12 |
| rs3850378 | 14 | T | C | 0.1536 | 0.0207 | 0.1054 | 1.10E-13 | 55.06 |
| rs1864239 | 15 | A | G | 1.3366 | 0.1782 | 0.0189 | 6.27E-14 | 56.25 |
| rs56062135 | 15 | T | C | -0.1382 | 0.0145 | 0.7853 | 1.37E-21 | 90.83 |
| rs11548656 | 16 | A | G | -0.2374 | 0.0362 | 0.9602 | 5.18E-11 | 43 |
| rs16940202 | 16 | T | C | 0.113 | 0.0169 | 0.1889 | 2.51E-11 | 44.7 |
| rs2301127 | 16 | A | G | -0.0783 | 0.0126 | 0.4791 | 4.96E-10 | 38.61 |
| rs28374519 | 16 | A | G | 0.1105 | 0.0137 | 0.4612 | 6.55E-16 | 65.05 |
| rs7190426 | 16 | A | C | -0.0872 | 0.0155 | 0.8121 | 2.06E-08 | 31.65 |
| rs749910 | 16 | A | G | -0.1961 | 0.0138 | 0.2256 | 7.83E-46 | 201.91 |
| rs8056255 | 16 | A | T | -0.2765 | 0.0327 | 0.0368 | 2.99E-17 | 71.49 |
| rs9934775 | 16 | T | C | 0.1116 | 0.0172 | 0.8419 | 8.77E-11 | 42.1 |
| rs12936409 | 17 | T | C | -0.1406 | 0.0124 | 0.5278 | 7.73E-30 | 128.56 |
| rs2857656 | 17 | C | G | 0.0995 | 0.014 | 0.3161 | 1.07E-12 | 50.51 |
| rs4072601 | 17 | A | G | 0.1264 | 0.0177 | 0.8738 | 8.43E-13 | 50.99 |
| rs744166 | 17 | A | G | -0.1109 | 0.0126 | 0.4145 | 1.34E-18 | 77.46 |
| rs1319951 | 18 | C | G | -0.0851 | 0.0147 | 0.2475 | 7.50E-09 | 33.51 |
| rs80262450 | 18 | A | G | -0.1581 | 0.019 | 0.0895 | 1.04E-16 | 69.23 |
| rs11669299 | 19 | T | C | 0.1107 | 0.0157 | 0.828 | 1.84E-12 | 49.71 |
| rs4807569 | 19 | A | C | 0.1281 | 0.0152 | 0.8012 | 4.24E-17 | 71.02 |
| rs62126610 | 19 | A | G | 0.1407 | 0.0166 | 0.826 | 2.60E-17 | 71.84 |
| rs7256518 | 19 | A | G | 0.1665 | 0.0276 | 0.9503 | 1.63E-09 | 36.39 |
| rs154873 | 20 | A | G | 0.0813 | 0.0132 | 0.6481 | 7.38E-10 | 37.93 |
| rs4256018 | 20 | T | G | 0.0786 | 0.0138 | 0.2783 | 1.23E-08 | 32.44 |
| rs6017342 | 20 | A | C | 0.1156 | 0.0135 | 0.4801 | 1.07E-17 | 73.32 |
| rs6062496 | 20 | A | G | -0.137 | 0.0129 | 0.5994 | 2.83E-26 | 112.78 |
| rs6063502 | 20 | A | G | -0.0734 | 0.0134 | 0.5984 | 4.55E-08 | 30 |
| rs1297264 | 21 | A | G | -0.1462 | 0.0126 | 0.5616 | 3.98E-31 | 134.62 |
| rs2836881 | 21 | T | G | 0.1643 | 0.0146 | 0.7425 | 1.96E-29 | 126.63 |
| rs2838517 | 21 | T | C | -0.128 | 0.0125 | 0.6163 | 1.84E-24 | 104.85 |
| rs2413583 | 22 | T | C | 0.1732 | 0.0171 | 0.835 | 4.60E-24 | 102.58 |
| rs5754100 | 22 | T | C | 0.1293 | 0.016 | 0.1839 | 7.14E-16 | 65.3 |
| rs5763793 | 22 | T | G | -0.0734 | 0.013 | 0.6372 | 1.47E-08 | 31.88 |

Abbreviations: Beta, effect estimate; Chr, chromosome; EA, effect allele; EAF, effect allele frequency; OA, other allele; SE, standard error; SNP, single-nucleotide polymorphism.

Supplementary Table S4. Genetic instrumental variables for Crohn’s disease.

| **SNP** | **Chr** | **OA** | **EA** | **Beta** | **SE** | **P value** | **EAF** | **F-statistics** |
| --- | --- | --- | --- | --- | --- | --- | --- | --- |
| rs114802258 | 1 | T | C | 0.2245 | 0.0384 | 5.11E-09 | 9.35E-01 | 34.17 |
| rs12131079 | 1 | T | C | 0.1088 | 0.0174 | 3.99E-10 | 3.22E-01 | 39.09 |
| rs2476601 | 1 | A | G | 0.2312 | 0.0286 | 6.44E-16 | 9.44E-02 | 65.34 |
| rs3122605 | 1 | A | G | 0.1748 | 0.0227 | 1.24E-14 | 8.49E-01 | 59.29 |
| rs35730213 | 1 | C | G | 0.1166 | 0.0181 | 1.17E-10 | 2.74E-01 | 41.49 |
| rs4316387 | 1 | T | C | -0.1292 | 0.0189 | 7.75E-12 | 8.04E-01 | 46.72 |
| rs6704109 | 1 | T | C | -0.1748 | 0.0181 | 5.1E-22 | 7.66E-01 | 93.25 |
| rs7517847 | 1 | T | G | -0.3447 | 0.0165 | 5.84E-97 | 4.21E-01 | 436.36 |
| rs11677002 | 2 | T | C | -0.1124 | 0.0163 | 4.57E-12 | 4.59E-01 | 47.54 |
| rs11683692 | 2 | T | C | -0.2144 | 0.038 | 1.75E-08 | 5.47E-02 | 31.83 |
| rs1583792 | 2 | T | C | 0.0882 | 0.016 | 3.26E-08 | 4.49E-01 | 30.38 |
| rs2110735 | 2 | A | G | -0.1372 | 0.0185 | 1.2E-13 | 7.79E-01 | 54.99 |
| rs34004493 | 2 | A | G | 0.1258 | 0.0179 | 2E-12 | 7.54E-01 | 49.38 |
| rs3816234 | 2 | A | G | -0.2704 | 0.0162 | 1.51E-62 | 4.57E-01 | 278.56 |
| rs4343432 | 2 | A | G | 0.1123 | 0.0162 | 3.5E-12 | 5.53E-01 | 48.05 |
| rs55946629 | 2 | A | C | -0.1755 | 0.0231 | 2.85E-14 | 1.23E-01 | 57.71 |
| rs6740847 | 2 | A | G | -0.104 | 0.0161 | 9.72E-11 | 4.55E-01 | 41.72 |
| rs7608697 | 2 | A | C | 0.1229 | 0.0163 | 4.03E-14 | 6.37E-01 | 56.84 |
| rs2581828 | 3 | C | G | -0.0941 | 0.0162 | 6.46E-09 | 3.90E-01 | 33.73 |
| rs56116661 | 3 | T | C | 0.1312 | 0.0212 | 5.67E-10 | 8.06E-01 | 38.29 |
| rs6808936 | 3 | A | G | 0.0904 | 0.0161 | 1.93E-08 | 5.77E-01 | 31.52 |
| rs9836291 | 3 | A | G | -0.1722 | 0.017 | 3.77E-24 | 3.24E-01 | 102.59 |
| rs13107325 | 4 | T | C | -0.2006 | 0.0284 | 1.67E-12 | 7.95E-02 | 49.88 |
| rs62324212 | 4 | A | C | -0.106 | 0.0163 | 8.02E-11 | 4.64E-01 | 42.28 |
| rs73243877 | 4 | A | G | 0.1164 | 0.0212 | 4.12E-08 | 8.20E-01 | 30.14 |
| rs112856973 | 5 | T | C | -0.1612 | 0.0243 | 3.61E-11 | 1.31E-01 | 44 |
| rs181826 | 5 | A | C | -0.1162 | 0.0167 | 3.25E-12 | 3.80E-01 | 48.41 |
| rs2188962 | 5 | T | C | -0.2004 | 0.016 | 5.59E-36 | 6.13E-01 | 156.85 |
| rs6451494 | 5 | T | C | 0.2605 | 0.0166 | 8.26E-56 | 5.90E-01 | 246.22 |
| rs6579807 | 5 | T | C | -0.1993 | 0.0244 | 3.44E-16 | 8.40E-01 | 66.71 |
| rs6873866 | 5 | T | C | -0.1314 | 0.0164 | 1.35E-15 | 5.95E-01 | 64.18 |
| rs755374 | 5 | T | C | -0.1969 | 0.0174 | 1.38E-29 | 3.27E-01 | 128.03 |
| rs1012636 | 6 | T | G | -0.1291 | 0.0198 | 7.01E-11 | 7.94E-01 | 42.51 |
| rs111281598 | 6 | T | C | 0.2745 | 0.0316 | 4.17E-18 | 5.86E-02 | 75.45 |
| rs1321859 | 6 | T | C | 0.1049 | 0.0172 | 1.18E-09 | 3.63E-01 | 37.19 |
| rs145568234 | 6 | T | G | 0.8602 | 0.0633 | 4.31E-42 | 1.39E-02 | 184.64 |
| rs212408 | 6 | T | G | 0.1136 | 0.0167 | 9.12E-12 | 6.40E-01 | 46.26 |
| rs35171809 | 6 | A | G | 0.1566 | 0.0159 | 9.07E-23 | 5.53E-01 | 96.99 |
| rs552183536 | 6 | T | C | 0.163 | 0.0273 | 2.39E-09 | 1.13E-01 | 35.64 |
| rs73516754 | 6 | A | C | 0.1423 | 0.0169 | 4.04E-17 | 6.85E-01 | 70.89 |
| rs7753014 | 6 | C | G | -0.0989 | 0.0163 | 1.39E-09 | 5.20E-01 | 36.81 |
| rs9482770 | 6 | T | C | 0.0987 | 0.0162 | 1.01E-09 | 4.11E-01 | 37.11 |
| rs9501641 | 6 | T | C | -0.3027 | 0.0432 | 2.57E-12 | 9.58E-01 | 49.09 |
| rs9656588 | 7 | T | C | 0.1183 | 0.0173 | 8.73E-12 | 6.85E-01 | 46.75 |
| rs4380956 | 8 | A | G | -0.132 | 0.0165 | 1.15E-15 | 6.30E-01 | 63.99 |
| rs79832570 | 8 | T | C | 0.2234 | 0.0344 | 8.9E-11 | 9.34E-02 | 42.17 |
| rs938650 | 8 | A | G | 0.1747 | 0.0247 | 1.65E-12 | 1.04E-01 | 50.02 |
| rs10114470 | 9 | T | C | 0.1687 | 0.0177 | 1.76E-21 | 6.75E-01 | 90.83 |
| rs1887428 | 9 | C | G | 0.166 | 0.0169 | 8.54E-23 | 6.28E-01 | 96.47 |
| rs4077515 | 9 | T | C | -0.1848 | 0.0162 | 3.15E-30 | 3.97E-01 | 130.11 |
| rs10822050 | 10 | T | C | 0.1827 | 0.0162 | 2.35E-29 | 3.77E-01 | 127.17 |
| rs10884966 | 10 | A | G | -0.1131 | 0.0171 | 4.13E-11 | 3.20E-01 | 43.74 |
| rs1148246 | 10 | T | C | 0.1323 | 0.0167 | 2.09E-15 | 3.61E-01 | 62.75 |
| rs1250573 | 10 | A | G | 0.1522 | 0.0179 | 1.92E-17 | 2.86E-01 | 72.29 |
| rs2002695 | 10 | A | G | -0.1293 | 0.0189 | 8.31E-12 | 2.53E-01 | 46.8 |
| rs2675670 | 10 | C | G | -0.1074 | 0.0161 | 2.9E-11 | 5.87E-01 | 44.49 |
| rs61839660 | 10 | T | C | -0.1468 | 0.0261 | 1.98E-08 | 9.30E-01 | 31.63 |
| rs6584282 | 10 | A | G | -0.1658 | 0.016 | 3.44E-25 | 4.93E-01 | 107.36 |
| rs11236797 | 11 | A | C | -0.176 | 0.0161 | 8.51E-28 | 4.50E-01 | 119.48 |
| rs28999107 | 12 | T | G | -0.1083 | 0.0178 | 1.06E-09 | 5.63E-01 | 37.01 |
| rs34635748 | 12 | T | C | -0.4794 | 0.0504 | 1.95E-21 | 9.77E-01 | 90.46 |
| rs77566919 | 12 | A | G | 0.1089 | 0.0185 | 4.13E-09 | 6.88E-01 | 34.65 |
| rs1373904 | 13 | A | G | 0.141 | 0.0189 | 9.11E-14 | 2.26E-01 | 55.65 |
| rs194746 | 14 | T | C | -0.0975 | 0.0161 | 1.24E-09 | 5.29E-01 | 36.67 |
| rs3850378 | 14 | T | C | 0.199 | 0.0267 | 8.31E-14 | 1.05E-01 | 55.54 |
| rs72743461 | 15 | A | C | -0.1684 | 0.0187 | 2.26E-19 | 2.15E-01 | 81.08 |
| rs2021511 | 16 | T | C | 0.1082 | 0.0182 | 2.63E-09 | 2.96E-01 | 35.34 |
| rs2076756 | 16 | A | G | 0.385 | 0.0174 | 1.8E-108 | 7.74E-01 | 489.5 |
| rs42861 | 16 | A | G | 0.1243 | 0.0167 | 8.87E-14 | 3.95E-01 | 55.39 |
| rs59926756 | 16 | A | G | -0.1062 | 0.0176 | 1.74E-09 | 2.81E-01 | 36.4 |
| rs7195228 | 16 | C | G | -0.1327 | 0.0209 | 2.09E-10 | 8.12E-01 | 40.31 |
| rs72798422 | 16 | T | C | 0.5495 | 0.0382 | 6.05E-47 | 3.78E-02 | 206.89 |
| rs12936409 | 17 | T | C | -0.1426 | 0.016 | 4.31E-19 | 5.28E-01 | 79.42 |
| rs2857656 | 17 | C | G | 0.1576 | 0.0182 | 4.22E-18 | 3.16E-01 | 74.97 |
| rs2948542 | 17 | A | G | 0.1016 | 0.0163 | 5.15E-10 | 6.17E-01 | 38.85 |
| rs744166 | 17 | A | G | -0.1142 | 0.0162 | 1.8E-12 | 4.15E-01 | 49.69 |
| rs80262450 | 18 | A | G | -0.2268 | 0.0244 | 1.34E-20 | 8.95E-02 | 86.38 |
| rs144309607 | 19 | T | C | 0.3712 | 0.047 | 2.69E-15 | 9.69E-01 | 62.37 |
| rs4807570 | 19 | A | G | -0.1811 | 0.0193 | 6.03E-21 | 2.00E-01 | 88.03 |
| rs492602 | 19 | A | G | 0.1084 | 0.0162 | 2.33E-11 | 4.41E-01 | 44.77 |
| rs62126620 | 19 | A | G | -0.144 | 0.0201 | 8.61E-13 | 2.02E-01 | 51.32 |
| rs3761158 | 20 | A | G | 0.1098 | 0.0165 | 2.65E-11 | 4.16E-01 | 44.28 |
| rs6062496 | 20 | A | G | -0.1223 | 0.0167 | 2.62E-13 | 5.99E-01 | 53.62 |
| rs1297264 | 21 | A | G | -0.1769 | 0.0163 | 1.59E-27 | 5.62E-01 | 117.76 |
| rs2284553 | 21 | A | G | 0.1277 | 0.0165 | 1.14E-14 | 3.81E-01 | 59.89 |
| rs2838517 | 21 | T | C | -0.1456 | 0.0162 | 2.03E-19 | 6.16E-01 | 80.76 |
| rs2143178 | 22 | T | C | -0.2087 | 0.0223 | 6.84E-21 | 1.67E-01 | 87.57 |
| rs5754100 | 22 | T | C | 0.1687 | 0.0206 | 3.02E-16 | 1.84E-01 | 67.05 |

Abbreviations: Beta, effect estimate; Chr, chromosome; EA, effect allele; EAF, effect allele frequency; OA, other allele; SE, standard error; SNP, single-nucleotide polymorphism.

Supplementary Table S5. Genetic instrumental variables for ulcerative colitis.

| **SNP** | **Chr** | **OA** | **EA** | **Beta** | **SE** | **P value** | **EAF** | **F-statistics** |
| --- | --- | --- | --- | --- | --- | --- | --- | --- |
| rs10737481 | 1 | T | G | 0.2173 | 0.0159 | 2.56E-42 | 5.50E-01 | 186.75 |
| rs10917545 | 1 | A | G | 0.2233 | 0.0252 | 7.03E-19 | 8.89E-01 | 78.51 |
| rs11209026 | 1 | A | G | 0.483 | 0.0358 | 1.995E-41 | 6.16E-02 | 181.99 |
| rs2816954 | 1 | A | T | -0.1375 | 0.0229 | 1.798E-09 | 1.61E-01 | 36.05 |
| rs3024493 | 1 | A | C | -0.21 | 0.0209 | 7.458E-24 | 8.34E-01 | 100.94 |
| rs6658353 | 1 | C | G | 0.1569 | 0.016 | 1.168E-22 | 5.12E-01 | 96.15 |
| rs7523335 | 1 | A | G | 0.1389 | 0.021 | 3.421E-11 | 1.69E-01 | 43.74 |
| rs7544646 | 1 | C | G | -0.1168 | 0.016 | 2.532E-13 | 4.69E-01 | 53.28 |
| rs7554511 | 1 | A | C | 0.1448 | 0.0178 | 4.267E-16 | 2.73E-01 | 66.16 |
| rs79051659 | 1 | A | G | -0.1605 | 0.0264 | 1.295E-09 | 1.04E-01 | 36.95 |
| rs1811711 | 2 | C | G | -0.1299 | 0.0223 | 6.086E-09 | 1.80E-01 | 33.93 |
| rs4676408 | 2 | A | G | -0.1433 | 0.0167 | 1.19E-17 | 4.88E-01 | 73.62 |
| rs55905347 | 2 | A | G | -0.1054 | 0.0166 | 2.093E-10 | 3.67E-01 | 40.31 |
| rs62180181 | 2 | T | C | -0.1226 | 0.0171 | 8.084E-13 | 7.23E-01 | 51.39 |
| rs7608697 | 2 | A | C | 0.1597 | 0.0161 | 3.031E-23 | 6.37E-01 | 98.38 |
| rs1131095 | 3 | T | C | 0.1593 | 0.0168 | 2.177E-21 | 3.24E-01 | 89.9 |
| rs17656349 | 5 | T | C | -0.09 | 0.0159 | 1.54E-08 | 4.35E-01 | 32.03 |
| rs17715902 | 5 | A | G | -0.0974 | 0.0166 | 4.618E-09 | 3.42E-01 | 34.42 |
| rs67111717 | 5 | A | G | 0.0944 | 0.0171 | 3.269E-08 | 6.52E-01 | 30.47 |
| rs6889364 | 5 | A | G | -0.1318 | 0.0228 | 7.869E-09 | 1.35E-01 | 33.41 |
| rs72704802 | 5 | T | C | 0.1223 | 0.0206 | 2.894E-09 | 8.24E-01 | 35.24 |
| rs755374 | 5 | T | C | -0.1714 | 0.0171 | 9.73E-24 | 3.27E-01 | 100.45 |
| rs113986290 | 6 | T | C | 0.3066 | 0.0531 | 7.593E-09 | 9.72E-01 | 33.33 |
| rs13200059 | 6 | A | G | -0.2944 | 0.0436 | 1.484E-11 | 3.48E-02 | 45.59 |
| rs28383224 | 6 | A | G | -0.1468 | 0.0165 | 4.649E-19 | 3.91E-01 | 79.14 |
| rs3734851 | 6 | A | G | -0.5033 | 0.0584 | 6.575E-18 | 1.89E-02 | 74.26 |
| rs6933404 | 6 | T | C | 0.1486 | 0.0188 | 2.69E-15 | 1.69E-01 | 62.47 |
| rs9267798 | 6 | C | G | -0.2486 | 0.028 | 6.536E-19 | 6.76E-02 | 78.82 |
| rs9271176 | 6 | A | G | -0.3495 | 0.0173 | 4.2E-91 | 3.23E-01 | 408.07 |
| rs10272963 | 7 | T | C | 0.1512 | 0.016 | 4.107E-21 | 5.87E-01 | 89.29 |
| rs4728142 | 7 | A | G | -0.0995 | 0.0158 | 3.232E-10 | 4.49E-01 | 39.65 |
| rs798506 | 7 | T | C | -0.1206 | 0.0179 | 1.469E-11 | 7.23E-01 | 45.39 |
| rs989960 | 7 | T | C | 0.1214 | 0.016 | 3.285E-14 | 5.64E-01 | 57.56 |
| rs10817678 | 9 | A | G | -0.1332 | 0.017 | 4.418E-15 | 6.69E-01 | 61.38 |
| rs1887428 | 9 | C | G | 0.167 | 0.0166 | 9.652E-24 | 6.28E-01 | 101.19 |
| rs3812565 | 9 | T | C | 0.1335 | 0.016 | 6.5E-17 | 3.55E-01 | 69.61 |
| rs10761659 | 10 | A | G | 0.1276 | 0.016 | 1.327E-15 | 4.97E-01 | 63.59 |
| rs7911117 | 10 | T | G | -0.1342 | 0.0239 | 1.838E-08 | 1.51E-01 | 31.52 |
| rs7911680 | 10 | A | C | -0.1525 | 0.0159 | 6.707E-22 | 4.96E-01 | 91.98 |
| rs2045241 | 11 | A | G | 0.1063 | 0.0169 | 2.826E-10 | 3.35E-01 | 39.56 |
| rs2212434 | 11 | T | C | -0.1252 | 0.0159 | 2.804E-15 | 5.59E-01 | 61.99 |
| rs12825700 | 12 | A | G | -0.1889 | 0.0161 | 7.331E-32 | 3.60E-01 | 137.64 |
| rs1359946 | 13 | A | G | -0.1571 | 0.0202 | 6.577E-15 | 8.10E-01 | 60.48 |
| rs56062135 | 15 | T | C | -0.1078 | 0.0184 | 4.661E-09 | 7.85E-01 | 34.32 |
| rs11645239 | 16 | C | G | -0.1174 | 0.02 | 4.14E-09 | 8.09E-01 | 34.45 |
| rs16940186 | 16 | T | C | 0.1357 | 0.0214 | 2.185E-10 | 1.75E-01 | 40.2 |
| rs7203363 | 16 | A | T | -0.1071 | 0.0189 | 1.414E-08 | 7.91E-01 | 32.11 |
| rs11651246 | 17 | T | G | 0.147 | 0.0219 | 2.014E-11 | 1.49E-01 | 45.05 |
| rs12936409 | 17 | T | C | -0.1365 | 0.0158 | 5.62E-18 | 5.28E-01 | 74.62 |
| rs8073117 | 17 | A | G | 0.1548 | 0.0225 | 6.401E-12 | 1.25E-01 | 47.33 |
| rs10408351 | 19 | A | G | -0.1548 | 0.0204 | 2.918E-14 | 2.19E-01 | 57.57 |
| rs78064630 | 19 | A | G | -0.1759 | 0.0308 | 1.081E-08 | 7.55E-02 | 32.61 |
| rs6017342 | 20 | A | C | 0.1944 | 0.017 | 3.952E-30 | 4.80E-01 | 130.74 |
| rs6062496 | 20 | A | G | -0.1359 | 0.0163 | 8.966E-17 | 5.99E-01 | 69.5 |
| rs1736161 | 21 | A | G | 0.1227 | 0.0161 | 2.22E-14 | 4.41E-01 | 58.07 |
| rs2836881 | 21 | T | G | 0.2217 | 0.0186 | 1.105E-32 | 7.43E-01 | 142.05 |
| rs2838517 | 21 | T | C | -0.1177 | 0.016 | 1.777E-13 | 6.16E-01 | 54.11 |
| rs137845 | 22 | A | G | 0.1011 | 0.0158 | 1.502E-10 | 4.79E-01 | 40.94 |
| rs4993442 | 22 | T | G | 0.0988 | 0.0179 | 3.543E-08 | 2.73E-01 | 30.46 |
| rs9611131 | 22 | T | C | -0.1494 | 0.0227 | 5.106E-11 | 1.50E-01 | 43.31 |

Abbreviations: Beta, effect estimate; Chr, chromosome; EA, effect allele; EAF, effect allele frequency; OA, other allele; SE, standard error; SNP, single-nucleotide polymorphism.

**Supplementary Table S6. Baseline** **characteristics grouped by social isolation and loneliness.**

| **Characteristics** | **All**  **(n = 275,157)** | **Social isolation** | | | | **Loneliness** | | |
| --- | --- | --- | --- | --- | --- | --- | --- | --- |
|  |  | **Least isolated (n = 182,913)** | **Moderately isolated (n = 83,945)** | **Most isolated (n = 8,299)** | **P value** | **No loneliness (n = 264,338)** | **Loneliness (n = 10,819)** | **P value** |
| **IBD, n (%)** | 1565 (0.57) | 977 (0.53) | 526 (0.63) | 62 (0.75) | 0.001 | 1474 (0.56) | 91 (0.84) | <0.001 |
| **UC, n (%)** | 1063 (0.39) | 672 (0.37) | 350 (0.42) | 41 (0.49) | 0.043 | 998 (0.38) | 65 (0.60) | <0.001 |
| **CD, n (%)** | 492 (0.18) | 299 (0.16) | 172 (0.20) | 21 (0.25) | 0.016 | 466 (0.18) | 26 (0.24) | 0.15 |
| **Age, years** | 55.86 (8.11) | 56.18 (8.14) | 55.28 (8.02) | 54.49 (7.69) | <0.001 | 55.87 (8.11) | 55.42 (7.93) | <0.001 |
| **Male, n (%)** | 133959 (48.7) | 87296 (47.7) | 41555 (49.5) | 5108 (61.5) | <0.001 | 128044 (48.4) | 5915 (54.7) | <0.001 |
| **Ethnicity, white, n (%)** | 251904 (91.5) | 167957 (91.8) | 76742 (91.4) | 7205 (86.8) | <0.001 | 242083 (91.6) | 9821 (90.8) | 0.003 |
| **College/university degree, n (%)** | 104100 (37.8) | 71522 (39.1) | 29228 (34.8) | 3350 (40.4) | <0.001 | 101133 (38.3) | 2967 (27.4) | <0.001 |
| **Body mass index** |  |  |  |  | <0.001 |  |  | <0.001 |
| Normal, n (%) | 93470 (34.0) | 63117 (34.5) | 27641 (32.9) | 2712 (32.7) |  | 90524 (34.2) | 2946 (27.2) |  |
| Overweight, n (%) | 118546 (43.1) | 80158 (43.8) | 35014 (41.7) | 3374 (40.7) |  | 114131 (43.2) | 4415 (40.8) |  |
| Obese, n (%) | 63141 (22.9) | 39638 (21.7) | 21290 (25.4) | 2213 (26.7) |  | 59683 (22.6) | 3458 (32.0) |  |
| **Physical activity (MET), high, n (%) ^a^** | 140149 (50.9) | 100843 (55.1) | 36274 (43.2) | 3032 (36.5) | <0.001 | 135256 (51.2) | 4893 (45.2) | <0.001 |
| **Employed, n (%)** | 174365 (63.4) | 112159 (61.3) | 56263 (67.0) | 5943 (71.6) | <0.001 | 167915 (63.5) | 6450 (59.6) | <0.001 |
| **Household income, high, n (%)** | 81291 (29.5) | 54561 (29.8) | 24100 (28.7) | 2630 (31.7) | <0.001 | 79285 (30.0) | 2006 (18.5) | <0.001 |
| **TDI, n (%)** |  |  |  |  | <0.001 |  |  | <0.001 |
| Low deprivation | 97494 (35.4) | 67060 (36.7) | 27943 (33.3) | 2491 (30.0) |  | 94435 (35.7) | 3059 (28.3) |  |
| Moderate deprivation | 93910 (34.1) | 63208 (34.6) | 28004 (33.4) | 2698 (32.5) |  | 90603 (34.3) | 3307 (30.6) |  |
| High deprivation | 83753 (30.4) | 52645 (28.8) | 27998 (33.4) | 3110 (37.5) |  | 79300 (30.0) | 4453 (41.2) |  |
| **Current smoker, n (%)** | 27384 (10.0) | 15991 (8.7) | 10151 (12.1) | 1242 (15.0) | <0.001 | 25563 (9.7) | 1821 (16.8) | <0.001 |
| **Current drinker, n (%)** | 257572 (93.6) | 172835 (94.5) | 77371 (92.2) | 7366 (88.8) | <0.001 | 247752 (93.7) | 9820 (90.8) | <0.001 |
| **Healthy diet pattern, n (%) ^b^** | 93735 (34.1) | 63955 (35.0) | 27252 (32.5) | 2528 (30.5) | <0.001 | 90591 (34.3) | 3144 (29.1) | <0.001 |
| **Healthy sleep pattern, n (%) ^c^** | 163085 (59.3) | 110557 (60.4) | 48168 (57.4) | 4360 (52.5) | <0.001 | 158454 (59.9) | 4631 (42.8) | <0.001 |

MET: range 0–21, positively correlated with weekly physical activity; healthy diet score: range 0-5 points, positively linked with the level of adherence to a healthy diet; healthy sleep score: range 0-5 points, positively connected with the degree of adherence to a healthy sleep pattern.

^a^, Physical activity: MET score above the cohort median.

^b^, Healthy diet pattern: the healthy diet score ≥4.

^c^, Healthy sleep pattern: the healthy sleep score ≥4.

Abbreviation: BMI, body mass index; CD, Crohn’s disease; IBD, inflammatory bowel diseases; MET, metabolic equivalent of task; TDI, Townsend Deprivation Index; UC, ulcerative colitis.

Supplementary Table S7. The association of combined social isolation and loneliness with the risk of IBD.

| **Exposure** | **N** | **Cases/Person-years** | **Model 1 HR**  **(95% CI)** | **Model 2 HR**  **(95% CI)** | **Model 3 HR**  **(95% CI)** | **PAF (%)**  **(95% CI)** |
| --- | --- | --- | --- | --- | --- | --- |
| None | 177335 | 939/2397130 | 1.00 [Reference] | 1.00 [Reference] | 1.00 [Reference] |  |
| Social isolation or loneliness | 97822 | 626/1314088 | 1.22 (1.1-1.35) | 1.16 (1.04-1.28) | 1.15 (1.04-1.28) | 6.9 (4.99-8.82) |
| Social isolation and loneliness | 5241 | 53/69636 | 1.95 (1.48-2.57) | 1.62 (1.22-2.14) | 1.6 (1.21-2.12) | 2.55 (1.81-3.28) |

Social isolation was defined as being in the ‘moderately’ or ‘most’ isolated categories, whereas individuals classified as ‘none’ were considered not or less isolated. Model 1: adjusted for age, sex, and ethnicity; Model 2: adjusted for age, sex, ethnicity, BMI, household income, education level, employment status, smoking status, drinking status, physical activity, healthy diet pattern, and healthy sleep pattern; Model 3: adjusted for age, sex, ethnicity, BMI, household income, education level, employment status, smoking status, drinking status, physical activity, healthy diet pattern, and healthy sleep pattern, and PRS for IBD. Abbreviation: BMI, body mass index; HR, hazard ratio; IBD, inflammatory bowel disease; PAF, population attributable fraction; PRS, polygenic risk score.

**Supplementary Table S8.** Separate and joint associations of social isolation and loneliness with long-term risks of UC and CD.

| **Subtype** | **Exposure** | **N** | **Cases/Person-years** | **Model1 HR (95% CI)** | **Model2 HR (95% CI)** | **Model3 HR (95% CI)** | **PAF (%) (95% CI [%])** |
| --- | --- | --- | --- | --- | --- | --- | --- |
| **UC** | **Separate effects** |  |  |  |  |  |  |
|  | Social isolation |  |  |  |  |  | 4.86 (2.65-7.08) |
|  | Least isolated | 182608 | 672/2469728 | 1.00 [Reference] | 1.00 [Reference] | 1.00 [Reference] |  |
|  | Moderately isolated | 83769 | 350/1126617 | 1.15 (1.01-1.3) | 1.11 (0.97-1.26) | 1.11 (0.97-1.26) |  |
|  | Most isolated | 8278 | 41/110459 | 1.32 (0.96-1.81) | 1.26 (0.92-1.74) | 1.26 (0.92-1.74) |  |
|  | **P trend** |  |  | 0.012 | 0.051 | 0.053 |  |
|  | Loneliness |  |  |  |  |  | 2.27 (1.51-3.03) |
|  | No loneliness | 263862 | 998/3562193 | 1.00 [Reference] | 1.00 [Reference] | 1.00 [Reference] |  |
|  | Loneliness | 10793 | 65/144612 | 1.58 (1.23-2.03) | 1.39 (1.08-1.79) | 1.39 (1.08-1.79) |  |
|  | **Joint effects** |  |  |  |  |  |  |
|  | No loneliness |  |  |  |  |  |  |
|  | Least isolated | 177039 | 643/2394574 | 1.00 [Reference] | 1.00 [Reference] | 1.00 [Reference] |  |
|  | Moderately isolated | 79466 | 323/1069284 | 1.13 (0.99-1.29) | 1.1 (0.96-1.26) | 1.10 (0.96-1.25) |  |
|  | Most isolated | 7357 | 32/98335 | 1.17 (0.82-1.67) | 1.15 (0.8-1.64) | 1.14 (0.8-1.63) |  |
|  | Loneliness |  |  |  |  |  |  |
|  | Less isolated | 5569 | 29/75155 | 1.41 (0.97-2.04) | 1.27 (0.88-1.85) | 1.26 (0.87-1.83) |  |
|  | Moderately isolated | 4303 | 27/57333 | 1.74 (1.18-2.56) | 1.51 (1.03-2.23) | 1.49 (1.01-2.19) |  |
|  | Most isolated | 921 | 9/12124 | 2.7 (1.4-5.22) | 2.3 (1.19-4.46) | 2.27 (1.17-4.40) |  |
|  | **P trend** |  |  | <0.001 | 0.001 | 0.002 |  |
| **CD** | **Separate effects** |  |  |  |  |  |  |
|  | Social isolation |  |  |  |  |  | 8.6 (5.29-11.91) |
|  | Least isolated | 182235 | 299/2466843 | 1.00 [Reference] | 1.00 [Reference] | 1.00 [Reference] |  |
|  | Moderately isolated | 83591 | 172/1125415 | 1.26 (1.05-1.52) | 1.19 (0.98-1.43) | 1.18 (0.98-1.43) |  |
|  | Most isolated | 8258 | 21/110309 | 1.6 (1.02-2.49) | 1.45 (0.93-2.26) | 1.44 (0.92-2.25) |  |
|  | **P trend** |  |  | 0.002 | 0.027 | 0.029 |  |
|  | Loneliness |  |  |  |  |  | 1.42 (0.37-2.47) |
|  | No loneliness | 263330 | 466/3558245 | 1.00 [Reference] | 1.00 [Reference] | 1.00 [Reference] |  |
|  | Loneliness | 10754 | 26/144323 | 1.37 (0.93-2.04) | 1.14 (0.76-1.69) | 1.13 (0.76-1.68) |  |
|  | **Joint effects** |  |  |  |  |  |  |
|  | No loneliness |  |  |  |  |  |  |
|  | Least isolated | 176686 | 290/2391883 | 1.00 [Reference] | 1.00 [Reference] | 1.00 [Reference] |  |
|  | Moderately isolated | 79300 | 157/1068135 | 1.21 (1-1.48) | 1.15 (0.94-1.4) | 1.15 (0.94-1.4) |  |
|  | Most isolated | 7344 | 19/98227 | 1.62 (1.02-2.58) | 1.51 (0.94-2.41) | 1.5 (0.94-2.39) |  |
|  | Loneliness |  |  |  |  |  |  |
|  | Less isolated | 5549 | 9/74961 | 0.99 (0.51-1.92) | 0.85 (0.44-1.65) | 0.85 (0.43-1.64) |  |
|  | Moderately isolated | 4291 | 15/57280 | 2.16 (1.29-3.64) | 1.69 (1-2.86) | 1.68 (0.99-2.83) |  |
|  | Most isolated | 914 | 2/12082 | 1.38 (0.34-5.56) | 1.02 (0.25-4.13) | 1.02 (0.25-4.11) |  |
|  | **P trend** |  |  | 0.002 | 0.065 | 0.07 |  |

Model 1 adjusted for age, sex, and ethnicity; Model 2 further adjusted for BMI, household income, educational level, employment status, smoking status, alcohol consumption, physical activity, TDI, healthy diet pattern, and healthy sleep pattern; Model 3 additionally adjusted for PRS of UC or CD. Abbreviations: BMI, body mass index; CD, Crohn’s disease; HR, hazard ratio; PAF, population attributable fraction; PRS, polygenic risk score; TDI, Townsend Deprivation Index; UC, ulcerative colitis.

Supplementary Table S9. Association of combined social isolation and loneliness with the risk of IBD stratified by genetic susceptibility.

| **PRS stratification** | **Group** | **N** | **Cases/Person-years** | **Model 1 HR**  **(95% CI)** | **Model 2 HR**  **(95% CI)** |
| --- | --- | --- | --- | --- | --- |
| **Low genetic risk** | None | 44209 | 144/597609 | 1.00 [Reference] | 1.00 [Reference] |
|  | Isolation or loneliness | 24363 | 89/327692 | 1.15 (0.88-1.5) | 1.07 (0.82-1.41) |
|  | Isolation and loneliness | 1287 | 6/17041 | 1.51 (0.67-3.42) | 1.16 (0.51-2.67) |
| **Intermediate genetic risk** | None | 89009 | 467/1204073 | 1.00 [Reference] | 1.00 [Reference] |
|  | Isolation or loneliness | 48998 | 317/658253 | 1.24 (1.08-1.43) | 1.18 (1.02-1.37) |
|  | Isolation and loneliness | 2612 | 27/34679 | 2.01 (1.37-2.97) | 1.68 (1.13-2.49) |
| **High genetic risk** | None | 44117 | 328/595447 | 1.00 [Reference] | 1.00 [Reference] |
|  | Isolation or loneliness | 24461 | 220/328142 | 1.21 (1.02-1.43) | 1.15 (0.97-1.37) |
|  | Isolation and loneliness | 1342 | 20/17915 | 1.99 (1.26-3.12) | 1.68 (1.06-2.67) |

Social isolation was defined as being in the ‘moderately’ or ‘most’ isolated categories, whereas individuals classified as ‘none’ were considered not or less isolated. Grades of genetic risk are defined as low, intermediate, and high according to Q1, Q2-3, and Q4 of PRS for IBD. Model 1: adjusted for age, sex, and ethnicity; Model 2: adjusted for age, sex, ethnicity, BMI, household income, education level, employment status, smoking status, drinking status, physical activity, healthy diet pattern, and healthy sleep pattern. Abbreviation: BMI, body mass index; HR, hazard ratio; IBD, inflammatory bowel disease; PRS, polygenic risk scores.

**Supplementary Table S10.** Metabolites associated with social isolation and loneliness.

| **Metabolites** | **Coefficient** | **SE** | **P** | **P for FDR** |
| --- | --- | --- | --- | --- |
| **Loneliness** |  |  |  |  |
| Total cholesterol | -0.049 | 0.019 | 0.011 | 0.082 |
| Total cholesterol minus HDL-C | -0.042 | 0.020 | 0.035 | 0.138 |
| Clinical LDL cholesterol | -0.052 | 0.020 | 0.009 | 0.079 |
| LDL cholesterol | -0.048 | 0.020 | 0.016 | 0.093 |
| HDL cholesterol | -0.034 | 0.017 | 0.045 | 0.162 |
| Triglycerides in LDL | 0.046 | 0.019 | 0.019 | 0.100 |
| Phospholipids in LDL | -0.044 | 0.020 | 0.028 | 0.129 |
| Total esterified cholesterol | -0.052 | 0.019 | 0.007 | 0.074 |
| Cholesteryl esters in LDL | -0.044 | 0.020 | 0.028 | 0.129 |
| Cholesteryl esters in HDL | -0.037 | 0.017 | 0.031 | 0.133 |
| Total free cholesterol | -0.042 | 0.020 | 0.032 | 0.133 |
| Free cholesterol in LDL | -0.057 | 0.020 | 0.004 | 0.070 |
| Total lipids in LDL | -0.042 | 0.020 | 0.034 | 0.137 |
| Total concentration of lipoprotein particles | -0.047 | 0.019 | 0.013 | 0.082 |
| Concentration of HDL particles | -0.044 | 0.019 | 0.018 | 0.098 |
| Degree of unsaturation | -0.117 | 0.018 | <0.001 | <0.001 |
| Omega-3 fatty acids | -0.071 | 0.020 | <0.001 | 0.010 |
| Omega-6 fatty acids | -0.045 | 0.020 | 0.021 | 0.105 |
| Polyunsaturated fatty acids | -0.058 | 0.020 | 0.003 | 0.070 |
| Linoleic acid | -0.041 | 0.020 | 0.039 | 0.145 |
| Docosahexaenoic acid | -0.081 | 0.019 | <0.001 | 0.001 |
| Histidine | -0.049 | 0.020 | 0.013 | 0.082 |
| Glucose | 0.055 | 0.019 | 0.004 | 0.070 |
| Acetate | 0.110 | 0.020 | <0.001 | <0.001 |
| Albumin | -0.074 | 0.020 | <0.001 | 0.007 |
| Glycoprotein acetyls | 0.039 | 0.018 | 0.033 | 0.134 |
| Cholesterol in medium VLDL | -0.040 | 0.020 | 0.043 | 0.156 |
| Cholesteryl esters in medium VLDL | -0.047 | 0.020 | 0.018 | 0.098 |
| Triglycerides in very small VLDL | 0.052 | 0.019 | 0.007 | 0.074 |
| Total lipids in IDL | -0.041 | 0.019 | 0.032 | 0.133 |
| Cholesterol in IDL | -0.052 | 0.019 | 0.007 | 0.074 |
| Cholesteryl esters in IDL | -0.052 | 0.019 | 0.007 | 0.074 |
| Free cholesterol in IDL | -0.051 | 0.019 | 0.008 | 0.074 |
| Triglycerides in IDL | 0.052 | 0.020 | 0.008 | 0.074 |
| Total lipids in large LDL | -0.046 | 0.020 | 0.021 | 0.105 |
| Phospholipids in large LDL | -0.049 | 0.020 | 0.013 | 0.082 |
| Cholesterol in large LDL | -0.051 | 0.020 | 0.010 | 0.081 |
| Cholesteryl esters in large LDL | -0.048 | 0.020 | 0.016 | 0.093 |
| Free cholesterol in large LDL | -0.057 | 0.020 | 0.003 | 0.070 |
| Triglycerides in large LDL | 0.047 | 0.020 | 0.015 | 0.093 |
| Cholesterol in medium LDL | -0.041 | 0.020 | 0.038 | 0.145 |
| Free cholesterol in medium LDL | -0.056 | 0.020 | 0.005 | 0.070 |
| Triglycerides in medium LDL | 0.043 | 0.019 | 0.025 | 0.121 |
| Cholesterol in small LDL | -0.040 | 0.020 | 0.046 | 0.162 |
| Free cholesterol in small LDL | -0.053 | 0.020 | 0.008 | 0.074 |
| Cholesteryl esters in medium HDL | -0.036 | 0.018 | 0.047 | 0.162 |
| Concentration of small HDL particles | -0.050 | 0.020 | 0.012 | 0.082 |
| Cholesterol in small HDL | -0.051 | 0.020 | 0.010 | 0.081 |
| Cholesteryl esters in small HDL | -0.057 | 0.020 | 0.004 | 0.070 |
| **Social isolation** |  |  |  |  |
| Total cholesterol | -0.018 | 0.007 | 0.010 | 0.017 |
| VLDL cholesterol | 0.023 | 0.007 | 0.001 | 0.003 |
| HDL cholesterol | -0.063 | 0.006 | <0.001 | <0.001 |
| Total triglycerides | 0.017 | 0.007 | 0.011 | 0.020 |
| Triglycerides in VLDL | 0.019 | 0.007 | 0.005 | 0.009 |
| Triglycerides in LDL | 0.015 | 0.007 | 0.030 | 0.046 |
| Total phospholipids in lipoprotein particles | -0.034 | 0.007 | <0.001 | <0.001 |
| Phospholipids in VLDL | 0.025 | 0.007 | <0.001 | <0.001 |
| Phospholipids in HDL | -0.067 | 0.006 | <0.001 | <0.001 |
| Total esterified cholesterol | -0.021 | 0.007 | 0.002 | 0.005 |
| Cholesteryl esters in VLDL | 0.021 | 0.007 | 0.003 | 0.006 |
| Cholesteryl esters in HDL | -0.063 | 0.006 | <0.001 | <0.001 |
| Free cholesterol in VLDL | 0.024 | 0.007 | <0.001 | 0.001 |
| Free cholesterol in HDL | -0.062 | 0.006 | <0.001 | <0.001 |
| Total lipids in lipoprotein particles | -0.014 | 0.007 | 0.049 | 0.071 |
| Total lipids in VLDL | 0.023 | 0.007 | 0.001 | 0.003 |
| Total lipids in HDL | -0.066 | 0.006 | <0.001 | <0.001 |
| Total concentration of lipoprotein particles | -0.054 | 0.007 | <0.001 | <0.001 |
| Concentration of VLDL particles | 0.022 | 0.007 | 0.002 | 0.004 |
| Concentration of LDL particles | 0.017 | 0.007 | 0.019 | 0.031 |
| Concentration of HDL particles | -0.059 | 0.007 | <0.001 | <0.001 |
| Average diameter for VLDL particles | 0.027 | 0.006 | <0.001 | <0.001 |
| Average diameter for HDL particles | -0.058 | 0.006 | <0.001 | <0.001 |
| Phosphoglycerides | -0.044 | 0.007 | <0.001 | <0.001 |
| Total cholines | -0.042 | 0.007 | <0.001 | <0.001 |
| Phosphatidylcholines | -0.047 | 0.007 | <0.001 | <0.001 |
| Sphingomyelins | -0.028 | 0.007 | <0.001 | <0.001 |
| Apolipoprotein B | 0.016 | 0.007 | 0.027 | 0.042 |
| Apolipoprotein A1 | -0.064 | 0.007 | <0.001 | <0.001 |
| Degree of unsaturation | -0.050 | 0.007 | <0.001 | <0.001 |
| Omega-3 fatty acids | -0.047 | 0.007 | <0.001 | <0.001 |
| Polyunsaturated fatty acids | -0.024 | 0.007 | <0.001 | 0.001 |
| Docosahexaenoic acid | -0.056 | 0.007 | <0.001 | <0.001 |
| Total concentration of branched-chain amino acids (leucine + isoleucine + valine) | -0.017 | 0.007 | 0.017 | 0.027 |
| Leucine | -0.017 | 0.007 | 0.016 | 0.026 |
| Valine | -0.018 | 0.007 | 0.009 | 0.016 |
| Tyrosine | -0.025 | 0.007 | <0.001 | 0.001 |
| Lactate | 0.067 | 0.007 | <0.001 | <0.001 |
| Pyruvate | 0.038 | 0.007 | <0.001 | <0.001 |
| Citrate | 0.042 | 0.007 | <0.001 | <0.001 |
| 3-Hydroxybutyrate | 0.021 | 0.007 | 0.004 | 0.008 |
| Acetone | 0.016 | 0.007 | 0.030 | 0.046 |
| Glycoprotein acetyls | 0.040 | 0.007 | <0.001 | <0.001 |
| Concentration of chylomicrons and extremely large VLDL particles | 0.019 | 0.007 | 0.004 | 0.008 |
| Total lipids in chylomicrons and extremely large VLDL | 0.019 | 0.007 | 0.005 | 0.010 |
| Phospholipids in chylomicrons and extremely large VLDL | 0.021 | 0.007 | 0.002 | 0.004 |
| Cholesterol in chylomicrons and extremely large VLDL | 0.022 | 0.007 | 0.001 | 0.003 |
| Cholesteryl esters in chylomicrons and extremely large VLDL | 0.023 | 0.007 | <0.001 | 0.001 |
| Free cholesterol in chylomicrons and extremely large VLDL | 0.020 | 0.007 | 0.002 | 0.005 |
| Triglycerides in chylomicrons and extremely large VLDL | 0.017 | 0.007 | 0.012 | 0.021 |
| Concentration of very large VLDL particles | 0.026 | 0.007 | <0.001 | <0.001 |
| Total lipids in very large VLDL | 0.023 | 0.007 | <0.001 | 0.001 |
| Phospholipids in very large VLDL | 0.027 | 0.007 | <0.001 | <0.001 |
| Cholesterol in very large VLDL | 0.030 | 0.007 | <0.001 | <0.001 |
| Cholesteryl esters in very large VLDL | 0.032 | 0.007 | <0.001 | <0.001 |
| Free cholesterol in very large VLDL | 0.027 | 0.007 | <0.001 | <0.001 |
| Triglycerides in very large VLDL | 0.020 | 0.007 | 0.003 | 0.007 |
| Concentration of large VLDL particles | 0.026 | 0.007 | <0.001 | <0.001 |
| Total lipids in large VLDL | 0.025 | 0.007 | <0.001 | <0.001 |
| Phospholipids in large VLDL | 0.029 | 0.007 | <0.001 | <0.001 |
| Cholesterol in large VLDL | 0.031 | 0.007 | <0.001 | <0.001 |
| Cholesteryl esters in large VLDL | 0.031 | 0.007 | <0.001 | <0.001 |
| Free cholesterol in large VLDL | 0.030 | 0.007 | <0.001 | <0.001 |
| Triglycerides in large VLDL | 0.019 | 0.007 | 0.005 | 0.009 |
| Concentration of medium VLDL particles | 0.023 | 0.007 | 0.001 | 0.003 |
| Total lipids in medium VLDL | 0.021 | 0.007 | 0.003 | 0.006 |
| Phospholipids in medium VLDL | 0.021 | 0.007 | 0.003 | 0.006 |
| Cholesterol in medium VLDL | 0.017 | 0.007 | 0.019 | 0.031 |
| Free cholesterol in medium VLDL | 0.020 | 0.007 | 0.006 | 0.012 |
| Triglycerides in medium VLDL | 0.019 | 0.007 | 0.004 | 0.009 |
| Concentration of small VLDL particles | 0.027 | 0.007 | <0.001 | <0.001 |
| Total lipids in small VLDL | 0.024 | 0.007 | <0.001 | 0.001 |
| Phospholipids in small VLDL | 0.023 | 0.007 | 0.001 | 0.002 |
| Cholesterol in small VLDL | 0.027 | 0.007 | <0.001 | <0.001 |
| Cholesteryl esters in small VLDL | 0.030 | 0.007 | <0.001 | <0.001 |
| Free cholesterol in small VLDL | 0.021 | 0.007 | 0.003 | 0.006 |
| Triglycerides in small VLDL | 0.017 | 0.007 | 0.012 | 0.021 |
| Triglycerides in very small VLDL | 0.014 | 0.007 | 0.042 | 0.063 |
| Cholesterol in IDL | -0.015 | 0.007 | 0.027 | 0.042 |
| Cholesteryl esters in IDL | -0.017 | 0.007 | 0.015 | 0.024 |
| Concentration of large LDL particles | 0.017 | 0.007 | 0.019 | 0.030 |
| Phospholipids in medium LDL | 0.014 | 0.007 | 0.045 | 0.067 |
| Triglycerides in medium LDL | 0.018 | 0.007 | 0.009 | 0.016 |
| Concentration of small LDL particles | 0.020 | 0.007 | 0.005 | 0.010 |
| Total lipids in small LDL | 0.018 | 0.007 | 0.014 | 0.024 |
| Phospholipids in small LDL | 0.019 | 0.007 | 0.007 | 0.012 |
| Cholesterol in small LDL | 0.015 | 0.007 | 0.034 | 0.051 |
| Cholesteryl esters in small LDL | 0.016 | 0.007 | 0.022 | 0.034 |
| Triglycerides in small LDL | 0.019 | 0.007 | 0.006 | 0.011 |
| Concentration of very large HDL particles | -0.039 | 0.006 | <0.001 | <0.001 |
| Total lipids in very large HDL | -0.041 | 0.006 | <0.001 | <0.001 |
| Phospholipids in very large HDL | -0.042 | 0.006 | <0.001 | <0.001 |
| Cholesterol in very large HDL | -0.040 | 0.006 | <0.001 | <0.001 |
| Cholesteryl esters in very large HDL | -0.042 | 0.006 | <0.001 | <0.001 |
| Free cholesterol in very large HDL | -0.028 | 0.006 | <0.001 | <0.001 |
| Concentration of large HDL particles | -0.056 | 0.006 | <0.001 | <0.001 |
| Total lipids in large HDL | -0.058 | 0.006 | <0.001 | <0.001 |
| Phospholipids in large HDL | -0.061 | 0.006 | <0.001 | <0.001 |
| Cholesterol in large HDL | -0.055 | 0.006 | <0.001 | <0.001 |
| Cholesteryl esters in large HDL | -0.055 | 0.006 | <0.001 | <0.001 |
| Free cholesterol in large HDL | -0.055 | 0.006 | <0.001 | <0.001 |
| Triglycerides in large HDL | -0.028 | 0.007 | <0.001 | <0.001 |
| Concentration of medium HDL particles | -0.066 | 0.007 | <0.001 | <0.001 |
| Total lipids in medium HDL | -0.066 | 0.007 | <0.001 | <0.001 |
| Phospholipids in medium HDL | -0.066 | 0.007 | <0.001 | <0.001 |
| Cholesterol in medium HDL | -0.067 | 0.007 | <0.001 | <0.001 |
| Cholesteryl esters in medium HDL | -0.066 | 0.007 | <0.001 | <0.001 |
| Free cholesterol in medium HDL | -0.067 | 0.006 | <0.001 | <0.001 |
| Concentration of small HDL particles | -0.026 | 0.007 | <0.001 | <0.001 |
| Total lipids in small HDL | -0.032 | 0.007 | <0.001 | <0.001 |
| Phospholipids in small HDL | -0.038 | 0.007 | <0.001 | <0.001 |
| Cholesterol in small HDL | -0.031 | 0.007 | <0.001 | <0.001 |
| Cholesteryl esters in small HDL | -0.028 | 0.007 | <0.001 | <0.001 |
| Free cholesterol in small HDL | -0.037 | 0.007 | <0.001 | <0.001 |
| Triglycerides in small HDL | 0.020 | 0.007 | 0.004 | 0.007 |

Abbreviation: FDR, false discovery rate; SE, standard error.

**Supplementary Table S11.** FDR-significant circulating proteins associated with social isolation.

| **Proteins** | **Coefficient** | **SE** | **P for FDR** | **Proteins** | **Coefficient** | **SE** | **P for FDR** |
| --- | --- | --- | --- | --- | --- | --- | --- |
| A1BG | 0.007 | 0.002 | 0.008 | ITGA5 | 0.011 | 0.003 | 0.002 |
| ABL1 | 0.024 | 0.008 | 0.023 | ITGAV | -0.011 | 0.002 | <0.001 |
| ACVRL1 | 0.015 | 0.003 | <0.001 | ITGB1BP1 | 0.018 | 0.005 | 0.003 |
| ADA2 | 0.017 | 0.005 | 0.008 | JAM2 | 0.009 | 0.003 | 0.036 |
| ADAM12 | 0.017 | 0.004 | 0.001 | KCTD5 | 0.015 | 0.005 | 0.026 |
| ADAM22 | 0.023 | 0.004 | <0.001 | KIAA0319 | 0.015 | 0.004 | 0.001 |
| ADAM8 | 0.015 | 0.003 | <0.001 | KLK4 | 0.026 | 0.008 | 0.006 |
| ADAMTS16 | 0.010 | 0.004 | 0.049 | KRT18 | 0.048 | 0.010 | <0.001 |
| ADGRG1 | 0.023 | 0.008 | 0.028 | KYNU | 0.015 | 0.006 | 0.039 |
| ADH1B | 0.024 | 0.009 | 0.047 | LAIR1 | 0.017 | 0.005 | 0.002 |
| ADH4 | 0.029 | 0.010 | 0.026 | LAMP3 | 0.018 | 0.006 | 0.030 |
| AGER | 0.017 | 0.005 | 0.006 | LAMTOR5 | 0.013 | 0.005 | 0.039 |
| AGRN | 0.021 | 0.004 | <0.001 | LAT | 0.026 | 0.009 | 0.031 |
| AHNAK | 0.013 | 0.004 | 0.008 | LAYN | 0.014 | 0.004 | 0.007 |
| AHSG | 0.013 | 0.003 | 0.001 | LBR | 0.037 | 0.008 | <0.001 |
| AIFM1 | 0.040 | 0.013 | 0.012 | LCN2 | 0.023 | 0.005 | <0.001 |
| AKR1C4 | 0.021 | 0.007 | 0.012 | LDLR | 0.023 | 0.006 | 0.003 |
| AKT1S1 | 0.021 | 0.008 | 0.044 | LEG1 | -0.025 | 0.008 | 0.021 |
| AKT3 | 0.019 | 0.007 | 0.040 | LEP | 0.028 | 0.009 | 0.009 |
| ALCAM | 0.015 | 0.002 | <0.001 | LGALS1 | 0.019 | 0.004 | <0.001 |
| ALDH1A1 | 0.022 | 0.007 | 0.007 | LGALS3BP | 0.011 | 0.004 | 0.020 |
| AMBP | 0.008 | 0.002 | 0.010 | LGALS4 | 0.020 | 0.007 | 0.017 |
| AMN | 0.019 | 0.006 | 0.007 | LGALS9 | 0.016 | 0.004 | <0.001 |
| AMOT | 0.021 | 0.005 | <0.001 | LILRA2 | 0.013 | 0.004 | 0.016 |
| ANGPTL4 | 0.018 | 0.006 | 0.008 | LILRA5 | 0.011 | 0.004 | 0.028 |
| ANXA3 | 0.025 | 0.009 | 0.024 | LILRB2 | 0.017 | 0.004 | 0.001 |
| APBB1IP | 0.036 | 0.006 | <0.001 | LMNB1 | 0.022 | 0.007 | 0.018 |
| APCS | 0.012 | 0.004 | 0.020 | LMNB2 | 0.016 | 0.003 | <0.001 |
| APEX1 | 0.030 | 0.007 | <0.001 | LPCAT2 | 0.015 | 0.005 | 0.018 |
| APOA1 | -0.011 | 0.003 | 0.004 | LPL | -0.015 | 0.006 | 0.049 |
| APOA4 | 0.013 | 0.004 | 0.020 | LPO | -0.026 | 0.008 | 0.006 |
| APOC1 | -0.018 | 0.005 | 0.001 | LRCH4 | 0.024 | 0.009 | 0.026 |
| APOD | -0.014 | 0.005 | 0.041 | LRIG1 | 0.014 | 0.004 | 0.006 |
| APOF | -0.017 | 0.003 | <0.001 | LRP11 | 0.017 | 0.004 | <0.001 |
| APOH | 0.014 | 0.004 | 0.014 | LRRC25 | 0.026 | 0.005 | <0.001 |
| APOM | -0.015 | 0.003 | <0.001 | LRRN1 | 0.014 | 0.005 | 0.021 |
| AREG | 0.027 | 0.006 | <0.001 | LSP1 | 0.028 | 0.005 | <0.001 |
| ARSB | 0.029 | 0.007 | <0.001 | LTA4H | 0.028 | 0.007 | 0.001 |
| ASAH2 | 0.022 | 0.007 | 0.006 | LTBP3 | 0.021 | 0.007 | 0.014 |
| ASGR1 | 0.012 | 0.004 | 0.010 | LTBR | 0.019 | 0.003 | <0.001 |
| ATOX1 | 0.026 | 0.009 | 0.017 | LYN | 0.027 | 0.010 | 0.045 |
| AXL | 0.014 | 0.003 | <0.001 | LYVE1 | 0.007 | 0.002 | 0.030 |
| AZU1 | 0.032 | 0.010 | 0.008 | MAD1L1 | 0.029 | 0.006 | <0.001 |
| B2M | 0.010 | 0.004 | 0.040 | MAMDC2 | 0.014 | 0.004 | 0.006 |
| BAP18 | 0.021 | 0.008 | 0.049 | MAMDC4 | -0.041 | 0.007 | <0.001 |
| BAX | 0.039 | 0.011 | 0.004 | MANSC1 | 0.014 | 0.003 | <0.001 |
| BCL2L15 | 0.022 | 0.007 | 0.015 | MANSC4 | 0.026 | 0.008 | 0.011 |
| BID | 0.033 | 0.008 | <0.001 | MAP2 | 0.016 | 0.006 | 0.022 |
| BMPER | 0.009 | 0.003 | 0.006 | MAP2K1 | 0.029 | 0.008 | 0.003 |
| BNIP3L | 0.018 | 0.006 | 0.009 | MAPK13 | 0.015 | 0.005 | 0.025 |
| BOC | 0.009 | 0.003 | 0.010 | MAPK9 | 0.014 | 0.005 | 0.043 |
| BPIFB1 | -0.029 | 0.008 | 0.003 | MAX | 0.035 | 0.013 | 0.033 |
| BPIFB2 | 0.049 | 0.008 | <0.001 | MDH1 | 0.015 | 0.005 | 0.020 |
| BSG | 0.007 | 0.002 | 0.020 | MED18 | 0.025 | 0.009 | 0.042 |
| BTN2A1 | 0.015 | 0.003 | <0.001 | MEGF10 | -0.022 | 0.005 | <0.001 |
| BTNL10 | 0.014 | 0.005 | 0.030 | MERTK | 0.016 | 0.003 | <0.001 |
| C1QA | 0.009 | 0.003 | 0.010 | MFAP5 | 0.016 | 0.005 | 0.011 |
| C1R | 0.007 | 0.002 | 0.025 | MILR1 | 0.019 | 0.006 | 0.017 |
| C1RL | 0.012 | 0.002 | <0.001 | MMP12 | 0.025 | 0.007 | 0.001 |
| C1S | 0.009 | 0.003 | 0.005 | MMP8 | 0.036 | 0.009 | <0.001 |
| C2 | 0.008 | 0.003 | 0.031 | MMP9 | 0.035 | 0.007 | <0.001 |
| C4BPB | 0.023 | 0.005 | <0.001 | MNDA | 0.035 | 0.013 | 0.025 |
| C9 | 0.018 | 0.006 | 0.024 | MPI | 0.028 | 0.011 | 0.041 |
| CA12 | 0.012 | 0.004 | 0.014 | MPO | 0.027 | 0.006 | <0.001 |
| CA14 | -0.033 | 0.004 | <0.001 | MSTN | 0.035 | 0.006 | <0.001 |
| CA3 | 0.019 | 0.008 | 0.046 | MTDH | 0.034 | 0.011 | 0.017 |
| CA6 | -0.034 | 0.008 | <0.001 | MXRA8 | -0.014 | 0.004 | 0.002 |
| CA9 | -0.014 | 0.006 | 0.041 | MYOC | -0.015 | 0.006 | 0.047 |
| CALB1 | 0.022 | 0.005 | <0.001 | MZB1 | -0.018 | 0.006 | 0.020 |
| CALB2 | 0.010 | 0.004 | 0.029 | NAAA | 0.034 | 0.007 | <0.001 |
| CAMKK1 | 0.014 | 0.006 | 0.042 | NADK | 0.028 | 0.007 | <0.001 |
| CAPG | 0.025 | 0.007 | 0.004 | NAGA | 0.023 | 0.007 | 0.011 |
| CASP1 | 0.022 | 0.008 | 0.042 | NAMPT | 0.024 | 0.006 | 0.003 |
| CASP8 | 0.023 | 0.008 | 0.018 | NBL1 | 0.011 | 0.003 | 0.004 |
| CBLN4 | 0.013 | 0.004 | 0.009 | NBN | 0.024 | 0.007 | 0.004 |
| CC2D1A | 0.025 | 0.009 | 0.039 | NCF2 | 0.044 | 0.011 | <0.001 |
| CCDC80 | 0.013 | 0.004 | 0.017 | NCR1 | 0.021 | 0.004 | <0.001 |
| CCER2 | 0.033 | 0.008 | <0.001 | NCR3LG1 | 0.019 | 0.004 | <0.001 |
| CCL14 | 0.015 | 0.005 | 0.017 | NECTIN2 | 0.015 | 0.004 | <0.001 |
| CCL15 | 0.015 | 0.005 | 0.034 | NEDD4L | 0.022 | 0.006 | 0.003 |
| CCL21 | 0.013 | 0.005 | 0.046 | NEO1 | 0.008 | 0.003 | 0.020 |
| CCL22 | 0.033 | 0.006 | <0.001 | NFKBIE | 0.025 | 0.006 | <0.001 |
| CCL23 | 0.017 | 0.005 | 0.009 | NFYA | 0.016 | 0.005 | 0.006 |
| CCL26 | 0.036 | 0.011 | 0.010 | NGFR | -0.009 | 0.004 | 0.039 |
| CCN4 | 0.013 | 0.005 | 0.032 | NMNAT1 | 0.040 | 0.009 | <0.001 |
| CCN5 | 0.013 | 0.005 | 0.042 | NOS3 | 0.025 | 0.006 | 0.001 |
| CCS | 0.015 | 0.006 | 0.050 | NOTCH1 | 0.005 | 0.002 | 0.029 |
| CCT5 | 0.015 | 0.006 | 0.041 | NPDC1 | 0.017 | 0.004 | <0.001 |
| CD163 | 0.014 | 0.005 | 0.041 | NPPC | 0.035 | 0.006 | <0.001 |
| CD164 | 0.010 | 0.003 | 0.015 | NRCAM | 0.014 | 0.003 | <0.001 |
| CD200 | 0.011 | 0.003 | 0.004 | NRP2 | 0.010 | 0.004 | 0.025 |
| CD207 | 0.016 | 0.005 | 0.004 | NSFL1C | 0.026 | 0.008 | 0.010 |
| CD22 | 0.022 | 0.005 | <0.001 | NT5C1A | 0.017 | 0.004 | <0.001 |
| CD244 | 0.009 | 0.003 | 0.039 | NTRK3 | -0.015 | 0.003 | <0.001 |
| CD276 | 0.013 | 0.004 | 0.015 | NUCB2 | 0.015 | 0.005 | 0.007 |
| CD28 | 0.019 | 0.004 | <0.001 | NUDC | 0.022 | 0.008 | 0.030 |
| CD300A | 0.015 | 0.004 | <0.001 | NUDT5 | 0.022 | 0.006 | 0.004 |
| CD300C | 0.020 | 0.004 | <0.001 | NXPH3 | 0.011 | 0.004 | 0.025 |
| CD300E | 0.015 | 0.005 | 0.015 | OCLN | 0.020 | 0.005 | 0.002 |
| CD300LF | 0.022 | 0.008 | 0.021 | OGFR | 0.016 | 0.004 | 0.001 |
| CD302 | 0.018 | 0.004 | <0.001 | OGN | 0.024 | 0.005 | <0.001 |
| CD38 | 0.011 | 0.004 | 0.030 | OLR1 | 0.037 | 0.007 | <0.001 |
| CD4 | 0.015 | 0.004 | <0.001 | OSCAR | 0.026 | 0.004 | <0.001 |
| CD46 | 0.012 | 0.003 | 0.004 | OSM | 0.046 | 0.009 | <0.001 |
| CD5 | 0.017 | 0.004 | <0.001 | OSMR | 0.009 | 0.002 | 0.003 |
| CD58 | 0.006 | 0.002 | 0.027 | PADI2 | 0.020 | 0.007 | 0.025 |
| CD59 | 0.008 | 0.003 | 0.029 | PADI4 | 0.036 | 0.009 | <0.001 |
| CD7 | 0.021 | 0.005 | 0.001 | PAG1 | 0.035 | 0.007 | <0.001 |
| CD72 | 0.026 | 0.005 | <0.001 | PAGR1 | 0.014 | 0.005 | 0.040 |
| CD74 | 0.017 | 0.004 | <0.001 | PALM | 0.010 | 0.004 | 0.032 |
| CD79B | 0.014 | 0.005 | 0.041 | PALM2 | 0.015 | 0.005 | 0.013 |
| CD80 | 0.013 | 0.004 | 0.011 | PAPPA | 0.015 | 0.006 | 0.041 |
| CD83 | 0.027 | 0.004 | <0.001 | PARK7 | 0.018 | 0.006 | 0.027 |
| CD93 | 0.016 | 0.004 | <0.001 | PAXX | 0.015 | 0.006 | 0.045 |
| CD99L2 | 0.008 | 0.003 | 0.033 | PBLD | 0.019 | 0.006 | 0.016 |
| CDA | 0.016 | 0.006 | 0.035 | PCBD1 | 0.018 | 0.006 | 0.015 |
| CDCP1 | 0.019 | 0.007 | 0.022 | PCDH7 | 0.012 | 0.004 | 0.028 |
| CDH3 | 0.013 | 0.004 | 0.020 | PCDH9 | 0.011 | 0.004 | 0.040 |
| CDH6 | 0.012 | 0.005 | 0.040 | PCYT2 | 0.024 | 0.009 | 0.044 |
| CDHR5 | 0.029 | 0.005 | <0.001 | PDCD1 | 0.024 | 0.005 | <0.001 |
| CEACAM16 | 0.061 | 0.010 | <0.001 | PDCD1LG2 | 0.010 | 0.004 | 0.032 |
| CEACAM21 | 0.043 | 0.011 | <0.001 | PDCD5 | 0.023 | 0.007 | 0.010 |
| CEACAM6 | 0.029 | 0.005 | <0.001 | PDE1C | -0.018 | 0.007 | 0.039 |
| CEACAM8 | 0.032 | 0.006 | <0.001 | PDGFRA | 0.011 | 0.003 | 0.010 |
| CEBPA | 0.015 | 0.005 | 0.010 | PDZK1 | 0.019 | 0.005 | 0.005 |
| CEBPB | 0.019 | 0.005 | 0.001 | PEBP1 | 0.026 | 0.007 | 0.003 |
| CELA2A | -0.025 | 0.007 | 0.007 | PENK | 0.010 | 0.004 | 0.044 |
| CELA3A | -0.026 | 0.007 | 0.002 | PGF | 0.010 | 0.003 | 0.010 |
| CEP152 | 0.013 | 0.005 | 0.041 | PGLYRP1 | 0.025 | 0.005 | <0.001 |
| CETN2 | 0.024 | 0.008 | 0.024 | PHOSPHO1 | 0.010 | 0.004 | 0.044 |
| CFC1 | 0.019 | 0.006 | 0.007 | PI3 | 0.024 | 0.007 | 0.003 |
| CFD | 0.006 | 0.002 | 0.041 | PIGR | 0.020 | 0.004 | <0.001 |
| CFI | 0.009 | 0.002 | 0.002 | PIK3IP1 | 0.011 | 0.003 | 0.006 |
| CFP | 0.007 | 0.003 | 0.039 | PILRA | 0.017 | 0.005 | 0.008 |
| CHCHD10 | 0.020 | 0.005 | <0.001 | PILRB | 0.019 | 0.007 | 0.034 |
| CHGB | 0.014 | 0.005 | 0.030 | PLA2G15 | 0.011 | 0.003 | 0.007 |
| CHRDL1 | 0.014 | 0.004 | 0.004 | PLA2G7 | 0.012 | 0.004 | 0.011 |
| CHRDL2 | 0.026 | 0.007 | 0.002 | PLAUR | 0.024 | 0.003 | <0.001 |
| CKAP4 | 0.013 | 0.003 | <0.001 | PLTP | -0.017 | 0.004 | 0.001 |
| CLEC14A | 0.014 | 0.004 | 0.002 | PNLIP | -0.024 | 0.008 | 0.012 |
| CLEC4A | -0.011 | 0.004 | 0.036 | POF1B | 0.016 | 0.006 | 0.021 |
| CLEC4D | 0.044 | 0.008 | <0.001 | POLR2F | 0.021 | 0.004 | <0.001 |
| CLEC4G | 0.017 | 0.004 | <0.001 | PON1 | -0.019 | 0.003 | <0.001 |
| CLEC5A | 0.012 | 0.004 | 0.005 | PON2 | 0.017 | 0.004 | <0.001 |
| CLEC6A | 0.019 | 0.006 | 0.008 | PON3 | -0.022 | 0.003 | <0.001 |
| CLEC7A | 0.024 | 0.007 | 0.007 | PPME1 | 0.022 | 0.008 | 0.023 |
| CLMP | 0.010 | 0.003 | 0.003 | PPY | -0.039 | 0.013 | 0.016 |
| CLPP | 0.035 | 0.012 | 0.017 | PQBP1 | 0.019 | 0.007 | 0.030 |
| CNDP1 | -0.030 | 0.006 | <0.001 | PRAP1 | 0.013 | 0.005 | 0.045 |
| CNGB3 | -0.018 | 0.007 | 0.035 | PRCP | 0.010 | 0.004 | 0.039 |
| COL15A1 | 0.008 | 0.003 | 0.041 | PRG2 | 0.024 | 0.006 | 0.002 |
| COL24A1 | 0.027 | 0.007 | 0.001 | PRKAB1 | 0.017 | 0.007 | 0.050 |
| COL6A3 | 0.019 | 0.004 | <0.001 | PRND | 0.047 | 0.006 | <0.001 |
| COLEC12 | 0.013 | 0.003 | <0.001 | PROK1 | 0.024 | 0.008 | 0.018 |
| CPA1 | -0.036 | 0.007 | <0.001 | PRR4 | -0.022 | 0.009 | 0.046 |
| CPB1 | -0.029 | 0.007 | <0.001 | PRSS2 | -0.026 | 0.006 | <0.001 |
| CPM | 0.017 | 0.004 | <0.001 | PRSS27 | 0.018 | 0.005 | 0.003 |
| CPPED1 | 0.022 | 0.008 | 0.041 | PRTN3 | 0.026 | 0.006 | <0.001 |
| CPQ | 0.023 | 0.004 | <0.001 | PSIP1 | 0.031 | 0.008 | 0.001 |
| CPXM2 | 0.014 | 0.003 | <0.001 | PSME2 | 0.014 | 0.005 | 0.013 |
| CR1 | 0.012 | 0.004 | 0.032 | PSMG3 | 0.023 | 0.006 | 0.003 |
| CR2 | 0.021 | 0.006 | 0.002 | PSMG4 | 0.022 | 0.007 | 0.016 |
| CREG1 | 0.021 | 0.005 | <0.001 | PTGDS | 0.016 | 0.004 | <0.001 |
| CRELD1 | 0.014 | 0.005 | 0.014 | PTH | 0.032 | 0.010 | 0.008 |
| CRELD2 | 0.018 | 0.006 | 0.025 | PTK7 | 0.015 | 0.005 | 0.007 |
| CRHBP | 0.009 | 0.003 | 0.038 | PTPRB | 0.009 | 0.004 | 0.049 |
| CRIP2 | 0.024 | 0.005 | <0.001 | PTPRC | 0.008 | 0.003 | 0.022 |
| CRLF1 | 0.012 | 0.003 | 0.002 | PTPRN2 | 0.016 | 0.004 | 0.001 |
| CRTAC1 | -0.010 | 0.004 | 0.040 | PTPRS | 0.019 | 0.003 | <0.001 |
| CRYBB1 | 0.020 | 0.008 | 0.050 | PTS | 0.026 | 0.007 | 0.001 |
| CSF1 | 0.021 | 0.003 | <0.001 | PXN | 0.034 | 0.006 | <0.001 |
| CSF1R | 0.020 | 0.005 | <0.001 | PZP | 0.016 | 0.006 | 0.049 |
| CSF3R | 0.018 | 0.004 | <0.001 | QDPR | 0.019 | 0.005 | 0.003 |
| CST3 | 0.016 | 0.003 | <0.001 | QPCT | 0.014 | 0.003 | <0.001 |
| CST7 | 0.038 | 0.011 | 0.007 | QSOX1 | 0.013 | 0.005 | 0.050 |
| CSTB | 0.025 | 0.005 | <0.001 | RAB44 | 0.020 | 0.005 | 0.002 |
| CTBS | 0.012 | 0.003 | 0.003 | RABGAP1L | 0.023 | 0.008 | 0.018 |
| CTRB1 | -0.023 | 0.007 | 0.007 | RALY | 0.016 | 0.006 | 0.044 |
| CTSH | 0.039 | 0.009 | <0.001 | RARRES2 | 0.023 | 0.006 | 0.001 |
| CTSL | 0.013 | 0.003 | <0.001 | RASSF2 | 0.021 | 0.007 | 0.017 |
| CTSO | 0.017 | 0.004 | <0.001 | RBFOX3 | 0.013 | 0.005 | 0.026 |
| CTSS | 0.015 | 0.003 | <0.001 | RBKS | 0.024 | 0.006 | 0.001 |
| CTSZ | 0.025 | 0.004 | <0.001 | RBP5 | 0.028 | 0.007 | <0.001 |
| CWC15 | 0.021 | 0.008 | 0.029 | RBP7 | 0.032 | 0.008 | <0.001 |
| CX3CL1 | 0.015 | 0.004 | 0.003 | RELT | 0.015 | 0.003 | <0.001 |
| CXCL14 | -0.028 | 0.008 | 0.003 | REN | 0.032 | 0.009 | 0.003 |
| CXCL16 | 0.009 | 0.003 | 0.013 | RET | 0.019 | 0.005 | 0.001 |
| CXCL17 | 0.017 | 0.006 | 0.017 | RETN | 0.026 | 0.005 | <0.001 |
| CYB5R2 | 0.022 | 0.007 | 0.010 | RIDA | 0.026 | 0.006 | <0.001 |
| CYTL1 | 0.014 | 0.003 | <0.001 | RNASE6 | 0.011 | 0.004 | 0.020 |
| DAPK2 | 0.019 | 0.007 | 0.025 | RNASET2 | 0.010 | 0.003 | 0.009 |
| DBI | 0.036 | 0.010 | 0.002 | ROBO1 | 0.010 | 0.003 | 0.004 |
| DDAH1 | 0.024 | 0.005 | <0.001 | ROBO2 | 0.010 | 0.003 | 0.006 |
| DDC | -0.025 | 0.006 | <0.001 | ROR1 | 0.016 | 0.004 | <0.001 |
| DDR1 | 0.009 | 0.003 | 0.008 | RSPO1 | 0.015 | 0.004 | 0.004 |
| DDT | 0.022 | 0.005 | <0.001 | RTN4R | 0.030 | 0.004 | <0.001 |
| DEFA1_DEFA1B | 0.030 | 0.006 | <0.001 | S100A11 | 0.018 | 0.006 | 0.013 |
| DFFA | 0.024 | 0.008 | 0.022 | S100A12 | 0.034 | 0.009 | 0.003 |
| DHPS | 0.014 | 0.005 | 0.025 | S100P | 0.023 | 0.007 | 0.006 |
| DLK1 | 0.021 | 0.007 | 0.014 | SART1 | 0.012 | 0.005 | 0.039 |
| DLL1 | 0.015 | 0.004 | <0.001 | SCARB2 | 0.013 | 0.004 | 0.003 |
| DNAJA2 | 0.028 | 0.010 | 0.027 | SCG2 | 0.010 | 0.004 | 0.040 |
| DNAJB1 | 0.029 | 0.011 | 0.037 | SDC1 | 0.013 | 0.005 | 0.029 |
| DNAJC9 | 0.029 | 0.010 | 0.015 | SDK2 | 0.019 | 0.005 | 0.001 |
| DPEP2 | 0.009 | 0.004 | 0.046 | SELENOP | -0.008 | 0.003 | 0.021 |
| DPP10 | 0.017 | 0.004 | <0.001 | SELL | 0.008 | 0.003 | 0.017 |
| DPY30 | 0.034 | 0.007 | <0.001 | SEMA3F | 0.012 | 0.003 | 0.002 |
| DRAXIN | 0.021 | 0.005 | <0.001 | SEMA4C | 0.010 | 0.003 | 0.021 |
| DSC2 | 0.023 | 0.004 | <0.001 | SEPTIN8 | 0.013 | 0.004 | 0.014 |
| DSCAM | 0.011 | 0.004 | 0.039 | SEPTIN9 | 0.009 | 0.004 | 0.046 |
| DTNB | 0.015 | 0.006 | 0.033 | SERPINA1 | 0.003 | 0.001 | 0.030 |
| DYNLT1 | 0.036 | 0.010 | 0.002 | SERPINA7 | 0.013 | 0.002 | <0.001 |
| EFCAB14 | 0.009 | 0.003 | 0.039 | SERPINB5 | 0.018 | 0.006 | 0.017 |
| EFNA1 | 0.016 | 0.003 | <0.001 | SERPINB6 | 0.020 | 0.007 | 0.030 |
| EFNA4 | 0.022 | 0.004 | <0.001 | SERPINB8 | 0.028 | 0.007 | 0.001 |
| EGLN1 | 0.026 | 0.007 | 0.001 | SERPINC1 | 0.005 | 0.002 | 0.045 |
| EIF4B | 0.020 | 0.007 | 0.022 | SERPIND1 | 0.013 | 0.003 | 0.002 |
| EIF4G1 | 0.033 | 0.012 | 0.037 | SERPINF1 | 0.013 | 0.003 | 0.001 |
| ELOA | 0.025 | 0.007 | 0.002 | SERPINF2 | 0.006 | 0.002 | 0.003 |
| ENAH | 0.015 | 0.006 | 0.047 | SETMAR | 0.014 | 0.004 | 0.001 |
| ENDOU | 0.020 | 0.007 | 0.018 | SF3B4 | 0.037 | 0.009 | <0.001 |
| ENPP2 | 0.008 | 0.003 | 0.048 | SFRP1 | 0.014 | 0.005 | 0.038 |
| ENPP5 | -0.017 | 0.005 | 0.008 | SFRP4 | 0.025 | 0.005 | <0.001 |
| EPHA1 | 0.018 | 0.004 | <0.001 | SFTPA2 | 0.026 | 0.006 | <0.001 |
| EPHA2 | 0.014 | 0.004 | 0.002 | SHISA5 | 0.013 | 0.003 | <0.001 |
| EPHA4 | 0.011 | 0.003 | 0.011 | SIGLEC1 | 0.020 | 0.005 | 0.001 |
| EPHB4 | 0.012 | 0.003 | <0.001 | SIGLEC10 | 0.015 | 0.004 | 0.003 |
| EPHB6 | 0.021 | 0.004 | <0.001 | SIGLEC6 | 0.016 | 0.004 | 0.001 |
| EPS8L2 | 0.013 | 0.004 | 0.004 | SIGLEC8 | 0.019 | 0.006 | 0.005 |
| ERMAP | 0.023 | 0.006 | <0.001 | SIRPB1 | 0.017 | 0.006 | 0.015 |
| ESAM | 0.010 | 0.004 | 0.046 | SIRT2 | 0.032 | 0.011 | 0.021 |
| EZR | 0.011 | 0.003 | 0.002 | SIT1 | 0.027 | 0.007 | <0.001 |
| F7 | 0.016 | 0.004 | <0.001 | SKAP1 | 0.029 | 0.009 | 0.010 |
| FABP1 | 0.064 | 0.012 | <0.001 | SLAMF7 | -0.019 | 0.007 | 0.036 |
| FABP3 | 0.030 | 0.006 | <0.001 | SLC27A4 | 0.021 | 0.008 | 0.047 |
| FABP4 | 0.030 | 0.006 | <0.001 | SLITRK1 | 0.012 | 0.004 | 0.017 |
| FABP5 | 0.037 | 0.008 | <0.001 | SLITRK6 | 0.011 | 0.004 | 0.036 |
| FAM3C | 0.016 | 0.004 | <0.001 | SMAD1 | 0.027 | 0.010 | 0.036 |
| FAS | 0.011 | 0.004 | 0.049 | SMNDC1 | 0.027 | 0.008 | 0.003 |
| FASLG | -0.014 | 0.005 | 0.018 | SMOC1 | 0.016 | 0.004 | 0.001 |
| FBLN2 | 0.014 | 0.004 | 0.003 | SMPD1 | 0.018 | 0.006 | 0.021 |
| FCAMR | 0.058 | 0.009 | <0.001 | SNRPB2 | 0.021 | 0.005 | 0.001 |
| FCAR | 0.018 | 0.006 | 0.014 | SOD1 | 0.022 | 0.006 | 0.003 |
| FCER2 | 0.030 | 0.006 | <0.001 | SORCS2 | 0.019 | 0.004 | <0.001 |
| FCGR2B | 0.027 | 0.007 | 0.001 | SPINK4 | 0.026 | 0.008 | 0.009 |
| FCRL1 | 0.022 | 0.006 | 0.001 | SPINK5 | 0.013 | 0.004 | 0.016 |
| FETUB | 0.022 | 0.004 | <0.001 | SPINT1 | 0.013 | 0.003 | <0.001 |
| FGF19 | -0.030 | 0.010 | 0.011 | SPOCK1 | -0.008 | 0.003 | 0.037 |
| FGFBP3 | 0.012 | 0.005 | 0.036 | SPON1 | 0.014 | 0.004 | 0.003 |
| FGFR2 | 0.011 | 0.003 | <0.001 | SPRR3 | 0.031 | 0.008 | 0.002 |
| FGFR4 | 0.019 | 0.007 | 0.021 | SRP14 | 0.030 | 0.008 | <0.001 |
| FGR | 0.033 | 0.007 | <0.001 | SSC4D | 0.090 | 0.014 | <0.001 |
| FHIT | 0.026 | 0.009 | 0.025 | SSNA1 | 0.016 | 0.006 | 0.042 |
| FKBP4 | 0.017 | 0.005 | 0.010 | ST3GAL1 | -0.017 | 0.005 | 0.010 |
| FLI1 | 0.036 | 0.014 | 0.040 | STAB2 | 0.010 | 0.003 | 0.030 |
| FLRT2 | 0.017 | 0.003 | <0.001 | STAMBP | 0.021 | 0.008 | 0.044 |
| FLT1 | 0.008 | 0.003 | 0.020 | STC2 | 0.012 | 0.003 | 0.003 |
| FLT3 | 0.010 | 0.004 | 0.032 | STIP1 | 0.024 | 0.008 | 0.020 |
| FMNL1 | 0.031 | 0.008 | <0.001 | STK11 | 0.025 | 0.009 | 0.027 |
| FN1 | 0.010 | 0.003 | 0.018 | STX16 | 0.019 | 0.007 | 0.038 |
| FNDC1 | 0.023 | 0.006 | <0.001 | STX6 | 0.027 | 0.008 | 0.008 |
| FNTA | 0.013 | 0.004 | 0.016 | STX8 | 0.019 | 0.006 | 0.010 |
| FOLR1 | 0.012 | 0.003 | 0.003 | SUGT1 | 0.026 | 0.010 | 0.044 |
| FOLR2 | 0.018 | 0.004 | <0.001 | SULT2A1 | 0.019 | 0.007 | 0.031 |
| FSTL1 | 0.007 | 0.003 | 0.043 | SUMF2 | 0.019 | 0.006 | 0.018 |
| FSTL3 | 0.014 | 0.004 | 0.003 | SUSD4 | 0.019 | 0.005 | 0.003 |
| FUOM | 0.025 | 0.008 | 0.010 | SWAP70 | 0.018 | 0.004 | <0.001 |
| FUS | 0.016 | 0.005 | 0.007 | TAFA5 | 0.022 | 0.004 | <0.001 |
| FXN | 0.023 | 0.009 | 0.049 | TALDO1 | 0.026 | 0.006 | <0.001 |
| GALNT3 | 0.020 | 0.005 | 0.002 | TCL1A | 0.052 | 0.012 | <0.001 |
| GCHFR | 0.024 | 0.006 | 0.001 | TCOF1 | 0.015 | 0.004 | <0.001 |
| GDF15 | 0.030 | 0.005 | <0.001 | TDP1 | 0.028 | 0.009 | 0.009 |
| GDNF | 0.014 | 0.005 | 0.014 | TEK | 0.007 | 0.003 | 0.043 |
| GET3 | 0.017 | 0.007 | 0.046 | TFF3 | 0.023 | 0.006 | <0.001 |
| GFER | 0.023 | 0.008 | 0.021 | TFPI2 | 0.017 | 0.005 | 0.003 |
| GFRA1 | 0.020 | 0.003 | <0.001 | TG | 0.058 | 0.014 | <0.001 |
| GFRA2 | 0.010 | 0.003 | <0.001 | TGFA | 0.032 | 0.006 | <0.001 |
| GGCT | 0.016 | 0.006 | 0.041 | TGFBR2 | 0.019 | 0.004 | <0.001 |
| GGH | 0.029 | 0.004 | <0.001 | THBD | 0.009 | 0.003 | 0.039 |
| GGT5 | 0.010 | 0.003 | 0.002 | THBS4 | 0.019 | 0.006 | 0.007 |
| GIMAP7 | -0.012 | 0.004 | 0.015 | THY1 | 0.019 | 0.003 | <0.001 |
| GIPC2 | 0.011 | 0.004 | 0.018 | TIMD4 | 0.015 | 0.005 | 0.028 |
| GLB1 | 0.019 | 0.005 | 0.002 | TMPRSS11B | -0.028 | 0.010 | 0.020 |
| GLOD4 | 0.013 | 0.004 | 0.019 | TMPRSS5 | 0.013 | 0.005 | 0.040 |
| GMPR | 0.016 | 0.006 | 0.050 | TMSB10 | 0.038 | 0.009 | <0.001 |
| GPC1 | 0.014 | 0.004 | 0.002 | TNFRSF10A | 0.011 | 0.004 | 0.025 |
| GPKOW | 0.014 | 0.004 | <0.001 | TNFRSF10B | 0.015 | 0.005 | 0.015 |
| GPR37 | 0.025 | 0.008 | 0.012 | TNFRSF10C | 0.021 | 0.006 | 0.005 |
| GRIK2 | -0.014 | 0.005 | 0.034 | TNFRSF11A | 0.027 | 0.005 | <0.001 |
| GRPEL1 | 0.022 | 0.007 | 0.016 | TNFRSF13B | 0.011 | 0.004 | 0.041 |
| GSTA1 | 0.050 | 0.010 | <0.001 | TNFRSF14 | 0.019 | 0.004 | <0.001 |
| GSTA3 | 0.043 | 0.009 | <0.001 | TNFRSF17 | -0.015 | 0.005 | 0.015 |
| GUSB | 0.044 | 0.007 | <0.001 | TNFRSF19 | 0.015 | 0.004 | <0.001 |
| H2AP | 0.016 | 0.006 | 0.044 | TNFRSF1A | 0.019 | 0.004 | <0.001 |
| HAVCR2 | 0.023 | 0.004 | <0.001 | TNFRSF1B | 0.018 | 0.004 | <0.001 |
| HCLS1 | 0.019 | 0.007 | 0.025 | TNFRSF21 | 0.012 | 0.003 | 0.002 |
| HDGFL2 | 0.030 | 0.008 | 0.002 | TNFRSF4 | 0.026 | 0.005 | <0.001 |
| HGF | 0.030 | 0.004 | <0.001 | TNFRSF9 | 0.015 | 0.005 | 0.019 |
| HGS | 0.026 | 0.010 | 0.034 | TNFSF13 | 0.010 | 0.003 | 0.016 |
| HHEX | 0.026 | 0.009 | 0.032 | TNFSF13B | 0.018 | 0.003 | <0.001 |
| HIF1A | 0.022 | 0.008 | 0.032 | TNFSF14 | 0.025 | 0.007 | 0.004 |
| HIP1R | 0.016 | 0.005 | 0.012 | TNFSF8 | 0.020 | 0.004 | <0.001 |
| HJV | 0.016 | 0.005 | 0.013 | TNN | 0.014 | 0.005 | 0.040 |
| HMOX1 | -0.018 | 0.006 | 0.014 | TOP2B | 0.031 | 0.010 | 0.012 |
| HNMT | 0.033 | 0.006 | <0.001 | TOR1AIP1 | 0.019 | 0.006 | 0.016 |
| HNRNPK | 0.025 | 0.010 | 0.041 | TP53 | 0.014 | 0.005 | 0.045 |
| HNRNPUL1 | 0.023 | 0.006 | 0.001 | TP53I3 | 0.021 | 0.007 | 0.012 |
| HRG | 0.010 | 0.004 | 0.039 | TPK1 | 0.018 | 0.003 | <0.001 |
| HS6ST2 | 0.030 | 0.004 | <0.001 | TPR | 0.020 | 0.006 | 0.007 |
| HSD11B1 | -0.014 | 0.005 | 0.027 | TREM2 | 0.028 | 0.007 | <0.001 |
| HSPA1A | 0.023 | 0.009 | 0.046 | TREML2 | 0.022 | 0.004 | <0.001 |
| HSPA2 | 0.012 | 0.004 | 0.030 | TRIAP1 | 0.026 | 0.008 | 0.006 |
| HSPB6 | 0.015 | 0.005 | 0.014 | TSNAX | 0.015 | 0.005 | 0.018 |
| ICAM1 | 0.020 | 0.004 | <0.001 | TSPYL1 | 0.015 | 0.005 | 0.021 |
| ICOSLG | 0.011 | 0.002 | <0.001 | TXNDC9 | 0.016 | 0.006 | 0.048 |
| IDI2 | 0.017 | 0.006 | 0.020 | TXNRD1 | 0.017 | 0.006 | 0.021 |
| IDUA | 0.032 | 0.006 | <0.001 | ULBP2 | 0.020 | 0.005 | <0.001 |
| IFI30 | 0.025 | 0.005 | <0.001 | UPB1 | 0.048 | 0.011 | <0.001 |
| IFNG | -0.051 | 0.014 | 0.003 | USO1 | 0.029 | 0.011 | 0.044 |
| IFNGR1 | 0.010 | 0.003 | <0.001 | USP8 | 0.032 | 0.012 | 0.034 |
| IFNL1 | 0.018 | 0.005 | 0.006 | VASN | 0.011 | 0.003 | 0.007 |
| IFNLR1 | 0.015 | 0.005 | 0.007 | VAT1 | 0.007 | 0.002 | 0.021 |
| IGDCC4 | 0.011 | 0.003 | 0.007 | VCAM1 | 0.010 | 0.003 | 0.012 |
| IGFBP4 | 0.015 | 0.005 | 0.018 | VEGFD | 0.015 | 0.004 | 0.005 |
| IGFBP6 | 0.013 | 0.004 | 0.003 | VNN2 | 0.026 | 0.006 | <0.001 |
| IGSF8 | 0.012 | 0.004 | 0.007 | VSIG10 | 0.011 | 0.004 | 0.041 |
| IL10RB | 0.016 | 0.003 | <0.001 | VSIG4 | 0.018 | 0.005 | 0.003 |
| IL16 | 0.024 | 0.005 | <0.001 | VSTM1 | 0.026 | 0.006 | <0.001 |
| IL18 | 0.022 | 0.006 | 0.002 | VWC2 | 0.027 | 0.005 | <0.001 |
| IL18BP | 0.014 | 0.004 | 0.001 | WFDC12 | -0.020 | 0.008 | 0.047 |
| IL18R1 | 0.026 | 0.004 | <0.001 | WFDC2 | 0.018 | 0.005 | 0.002 |
| IL1R1 | 0.008 | 0.003 | 0.024 | WFIKKN1 | 0.022 | 0.005 | <0.001 |
| IL1RL2 | 0.020 | 0.005 | <0.001 | WWP2 | 0.024 | 0.007 | 0.003 |
| IL1RN | 0.040 | 0.007 | <0.001 | XRCC4 | 0.014 | 0.005 | 0.017 |
| IL2RA | 0.013 | 0.005 | 0.034 | YAP1 | 0.017 | 0.003 | <0.001 |
| IL31RA | -0.017 | 0.005 | 0.005 | ZBTB17 | 0.014 | 0.005 | 0.028 |
| IL36G | 0.024 | 0.008 | 0.026 | ZHX2 | 0.013 | 0.004 | 0.013 |
| IL6 | 0.026 | 0.009 | 0.023 | C3 | 0.016 | 0.006 | 0.024 |
| IL7R | -0.018 | 0.006 | 0.029 | CFH | 0.011 | 0.003 | 0.002 |
| INHBB | 0.022 | 0.006 | 0.004 | GM2A | 0.017 | 0.005 | 0.003 |
| INHBC | 0.024 | 0.005 | <0.001 | NPC2 | 0.021 | 0.004 | <0.001 |
| INSR | 0.007 | 0.002 | 0.008 | PSAP | 0.016 | 0.004 | <0.001 |
| ISM1 | 0.017 | 0.005 | 0.004 |  |  |  |  |

Abbreviation: FDR, false discovery rate; SE, standard error.

**Supplementary Table S12.** FDR-significant circulating proteins associated with loneliness.

| **Proteins** | **Estimate** | **SE** | **P for FDR** |
| --- | --- | --- | --- |
| AMBP | 0.026 | 0.007 | 0.025 |
| AREG | 0.054 | 0.015 | 0.042 |
| CCL16 | 0.069 | 0.017 | 0.021 |
| CCL21 | 0.049 | 0.014 | 0.038 |
| CD59 | 0.027 | 0.007 | 0.038 |
| CLEC4D | 0.105 | 0.021 | 0.000 |
| COL18A1 | 0.029 | 0.008 | 0.033 |
| COL6A3 | 0.048 | 0.011 | 0.008 |
| CST3 | 0.034 | 0.009 | 0.038 |
| EPHB4 | 0.028 | 0.008 | 0.045 |
| FABP1 | 0.111 | 0.032 | 0.046 |
| FABP3 | 0.062 | 0.017 | 0.038 |
| FABP4 | 0.092 | 0.017 | 0.000 |
| FGF21 | 0.154 | 0.040 | 0.023 |
| FGF23 | 0.074 | 0.019 | 0.021 |
| FGFR2 | 0.030 | 0.007 | 0.016 |
| GFRA1 | 0.034 | 0.009 | 0.038 |
| GSAP | 0.102 | 0.029 | 0.046 |
| IFNGR1 | 0.025 | 0.007 | 0.038 |
| IGSF9 | 0.099 | 0.028 | 0.038 |
| IL1RN | 0.100 | 0.018 | <0.001 |
| LGALS1 | 0.042 | 0.012 | 0.038 |
| LGALS3 | 0.038 | 0.010 | 0.028 |
| LGALS4 | 0.065 | 0.018 | 0.038 |
| NELL1 | -0.061 | 0.014 | 0.006 |
| OCLN | 0.060 | 0.015 | 0.016 |
| PLAUR | 0.034 | 0.009 | 0.028 |
| PRSS8 | 0.064 | 0.014 | 0.002 |
| PVR | 0.048 | 0.012 | 0.022 |
| THY1 | 0.033 | 0.009 | 0.038 |
| TNFRSF11A | 0.059 | 0.013 | 0.002 |
| TNFRSF1A | 0.038 | 0.010 | 0.021 |

Abbreviation: FDR, false discovery rate; SE, standard error.

Supplementary Table S13. Association between social isolation, loneliness, and risk of IBD stratified by sex.

| **Sex** | **Subgroup** | **HR (95% CI)** | **P value** |  |
| --- | --- | --- | --- | --- |
| **Male** | **Social isolation** |  |  | |
|  | Least isolated | 1.00 [Reference] |  | |
|  | Moderately isolated | 1.13 (0.98-1.31) | 0.102 | |
|  | Most isolated | 1.40 (1.02-1.91) | 0.037 | |
|  | **P trend** | 0.017 | | |
|  | **Loneliness** |  |  | |
|  | No loneliness | 1.00 [Reference] |  | |
|  | Loneliness | 1.39 (1.06-1.82) | 0.019 | |
| **Female** | **Social isolation** |  |  | |
|  | Least isolated | 1.00 [Reference] |  | |
|  | Moderately isolated | 1.12 (0.96-1.32) | 0.161 | |
|  | Most isolated | 1.17 (0.74-1.86) | 0.496 | |
|  | **P trend** | 0.142 | | |
|  | **Loneliness** |  |  | |
|  | No loneliness | 1.00 [Reference] |  | |
|  | Loneliness | 1.17 (0.82-1.65) | 0.384 | |

The HR represents the results after adjusting for Model (age, ethnicity, BMI, household income, education level, employment status, smoking status, drinking status, physical activity, healthy diet pattern, and healthy sleep pattern, and PRS for IBD). Abbreviation: BMI, body mass index; HR, hazard ratio; IBD, inflammatory bowel disease; PRS, polygenic risk scores.

Supplementary Table S14. Impact of social isolation and loneliness on the risk of IBD among European populations.

| **Subgroup** | **HR (95% CI)** | **P value** |
| --- | --- | --- |
| **Social isolation** |  |  |
| Least isolated | 1.00 [Reference] |  |
| Moderately isolated | 1.13 (1.01-1.26) | 0.039 |
| Most isolated | 1.30 (0.98-1.71) | 0.066 |
| **P trend** | 0.006 | |
| **Loneliness** |  |  |
| No loneliness | 1.00 [Reference] |  |
| Loneliness | 1.29 (1.03-1.61) | 0.024 |

The HR represents the results after adjusting for Model (age, sex, BMI, household income, education level, employment status, smoking status, drinking status, physical activity, healthy diet pattern, and healthy sleep pattern, and PRS for IBD). Abbreviation: BMI, body mass index; HR, hazard ratio; IBD, inflammatory bowel disease; PRS, polygenic risk scores.

Supplementary Table S15. Association between social isolation, loneliness, and IBD risk with additional adjustment for the Charlson Comorbidity Index.

| **Subgroup** | **HR (95% CI)** | **P value** |
| --- | --- | --- |
| **Social isolation** |  |  |
| Least isolated | 1.00 [Reference] |  |
| Moderately isolated | 1.12 (1.01-1.25) | 0.035 |
| Most isolated | 1.30 (1.00-1.68) | 0.047 |
| **P trend** |  | 0.003 |
| **Loneliness** |  |  |
| No loneliness | 1.00 [Reference] |  |
| Loneliness | 1.27 (1.03-1.57) | 0.028 |

The HR represents the results after adjusting for Model (age, sex, ethnicity, BMI, household income, education level, employment status, smoking status, drinking status, physical activity, healthy diet pattern, and healthy sleep pattern, PRS for IBD and CCI).

Abbreviation: BMI, body mass index; CCI, Charlson Comorbidity Index; HR, hazard ratio; IBD, inflammatory bowel disease; PRS, polygenic risk scores.

**Supplementary Table S16.** Associations of social isolation and loneliness with IBD risk with all incident cases retained.

| **Exposure** | **Model 1 HR**  **(95% CI)** | **Model 2 HR**  **(95% CI)** | **Model 3 HR**  **(95% CI)** | **P value for**  **Model 1** | **P value for**  **Model 2** | **P value for**  **Model 3** |
| --- | --- | --- | --- | --- | --- | --- |
| **Separate effects** |  |  |  |  |  |  |
| Social isolation |  |  |  |  |  |  |
| Least isolated | 1.00 [Reference] | 1.00 [Reference] | 1.00 [Reference] |  |  |  |
| Moderately isolated | 1.2 (1.09-1.34) | 1.16 (1.04-1.29) | 1.16 (1.04-1.28) | < 0.001 | 0.006 | 0.007 |
| Most isolated | 1.38 (1.08-1.78) | 1.31 (1.02-1.68) | 1.31 (1.02-1.68) | 0.011 | 0.037 | 0.038 |
| **P trend** | < 0.001 | 0.057 | 0.057 |  |  |  |
| Loneliness |  |  |  |  |  |  |
| No loneliness | 1.00 [Reference] | 1.00 [Reference] | 1.00 [Reference] |  |  |  |
| Loneliness | 1.44 (1.17-1.78) | 1.25 (1.01-1.55) | 1.24 (1.01-1.54) | 0.001 | 0.036 | 0.043 |
| **Joint effects** |  |  |  |  |  |  |
| **No loneliness** |  |  |  |  |  |  |
| Least isolated | 1.00 [Reference] | 1.00 [Reference] | 1.00 [Reference] |  |  |  |
| Moderately isolated | 1.18 (1.06-1.31) | 1.14 (1.02-1.27) | 1.14 (1.02-1.27) | 0.002 | 0.016 | 0.018 |
| Most isolated | 1.27 (0.97-1.68) | 1.23 (0.93-1.62) | 1.23 (0.93-1.62) | 0.086 | 0.149 | 0.152 |
| **Loneliness** |  |  |  |  |  |  |
| Least isolated | 1.2 (0.87-1.66) | 1.07 (0.77-1.48) | 1.06 (0.77-1.47) | 0.265 | 0.695 | 0.721 |
| Moderately isolated | 1.8 (1.33-2.44) | 1.51 (1.11-2.06) | 1.49 (1.1-2.03) | <0.001 | 0.009 | 0.011 |
| Most isolated | 2.36 (1.33-4.17) | 1.91 (1.08-3.39) | 1.92 (1.08-3.4) | 0.003 | 0.026 | 0.025 |
| **P trend** | < 0.001 | < 0.001 | < 0.001 |  |  |  |

Model 1 adjusted for age, sex, and ethnicity; Model 2 further adjusted for BMI, household income, educational level, employment status, smoking status, alcohol consumption, physical activity, TDI, healthy diet pattern, and healthy sleep pattern; Model 3 additionally adjusted for PRS of IBD. Abbreviation: BMI, body mass index; HR, hazard ratio; IBD, inflammatory bowel disease; PAF, population attributable fraction; PRS, polygenic risk score.

**Supplementary Table S17.** Mediation analysis of circulating metabolites associated with social isolation and loneliness.

| **Exposure** | **Metabolites** | **ACME** | **P value** | **Proportion** | **P value** |
| --- | --- | --- | --- | --- | --- |
| **Social isolation** |  |  |  |  |  |
|  | HDL cholesterol | -42.11 (-115.04, 10.42) | 0.128 | 4.40 (-75.20, 89.76) | 0.438 |
|  | Cholesteryl esters in HDL | -37.73 (-103.31, 13.78) | 0.150 | 3.91 (-49.97, 83.20) | 0.432 |
|  | Average diameter for HDL particles | -10.27 (-65.00, 43.06) | 0.708 | 0.81 (-39.66, 39.50) | 0.818 |
|  | Degree of unsaturation | -28.83 (-72.64, 5.58) | 0.096 | 2.99 (-41.07, 63.22) | 0.414 |
|  | Docosahexaenoic acid | -52.65 (-119.06, -6.79) | 0.026 | 5.84 (-76.95, 71.81) | 0.346 |
|  | Lactate | -12.31 (-46.47, 20.26) | 0.404 | 1.35 (-26.50, 33.76) | 0.590 |
|  | Glycoprotein acetyls | -31.33 (-71.77, -4.94) | 0.012 | 3.23 (-72.07, 47.49) | 0.402 |
|  | Cholesterol in medium HDL | -23.09 (-81.80, 29.44) | 0.384 | 2.29 (-29.46, 37.18) | 0.59 |
|  | Metabolite feature score | -19.05 (-63.53, 19.6) | 0.33 | 2.05 (-20.97, 33.74) | 0.524 |
| **Loneliness** | Degree of unsaturation | -58.20 (-160.72, 7.85) | 0.098 | 1.87 (-65.83, 74.58) | 0.814 |
|  | Omega-3 fatty acids | -45.79 (-116.00, -0.33) | 0.050 | 1.84 (-45.68, 48.46) | 0.780 |
|  | Docosahexaenoic acid | -67.48 (-158.82, -11.10) | 0.012 | 2.93 (-57.74, 77.38) | 0.762 |
|  | Acetate | -12.14 (-72.84, 40.80) | 0.652 | 0.10 (-34.19, 28.17) | 0.954 |
|  | Albumin | -88.48 (-192.28, -27.73) | <0.001 | 3.72 (-112.38, 109.78) | 0.810 |
|  | Metabolite feature score | -7.91 (-32.2, 12.32) | 0.386 | 0.74 (-13.33, 20.92) | 0.578 |

Abbreviation: ACME, average causal mediation effect; HDL, high-density lipoprotein.

**Supplementary Table S18.** Mediation analysis of circulating proteins associated with social isolation and loneliness.

| **Exposure** | **Proteins** | **ACME (95% CI)** | **P value** | **Proportion (95% CI)** | **P value** |
| --- | --- | --- | --- | --- | --- |
| **Social isolation** | AMBP | -63.02 (-152.42, -13.94) | 0.002 | 2.75 (-82.19, 115.41) | 0.904 |
|  | AREG | -56.60 (-139.65, -10.60) | 0.008 | 2.52 (-109.76, 122.94) | 0.924 |
|  | CCL21 | -20.48 (-65.59, 1.15) | 0.072 | -0.26 (-45.36, 34.55) | 0.960 |
|  | CD59 | -16.40 (-53.90, 1.50) | 0.108 | 0.60 (-26.74, 22.08) | 0.812 |
|  | CLEC4D | -94.39 (-204.32, -24.59) | <0.001 | 4.13 (-200.14, 185.56) | 0.950 |
|  | COL6A3 | -19.07 (-67.93, 17.74) | 0.322 | 0.83 (-52.35, 49.16) | 0.810 |
|  | CST3 | -53.50 (-139.69, -7.43) | 0.010 | -1.30 (-127.01, 67.90) | 0.978 |
|  | EPHB4 | -29.58 (-85.35, 9.28) | 0.146 | 1.77 (-52.36, 66.85) | 0.754 |
|  | FABP1 | -26.45 (-96.97, 23.25) | 0.312 | -0.64 (-53.05, 52.14) | 0.912 |
|  | FABP3 | -10.24 (-61.63, 30.65) | 0.616 | -0.33 (-29.38, 27.16) | 0.886 |
|  | FABP4 | -38.14 (-116.87, 9.65) | 0.134 | 2.63 (-61.94, 62.26) | 0.712 |
|  | FGFR2 | -29.09 (-89.08, 6.21) | 0.140 | -0.46 (-51.84, 46.62) | 0.948 |
|  | GFRA1 | -70.63 (-168.03, -14.11) | 0.006 | 3.21 (-169.60, 166.47) | 0.902 |
|  | IFNGR1 | -45.88 (-118.07, -4.23) | 0.016 | -1.72 (-96.48, 89.10) | 0.924 |
|  | IL1RN | -80.51 (-187.73, -21.32) | <0.001 | -2.80 (-165.15, 170.33) | 0.966 |
|  | LGALS1 | -52.62 (-133.25, -4.41) | 0.034 | 3.70 (-116.07, 118.92) | 0.768 |
|  | LGALS4 | -46.08 (-126.83, -5.75) | 0.006 | -1.88 (-107.21, 68.48) | 0.888 |
|  | OCLN | -68.38 (-184.86, -13.81) | 0.002 | -3.23 (-91.59, 101.85) | 0.740 |
|  | PLAUR | -164.08 (-351.32, -54.80) | <0.001 | -9.97 (-251.99, 281.05) | 0.860 |
|  | THY1 | -16.03 (-81.59, 39.64) | 0.532 | 0.42 (-42.51, 40.23) | 0.906 |
|  | TNFRSF11A | -93.06 (-220.02, -25.72) | 0.004 | -3.03 (-117.47, 162.10) | 0.970 |
|  | TNFRSF1A | -81.46 (-188.69, -21.61) | <0.001 | -3.24 (-164.12, 169.23) | 0.970 |
|  | Protein score | -108.23 (-247.36, -25.47) | <0.001 | -6.26 (-208.08, 132.89) | 0.806 |
| **Loneliness** | AMBP | -145.55 (-360.74, -28.74) | 0.002 | -2.59 (-95.84, 93.44) | 0.916 |
|  | AREG | -118.09 (-310.88, -19.79) | 0.006 | -2.02 (-64.13, 91.76) | 0.914 |
|  | CCL21 | -66.67 (-198.00, 5.01) | 0.082 | -0.82 (-58.20, 37.98) | 0.918 |
|  | CD59 | -67.61 (-204.40, 2.87) | 0.060 | 0.29 (-48.69, 77.48) | 0.978 |
|  | CLEC4D | -211.39 (-499.38, -55.20) | <0.001 | -5.26 (-167.08, 147.87) | 0.906 |
|  | COL6A3 | -51.95 (-190.49, 39.42) | 0.270 | -0.53 (-44.89, 51.97) | 0.912 |
|  | CST3 | -99.71 (-267.88, -12.87) | 0.016 | -0.43 (-89.94, 66.10) | 0.990 |
|  | EPHB4 | -58.39 (-168.09, 13.53) | 0.136 | -0.48 (-50.93, 33.82) | 0.938 |
|  | FABP1 | -43.72 (-169.00, 37.91) | 0.308 | -0.31 (-31.37, 43.96) | 0.922 |
|  | FABP3 | -31.93 (-202.70, 107.15) | 0.646 | -0.45 (-18.92, 12.97) | 0.780 |
|  | FABP4 | -90.17 (-256.52, 23.80) | 0.130 | -1.19 (-84.47, 93.43) | 0.920 |
|  | FGFR2 | -76.08 (-241.28, 18.24) | 0.104 | -0.83 (-62.52, 70.57) | 0.918 |
|  | GFRA1 | -108.89 (-262.14, -14.59) | 0.016 | -2.24 (-66.15, 84.18) | 0.864 |
|  | IFNGR1 | -107.03 (-284.60, -15.84) | 0.018 | -1.58 (-61.70, 86.93) | 0.924 |
|  | IL1RN | -179.74 (-427.88, -37.48) | <0.001 | -4.36 (-101.52, 135.18) | 0.856 |
|  | LGALS1 | -98.04 (-264.88, -5.94) | 0.028 | -1.54 (-77.85, 82.96) | 0.918 |
|  | LGALS4 | -126.78 (-331.15, -23.00) | 0.002 | -2.08 (-80.94, 61.87) | 0.894 |
|  | OCLN | -270.66 (-760.96, -38.78) | 0.004 | -3.35 (-31.99, 35.31) | 0.224 |
|  | PLAUR | -224.09 (-533.78, -58.62) | <0.001 | -3.26 (-149.40, 151.84) | 0.962 |
|  | THY1 | -23.97 (-143.56, 70.29) | 0.594 | -0.10 (-27.26, 28.46) | 0.958 |
|  | TNFRSF11A | -171.82 (-421.90, -38.23) | 0.004 | -3.26 (-164.97, 103.81) | 0.940 |
|  | TNFRSF1A | -148.06 (-365.04, -38.43) | <0.001 | -3.13 (-100.33, 92.59) | 0.922 |
|  | Protein score | -117.9 (-261.83, -32.15) | <0.001 | -6.98 (-213.63, 176.37) | 0.798 |

Abbreviation: ACME, average causal mediation effect.

**Supplementary Table S19.** Mediation analysis of modifiable lifestyle factors in the associations between social isolation, loneliness, and IBD risk.

| **Mediators** | **Social isolation** | | | | **Loneliness** | |
| --- | --- | --- | --- | --- | --- | --- |
|  | **ACME (95% CI)** | **Percentage mediated (95% CI)** | **P value** | **ACME (95% CI)** | **Percentage mediated (95% CI)** | **P value** |
| **BMI** | -36.96 (-61.32, -18.04) | 5.83 (2.16, 25.05) | 0.022 | -99.35 (-161.87, -42.32) | 8.07 (2.33, 39.25) | 0.024 |
| **Currently smoking** | -50.48 (-74.57, -29.88) | 7.74 (3.46, 37.33) | 0.022 | -102.15 (-148.12, -61.84) | 8.19 (3.76, 29.99) | 0.024 |
| **Physical activity** | -9.16 (-72.57, 49.09) | 1.50 (-15.12, 19.43) | 0.786 | -7.46 (-35.18, 19.35) | 0.64 (-2.76, 6.26) | 0.586 |
| **Healthy diet pattern** | -2.79 (-15.26, 7.49) | 0.46 (-2.05, 4.73) | 0.61 | -5.27 (-28.15, 14.43) | 0.45 (-2.19, 3.97) | 0.620 |
| **Healthy sleep pattern** | -11.95 (-28.13, 2.56) | 1.95 (-0.7, 12.3) | 0.122 | -60.49 (-142.27, 17.93) | 5.00 (-1.51, 20.36) | 0.142 |

Abbreviation: ACME, average causal mediation effect; BMI, body mass index.

**Supplementary Table S20.** MR analysis for association between social isolation/loneliness and IBD, MR-Egger intercept test for horizontal pleiotropy, and heterogeneity tests with Cochran’s Q statistic.

| **Exposure** |  | **No. of SNPs** | **Random-effects IVW Estimates** | | **MR-Egger Tests** | | | **MR-PRESSO global test** | **Heterogeneity Tests** | | |
| --- | --- | --- | --- | --- | --- | --- | --- | --- | --- | --- | --- |
|  |  |  | **OR (95% CI)** | **P value** | **Intercept** | **std. error** | **P value** | **P value** | **Q value** | **df** | **P value** |
| ukb-b-8476 | Feeling lonely | 79 | 0.82 (0.46-1.44) | 0.484 | -8.97E-04 | 6.96E-03 | 0.898 | 0.985 | 54.596 | 78 | 0.980 |
| ukb-b-5445 | Not living alone | 20 | 0.93 (0.49-1.76) | 0.829 | -1.06E-02 | 9.74E-03 | 0.291 | 0.823 | 12.858 | 19 | 0.846 |
| ukb-b-5379 | More contact with friends or family | 102 | 0.87 (0.72-1.06) | 0.181 | -1.08E-02 | 4.76E-03 | 0.026 | 0.711 | 93.475 | 101 | 0.690 |
| ukb-b-4982 | Willing to confide | 85 | 0.94 (0.83-1.05) | 0.277 | -1.56E-03 | 6.46E-03 | 0.810 | 0.953 | 64.011 | 84 | 0.949 |
| ukb-b-5076 | Fewer leisure social activities | 83 | 1.41 (0.76-2.61) | 0.274 | 2.66E-03 | 6.46E-03 | 0.681 | 0.2 | 93.762 | 82 | 0.176 |
| ukb-b-4667 | More activities-Religious group | 157 | 0.85 (0.53-1.37) | 0.499 | -6.64E-03 | 4.72E-03 | 0.161 | 0.957 | 124.985 | 156 | 0.968 |
| ukb-b-4171 | More activities-Pub or social club | 89 | 0.84 (0.50-1.42) | 0.517 | 3.51E-03 | 4.82E-03 | 0.469 | 0.755 | 78.809 | 88 | 0.748 |
| ukb-b-4077 | More activities-Other group activity | 48 | 0.63 (0.29-1.35) | 0.235 | 6.40E-03 | 7.06E-03 | 0.369 | 0.886 | 36.554 | 47 | 0.864 |
| ukb-b-4000 | More activities-Sports club or gym | 63 | 0.46 (0.24-0.89) | 0.021 | -5.73E-03 | 7.12E-03 | 0.424 | 0.497 | 61.578 | 62 | 0.491 |
| ukb-b-1553 | More activities-Adult education class | 31 | 0.70 (0.17-2.90) | 0.619 | 1.62E-03 | 8.30E-03 | 0.846 | 0.931 | 19.240 | 30 | 0.935 |

Abbreviations: GWAS, genome-wide association studies; IBD, inflammatory bowel disease; IVW, inverse-variance weighted; MR, Mendelian randomization.

**Supplementary Table S21.** MR analysis for association between social isolation/loneliness and CD, MR-Egger intercept test for horizontal pleiotropy, and heterogeneity tests with Cochran’s Q statistic.

| **Exposure** |  | **No. of SNPs** | **Random-effects IVW Estimates** | | **MR-Egger Tests** | | | **MR-PRESSO global test** | **Heterogeneity Tests** | | |
| --- | --- | --- | --- | --- | --- | --- | --- | --- | --- | --- | --- |
|  |  |  | **OR (95% CI)** | **P value** | **Intercept** | **std. error** | **P value** | **P value** | **Q value** | **df** | **P value** |
| ukb-b-8476 | Feeling lonely | 81 | 1.27 (0.57-2.82) | 0.554 | -1.42E-03 | 8.59E-03 | 0.869 | 0.839 | 67.607 | 80 | 0.837 |
| ukb-b-5445 | Not living alone | 19 | 1.03 (0.48-2.22) | 0.933 | -1.01E-02 | 1.24E-02 | 0.426 | 0.912 | 10.316 | 18 | 0.921 |
| ukb-b-5379 | More contact with friends or family | 105 | 0.87 (0.69-1.09) | 0.225 | -8.98E-03 | 6.21E-03 | 0.152 | 0.943 | 80.794 | 104 | 0.955 |
| ukb-b-4982 | Willing to confide | 83 | 0.86 (0.73-1.00) | 0.051 | 1.56E-02 | 8.36E-03 | 0.066 | 0.937 | 64.140 | 82 | 0.928 |
| ukb-b-5076 | Fewer leisure social activities | 85 | 1.59 (0.82-3.08) | 0.171 | 5.25E-03 | 7.54E-03 | 0.488 | 0.896 | 68.066 | 84 | 0.897 |
| ukb-b-4667 | More activities-Religious group | 157 | 1.03 (0.55-1.92) | 0.933 | -8.84E-03 | 6.01E-03 | 0.143 | 0.961 | 125.641 | 156 | 0.965 |
| ukb-b-4171 | More activities-Pub or social club | 90 | 1.03 (0.52-2.05) | 0.939 | -3.02E-03 | 6.19E-03 | 0.627 | 0.675 | 83.671 | 89 | 0.640 |
| ukb-b-4077 | More activities-Other group activity | 49 | 0.43 (0.16-1.11) | 0.082 | 1.25E-02 | 9.26E-03 | 0.183 | 0.893 | 35.972 | 48 | 0.900 |
| ukb-b-4000 | More activities-Sports club or gym | 63 | 0.38 (0.16-0.90) | 0.028 | 1.11E-04 | 9.57E-03 | 0.991 | 0.391 | 65.011 | 62 | 0.372 |
| ukb-b-1553 | More activities-Adult education class | 32 | 0.40 (0.05-2.97) | 0.373 | 1.06E-03 | 1.05E-02 | 0.920 | 0.803 | 24.791 | 31 | 0.777 |

Abbreviations: CD, Crohn’s disease; GWAS, genome-wide association studies; IVW, inverse-variance weighted; MR, Mendelian randomization.

**Supplementary Table S22.** MR analysis for association between social isolation/loneliness and UC, MR-Egger intercept test for horizontal pleiotropy, and heterogeneity tests with Cochran’s Q statistic.

| **Exposure** |  | **No. of SNPs** | **Random-effects IVW Estimates** | |  | **MR-Egger Tests** | | |  | **MR-PRESSO global test** | **Heterogeneity Tests** | | |
| --- | --- | --- | --- | --- | --- | --- | --- | --- | --- | --- | --- | --- | --- |
|  |  |  | **OR (95% CI)** | **P value** |  | **Intercept** | **std. error** | **P value** |  | **P value** | **Q value** | **df** | **P value** |
| ukb-b-8476 | Feeling lonely | 82 | 0.47 (0.22-1.00) | 0.050 |  | 1.08E-02 | 8.76E-03 | 0.222 |  | 0.907 | 64.522 | 81 | 0.910 |
| ukb-b-5445 | Not living alone | 20 | 1.12 (0.43-2.92) | 0.810 |  | 9.00E-04 | 1.29E-02 | 0.945 |  | 0.554 | 17.795 | 19 | 0.536 |
| ukb-b-5379 | More contact with friends or family | 100 | 0.95 (0.73-1.24) | 0.722 |  | -1.10E-02 | 6.18E-03 | 0.078 |  | 0.518 | 97.937 | 99 | 0.511 |
| ukb-b-4982 | Willing to confide | 91 | 1.03 (0.89-1.18) | 0.723 |  | 4.10E-04 | 7.52E-03 | 0.957 |  | 0.967 | 66.379 | 90 | 0.971 |
| ukb-b-5076 | Fewer leisure social activities | 84 | 3.00 (1.44-6.22) | 0.003 |  | -2.31E-03 | 8.11E-03 | 0.777 |  | 0.441 | 84.424 | 83 | 0.436 |
| ukb-b-4667 | More activities-Religious group | 159 | 0.55 (0.32-0.94) | 0.031 |  | -2.68E-03 | 5.92E-03 | 0.652 |  | 1.000 | 103.502 | 158 | 1.000 |
| ukb-b-4171 | More activities-Pub or social club | 92 | 0.93 (0.47-1.84) | 0.846 |  | 1.86E-03 | 6.11E-03 | 0.762 |  | 0.555 | 88.272 | 91 | 0.561 |
| ukb-b-4077 | More activities-Other group activity | 50 | 0.64 (0.26-1.62) | 0.351 |  | 4.56E-03 | 9.06E-03 | 0.617 |  | 0.929 | 36.027 | 49 | 0.916 |
| ukb-b-4000 | More activities-Sports club or gym | 65 | 0.98 (0.49-1.98) | 0.957 |  | -1.09E-02 | 9.08E-03 | 0.234 |  | 0.942 | 46.338 | 64 | 0.953 |
| ukb-b-1553 | More activities-Adult education class | 32 | 0.36 (0.05-2.95) | 0.345 |  | 3.13E-03 | 1.05E-02 | 0.768 |  | 0.664 | 27.386 | 31 | 0.653 |

Abbreviations: GWAS, genome-wide association studies; IVW, inverse-variance weighted; MR, Mendelian randomization; UC, ulcerative colitis.

**Supplementary Table S23.** Sensitivity MR analyses for association between social isolation/loneliness and IBD.

| **Exposure** | **Method** | **Number of SNPs** | **OR (95%CI)** | **P value** |
| --- | --- | --- | --- | --- |
| ukb-b-1553 | MRE-IVW | 31 | 0.70 (0.17-2.90) | 0.619 |
| ukb-b-1553 | FE-IVW | 31 | 0.70 (0.12-4.13) | 0.691 |
| ukb-b-1553 | Weighted median | 31 | 1.19 (0.09-14.93) | 0.893 |
| ukb-b-1553 | Penalised weighted median | 31 | 1.19 (0.10-14.23) | 0.891 |
| ukb-b-1553 | MR Egger | 31 | 0.46 (0.01-40.26) | 0.738 |
| ukb-b-4000 | MRE-IVW | 63 | 0.46 (0.24-0.89) | 0.021 |
| ukb-b-4000 | FE-IVW | 63 | 0.46 (0.24-0.89) | 0.022 |
| ukb-b-4000 | Weighted median | 63 | 0.42 (0.17-1.09) | 0.075 |
| ukb-b-4000 | Penalised weighted median | 63 | 0.42 (0.16-1.10) | 0.078 |
| ukb-b-4000 | MR Egger | 63 | 1.22 (0.10-14.35) | 0.873 |
| ukb-b-4077 | MRE-IVW | 48 | 0.63 (0.29-1.35) | 0.235 |
| ukb-b-4077 | FE-IVW | 48 | 0.63 (0.26-1.50) | 0.294 |
| ukb-b-4077 | Weighted median | 48 | 0.48 (0.14-1.57) | 0.222 |
| ukb-b-4077 | Penalised weighted median | 48 | 0.46 (0.13-1.61) | 0.228 |
| ukb-b-4077 | MR Egger | 48 | 0.20 (0.01-2.81) | 0.237 |
| ukb-b-4171 | MRE-IVW | 89 | 0.84 (0.50-1.42) | 0.517 |
| ukb-b-4171 | FE-IVW | 89 | 0.84 (0.49-1.46) | 0.540 |
| ukb-b-4171 | Weighted median | 89 | 0.74 (0.31-1.79) | 0.509 |
| ukb-b-4171 | Penalised weighted median | 89 | 0.74 (0.31-1.80) | 0.511 |
| ukb-b-4171 | MR Egger | 89 | 0.49 (0.10-2.32) | 0.372 |
| ukb-b-4667 | MRE-IVW | 157 | 0.85 (0.53-1.37) | 0.499 |
| ukb-b-4667 | FE-IVW | 157 | 0.85 (0.50-1.45) | 0.545 |
| ukb-b-4667 | Weighted median | 157 | 0.76 (0.37-1.59) | 0.471 |
| ukb-b-4667 | Penalised weighted median | 157 | 0.76 (0.36-1.63) | 0.486 |
| ukb-b-4667 | MR Egger | 157 | 3.61 (0.45-28.96) | 0.230 |
| ukb-b-4982 | MRE-IVW | 85 | 0.94 (0.83-1.05) | 0.277 |
| ukb-b-4982 | FE-IVW | 85 | 0.94 (0.82-1.07) | 0.342 |
| ukb-b-4982 | Weighted median | 85 | 0.90 (0.74-1.09) | 0.273 |
| ukb-b-4982 | Penalised weighted median | 85 | 0.90 (0.75-1.08) | 0.260 |
| ukb-b-4982 | MR Egger | 85 | 1.00 (0.59-1.70) | 0.996 |
| ukb-b-5076 | MRE-IVW | 83 | 1.41 (0.76-2.61) | 0.274 |
| ukb-b-5076 | FE-IVW | 83 | 1.41 (0.79-2.51) | 0.242 |
| ukb-b-5076 | Weighted median | 83 | 1.40 (0.59-3.32) | 0.445 |
| ukb-b-5076 | Penalised weighted median | 83 | 1.40 (0.57-3.43) | 0.462 |
| ukb-b-5076 | MR Egger | 83 | 0.92 (0.11-7.72) | 0.938 |
| ukb-b-5379 | MRE-IVW | 102 | 0.87 (0.72-1.06) | 0.181 |
| ukb-b-5379 | FE-IVW | 102 | 0.87 (0.71-1.07) | 0.198 |
| ukb-b-5379 | Weighted median | 102 | 0.74 (0.55-0.99) | 0.044 |
| ukb-b-5379 | Penalised weighted median | 102 | 0.74 (0.55-1.00) | 0.049 |
| ukb-b-5379 | MR Egger | 102 | 1.76 (0.93-3.33) | 0.087 |
| ukb-b-5445 | MRE-IVW | 20 | 0.93 (0.49-1.76) | 0.829 |
| ukb-b-5445 | FE-IVW | 20 | 0.93 (0.43-2.02) | 0.859 |
| ukb-b-5445 | Weighted median | 20 | 0.93 (0.33-2.68) | 0.900 |
| ukb-b-5445 | Penalised weighted median | 20 | 0.93 (0.31-2.77) | 0.903 |
| ukb-b-5445 | MR Egger | 20 | 2.53 (0.36-17.85) | 0.365 |
| ukb-b-8476 | MRE-IVW | 79 | 0.82 (0.46-1.44) | 0.484 |
| ukb-b-8476 | FE-IVW | 79 | 0.82 (0.41-1.61) | 0.558 |
| ukb-b-8476 | Weighted median | 79 | 0.90 (0.34-2.38) | 0.827 |
| ukb-b-8476 | Penalised weighted median | 79 | 0.90 (0.34-2.36) | 0.825 |
| ukb-b-8476 | MR Egger | 79 | 0.97 (0.06-14.86) | 0.983 |

Abbreviations: CI, confidence interval; IBD, inflammatory bowel disease; IVW, inverse-variance weighted; MR, Mendelian randomization; OR, odds ratio.

**Supplementary Table S24.** Sensitivity MR analyses for association between social isolation or loneliness and CD.

| **Exposure** | **Method** | **Number of SNPs** | **OR (95%CI)** | **P value** |
| --- | --- | --- | --- | --- |
| ukb-b-1553 | MRE-IVW | 32 | 0.40 (0.05-2.97) | 0.373 |
| ukb-b-1553 | FE-IVW | 32 | 0.40 (0.04-3.77) | 0.426 |
| ukb-b-1553 | Weighted median | 32 | 0.66 (0.03-14.38) | 0.790 |
| ukb-b-1553 | Penalised weighted median | 32 | 0.66 (0.03-15.13) | 0.793 |
| ukb-b-1553 | MR Egger | 32 | 0.31 (0.00-89.69) | 0.687 |
| ukb-b-4000 | MRE-IVW | 63 | 0.38 (0.16-0.90) | 0.028 |
| ukb-b-4000 | FE-IVW | 63 | 0.38 (0.16-0.88) | 0.025 |
| ukb-b-4000 | Weighted median | 63 | 0.34 (0.10-1.17) | 0.087 |
| ukb-b-4000 | Penalised weighted median | 63 | 0.32 (0.09-1.09) | 0.069 |
| ukb-b-4000 | MR Egger | 63 | 0.37 (0.01-9.92) | 0.556 |
| ukb-b-4077 | MRE-IVW | 49 | 0.43 (0.16-1.11) | 0.082 |
| ukb-b-4077 | FE-IVW | 49 | 0.43 (0.14-1.29) | 0.132 |
| ukb-b-4077 | Weighted median | 49 | 0.57 (0.12-2.71) | 0.478 |
| ukb-b-4077 | Penalised weighted median | 49 | 0.57 (0.11-2.82) | 0.489 |
| ukb-b-4077 | MR Egger | 49 | 0.04 (0.00-1.44) | 0.085 |
| ukb-b-4171 | MRE-IVW | 90 | 1.03 (0.52-2.05) | 0.939 |
| ukb-b-4171 | FE-IVW | 90 | 1.03 (0.50-2.09) | 0.941 |
| ukb-b-4171 | Weighted median | 90 | 1.12 (0.34-3.65) | 0.849 |
| ukb-b-4171 | Penalised weighted median | 90 | 1.12 (0.35-3.55) | 0.846 |
| ukb-b-4171 | MR Egger | 90 | 1.65 (0.22-12.47) | 0.630 |
| ukb-b-4667 | MRE-IVW | 157 | 1.03 (0.55-1.92) | 0.933 |
| ukb-b-4667 | FE-IVW | 157 | 1.03 (0.51-2.06) | 0.940 |
| ukb-b-4667 | Weighted median | 157 | 0.93 (0.34-2.55) | 0.883 |
| ukb-b-4667 | Penalised weighted median | 157 | 0.93 (0.35-2.46) | 0.879 |
| ukb-b-4667 | MR Egger | 157 | 7.07 (0.49-101.21) | 0.152 |
| ukb-b-4982 | MRE-IVW | 83 | 0.86 (0.73-1.00) | 0.051 |
| ukb-b-4982 | FE-IVW | 83 | 0.86 (0.72-1.02) | 0.085 |
| ukb-b-4982 | Weighted median | 83 | 0.83 (0.65-1.06) | 0.142 |
| ukb-b-4982 | Penalised weighted median | 83 | 0.83 (0.65-1.07) | 0.160 |
| ukb-b-4982 | MR Egger | 83 | 0.46 (0.23-0.91) | 0.028 |
| ukb-b-5076 | MRE-IVW | 85 | 1.59 (0.82-3.08) | 0.171 |
| ukb-b-5076 | FE-IVW | 85 | 1.59 (0.76-3.31) | 0.218 |
| ukb-b-5076 | Weighted median | 85 | 1.48 (0.50-4.40) | 0.484 |
| ukb-b-5076 | Penalised weighted median | 85 | 1.48 (0.51-4.29) | 0.474 |
| ukb-b-5076 | MR Egger | 85 | 0.69 (0.06-8.05) | 0.769 |
| ukb-b-5379 | MRE-IVW | 105 | 0.87 (0.69-1.09) | 0.225 |
| ukb-b-5379 | FE-IVW | 105 | 0.87 (0.67-1.13) | 0.285 |
| ukb-b-5379 | Weighted median | 105 | 0.92 (0.64-1.34) | 0.673 |
| ukb-b-5379 | Penalised weighted median | 105 | 0.92 (0.63-1.35) | 0.682 |
| ukb-b-5379 | MR Egger | 105 | 1.56 (0.67-3.60) | 0.301 |
| ukb-b-5445 | MRE-IVW | 19 | 1.03 (0.48-2.22) | 0.933 |
| ukb-b-5445 | FE-IVW | 19 | 1.03 (0.38-2.84) | 0.949 |
| ukb-b-5445 | Weighted median | 19 | 0.68 (0.18-2.57) | 0.571 |
| ukb-b-5445 | Penalised weighted median | 19 | 0.68 (0.17-2.66) | 0.581 |
| ukb-b-5445 | MR Egger | 19 | 2.66 (0.22-31.96) | 0.452 |
| ukb-b-8476 | MRE-IVW | 81 | 1.27 (0.57-2.82) | 0.554 |
| ukb-b-8476 | FE-IVW | 81 | 1.27 (0.54-3.02) | 0.586 |
| ukb-b-8476 | Weighted median | 81 | 1.08 (0.30-3.82) | 0.909 |
| ukb-b-8476 | Penalised weighted median | 81 | 1.08 (0.30-3.84) | 0.909 |
| ukb-b-8476 | MR Egger | 81 | 1.67 (0.06-48.81) | 0.765 |

Abbreviations: CD, Crohn’s disease; CI, confidence interval; IVW, inverse-variance weighted; MR, Mendelian randomization; OR, odds ratio.

**Supplementary Table S25.** Sensitivity MR analyses for association between social isolation or loneliness and UC.

| **Exposure** | **Method** | **Number of SNPs** | **OR (95%CI)** | **P value** |
| --- | --- | --- | --- | --- |
| ukb-b-1553 | MRE-IVW | 32 | 0.36 (0.05-2.95) | 0.345 |
| ukb-b-1553 | FE-IVW | 32 | 0.36 (0.04-3.38) | 0.374 |
| ukb-b-1553 | Weighted median | 32 | 0.84 (0.03-20.31) | 0.914 |
| ukb-b-1553 | Penalised weighted median | 32 | 0.84 (0.04-19.87) | 0.913 |
| ukb-b-1553 | MR Egger | 32 | 0.16 (0.00-49.39) | 0.540 |
| ukb-b-4000 | MRE-IVW | 65 | 0.98 (0.49-1.98) | 0.957 |
| ukb-b-4000 | FE-IVW | 65 | 0.98 (0.43-2.24) | 0.964 |
| ukb-b-4000 | Weighted median | 65 | 1.03 (0.33-3.27) | 0.953 |
| ukb-b-4000 | Penalised weighted median | 65 | 1.03 (0.32-3.36) | 0.955 |
| ukb-b-4000 | MR Egger | 65 | 6.11 (0.28-134.90) | 0.256 |
| ukb-b-4077 | MRE-IVW | 50 | 0.64 (0.26-1.62) | 0.351 |
| ukb-b-4077 | FE-IVW | 50 | 0.64 (0.22-1.89) | 0.423 |
| ukb-b-4077 | Weighted median | 50 | 0.52 (0.12-2.37) | 0.400 |
| ukb-b-4077 | Penalised weighted median | 50 | 0.52 (0.11-2.38) | 0.401 |
| ukb-b-4077 | MR Egger | 50 | 0.28 (0.01-8.33) | 0.468 |
| ukb-b-4171 | MRE-IVW | 92 | 0.93 (0.47-1.84) | 0.846 |
| ukb-b-4171 | FE-IVW | 92 | 0.93 (0.47-1.86) | 0.848 |
| ukb-b-4171 | Weighted median | 92 | 1.45 (0.50-4.19) | 0.496 |
| ukb-b-4171 | Penalised weighted median | 92 | 1.45 (0.52-4.04) | 0.480 |
| ukb-b-4171 | MR Egger | 92 | 0.70 (0.10-5.10) | 0.726 |
| ukb-b-4667 | MRE-IVW | 159 | 0.55 (0.32-0.94) | 0.031 |
| ukb-b-4667 | FE-IVW | 159 | 0.55 (0.28-1.07) | 0.080 |
| ukb-b-4667 | Weighted median | 159 | 0.50 (0.20-1.26) | 0.140 |
| ukb-b-4667 | Penalised weighted median | 159 | 0.50 (0.20-1.25) | 0.136 |
| ukb-b-4667 | MR Egger | 159 | 0.98 (0.07-13.43) | 0.987 |
| ukb-b-4982 | MRE-IVW | 91 | 1.03 (0.89-1.18) | 0.723 |
| ukb-b-4982 | FE-IVW | 91 | 1.03 (0.87-1.21) | 0.761 |
| ukb-b-4982 | Weighted median | 91 | 1.08 (0.86-1.36) | 0.501 |
| ukb-b-4982 | Penalised weighted median | 91 | 1.08 (0.85-1.37) | 0.514 |
| ukb-b-4982 | MR Egger | 91 | 1.01 (0.55-1.86) | 0.976 |
| ukb-b-5076 | MRE-IVW | 84 | 3.00 (1.44-6.22) | 0.003 |
| ukb-b-5076 | FE-IVW | 84 | 3.00 (1.45-6.18) | 0.003 |
| ukb-b-5076 | Weighted median | 84 | 3.81 (1.34-10.79) | 0.012 |
| ukb-b-5076 | Penalised weighted median | 84 | 3.83 (1.31-11.19) | 0.014 |
| ukb-b-5076 | MR Egger | 84 | 4.37 (0.29-65.09) | 0.288 |
| ukb-b-5379 | MRE-IVW | 100 | 0.95 (0.73-1.24) | 0.722 |
| ukb-b-5379 | FE-IVW | 100 | 0.95 (0.73-1.24) | 0.724 |
| ukb-b-5379 | Weighted median | 100 | 0.98 (0.66-1.45) | 0.911 |
| ukb-b-5379 | Penalised weighted median | 100 | 0.98 (0.66-1.46) | 0.912 |
| ukb-b-5379 | MR Egger | 100 | 1.96 (0.85-4.51) | 0.119 |
| ukb-b-5445 | MRE-IVW | 20 | 1.12 (0.43-2.92) | 0.810 |
| ukb-b-5445 | FE-IVW | 20 | 1.12 (0.42-3.01) | 0.816 |
| ukb-b-5445 | Weighted median | 20 | 1.02 (0.25-4.17) | 0.973 |
| ukb-b-5445 | Penalised weighted median | 20 | 1.02 (0.26-4.07) | 0.972 |
| ukb-b-5445 | MR Egger | 20 | 1.03 (0.07-15.09) | 0.984 |
| ukb-b-8476 | MRE-IVW | 82 | 0.47 (0.22-1.00) | 0.050 |
| ukb-b-8476 | FE-IVW | 82 | 0.47 (0.20-1.10) | 0.081 |
| ukb-b-8476 | Weighted median | 82 | 0.40 (0.12-1.37) | 0.147 |
| ukb-b-8476 | Penalised weighted median | 82 | 0.40 (0.12-1.40) | 0.154 |
| ukb-b-8476 | MR Egger | 82 | 0.06 (0.00-1.79) | 0.108 |

Abbreviations: CI, confidence interval; IVW, inverse-variance weighted; MR, Mendelian randomization; OR, odds ratio; UC, ulcerative colitis.

**Supplementary Table S26.** Reverse MR analysis with random-effects IVW for association between IBD, CD and UC and social isolation/loneliness.

| **Outcome id** | **Outcome** | **Exposure** | **Method** | **nsnp** | **b** | **se** | **P value** | **OR (95%CI)** |
| --- | --- | --- | --- | --- | --- | --- | --- | --- |
| ukb-b-1553 | Leisure/social activities: Adult education class \|\| id:ukb-b-1553 | CD | IVW | 56 | 0.000 | 0.000 | 0.720 | 1.000 (0.999-1.001) |
| ukb-b-4000 | Leisure/social activities: Sports club or gym \|\| id:ukb-b-4000 | CD | IVW | 57 | -0.002 | 0.001 | 0.237 | 0.998 (0.996-1.001) |
| ukb-b-4077 | Leisure/social activities: Other group activity \|\| id:ukb-b-4077 | CD | IVW | 56 | 0.001 | 0.001 | 0.357 | 1.001 (0.999-1.003) |
| ukb-b-4171 | Leisure/social activities: Pub or social club \|\| id:ukb-b-4171 | CD | IVW | 57 | -0.001 | 0.001 | 0.274 | 0.999 (0.996-1.001) |
| ukb-b-4667 | Leisure/social activities: Religious group \|\| id:ukb-b-4667 | CD | IVW | 56 | 0.000 | 0.001 | 0.780 | 1.000 (0.998-1.002) |
| ukb-b-4982 | Able to confide \|\| id:ukb-b-4982 | CD | IVW | 57 | -0.003 | 0.004 | 0.433 | 0.997 (0.988-1.005) |
| ukb-b-5076 | Leisure/social activities: None of the above \|\| id:ukb-b-5076 | CD | IVW | 56 | 0.002 | 0.001 | 0.166 | 1.002 (0.999-1.004) |
| ukb-b-5379 | Frequency of friend/family visits \|\| id:ukb-b-5379 | CD | IVW | 57 | 0.002 | 0.003 | 0.386 | 1.002 (0.997-1.007) |
| ukb-b-5445 | Number in household \|\| id:ukb-b-5445 | CD | IVW | 57 | -0.003 | 0.001 | 0.046 | 0.997 (0.995-1.000) |
| ukb-b-8476 | Loneliness, isolation \|\| id:ukb-b-8476 | CD | IVW | 57 | 0.001 | 0.001 | 0.204 | 1.001 (0.999-1.003) |
| ukb-b-1553 | Leisure/social activities: Adult education class \|\| id:ukb-b-1553 | IBD | IVW | 77 | 0.000 | 0.001 | 0.464 | 1.000 (0.998-1.001) |
| ukb-b-4000 | Leisure/social activities: Sports club or gym \|\| id:ukb-b-4000 | IBD | IVW | 77 | -0.001 | 0.001 | 0.345 | 0.999 (0.997-1.001) |
| ukb-b-4077 | Leisure/social activities: Other group activity \|\| id:ukb-b-4077 | IBD | IVW | 77 | -0.001 | 0.001 | 0.503 | 0.999 (0.997-1.001) |
| ukb-b-4171 | Leisure/social activities: Pub or social club \|\| id:ukb-b-4171 | IBD | IVW | 77 | 0.001 | 0.001 | 0.626 | 1.001 (0.998-1.003) |
| ukb-b-4667 | Leisure/social activities: Religious group \|\| id:ukb-b-4667 | IBD | IVW | 77 | 0.000 | 0.001 | 0.886 | 1.000 (0.998-1.002) |
| ukb-b-4982 | Able to confide \|\| id:ukb-b-4982 | IBD | IVW | 78 | 0.004 | 0.005 | 0.476 | 1.004 (0.993-1.014) |
| ukb-b-5076 | Leisure/social activities: None of the above \|\| id:ukb-b-5076 | IBD | IVW | 77 | 0.000 | 0.001 | 0.793 | 1.000 (0.998-1.003) |
| ukb-b-5379 | Frequency of friend/family visits \|\| id:ukb-b-5379 | IBD | IVW | 78 | -0.005 | 0.003 | 0.123 | 0.995 (0.988-1.001) |
| ukb-b-5445 | Number in household \|\| id:ukb-b-5445 | IBD | IVW | 78 | -0.002 | 0.002 | 0.292 | 0.998 (0.994-1.002) |
| ukb-b-8476 | Loneliness, isolation \|\| id:ukb-b-8476 | IBD | IVW | 77 | 0.001 | 0.001 | 0.560 | 1.001 (0.998-1.003) |
| ukb-b-1553 | Leisure/social activities: Adult education class \|\| id:ukb-b-1553 | UC | IVW | 45 | 0.002 | 0.001 | 0.007 | 1.002 (1.000-1.003) |
| ukb-b-4000 | Leisure/social activities: Sports club or gym \|\| id:ukb-b-4000 | UC | IVW | 45 | 0.000 | 0.001 | 0.871 | 1.000 (0.997-1.003) |
| ukb-b-4077 | Leisure/social activities: Other group activity \|\| id:ukb-b-4077 | UC | IVW | 45 | 0.001 | 0.001 | 0.457 | 1.001 (0.999-1.003) |
| ukb-b-4171 | Leisure/social activities: Pub or social club \|\| id:ukb-b-4171 | UC | IVW | 45 | 0.000 | 0.001 | 0.870 | 1.000 (0.997-1.002) |
| ukb-b-4667 | Leisure/social activities: Religious group \|\| id:ukb-b-4667 | UC | IVW | 45 | 0.001 | 0.001 | 0.415 | 1.001 (0.999-1.003) |
| ukb-b-4982 | Able to confide \|\| id:ukb-b-4982 | UC | IVW | 45 | 0.001 | 0.004 | 0.749 | 1.001 (0.993-1.010) |
| ukb-b-5076 | Leisure/social activities: None of the above \|\| id:ukb-b-5076 | UC | IVW | 45 | -0.002 | 0.001 | 0.191 | 0.998 (0.995-1.001) |
| ukb-b-5379 | Frequency of friend/family visits \|\| id:ukb-b-5379 | UC | IVW | 45 | 0.000 | 0.003 | 0.987 | 1.000 (0.994-1.006) |
| ukb-b-5445 | Number in household \|\| id:ukb-b-5445 | UC | IVW | 45 | -0.001 | 0.002 | 0.572 | 0.999 (0.995-1.003) |
| ukb-b-8476 | Loneliness, isolation \|\| id:ukb-b-8476 | UC | IVW | 45 | 0.001 | 0.001 | 0.346 | 1.001 (0.999-1.003) |

Abbreviations: CD, Crohn’s disease; CI, confidence interval; IBD, inflammatory bowel disease; IVW, inverse-variance weighted; MR, Mendelian randomization; OR, odds ratio; UC, ulcerative colitis.

**Reference:**

1 Elovainio, M. *et al.* Association of social isolation and loneliness with risk of incident hospital-treated infections: an analysis of data from the UK Biobank and Finnish Health and Social Support studies. *LANCET PUBLIC HEALTH* **8**, e109-e118, doi:10.1016/s2468-2667(22)00253-5 (2023).
